# *Lycium barbarum* Extract Enhanced Neuroplasticity and Functional Recovery in 5xFAD Mice via Modulating Microglial Status of the Central Nervous System

**DOI:** 10.1111/cns.70123

**Published:** 2024-11-20

**Authors:** Zhongqing Sun, Jinfeng Liu, Zihang Chen, Kwok‐Fai So, Yong Hu, Kin Chiu

**Affiliations:** ^1^ Department of Neurology, Xijing Hospital Fourth Military Medical University Xi'an China; ^2^ Innovation Research Institute, Xijing Hospital Fourth Military Medical University Xi'an China; ^3^ Department of Ophthalmology, School of Clinical Medicine The University of Hong Kong Hong Kong SAR China; ^4^ Department of Orthopaedics and Traumatology, School of Clinical Medicine, Li Kai Shing Faculty of Medicine The University of Hong Kong Hong Kong SAR China; ^5^ Department of Psychology The University of Hong Kong Hong Kong SAR China; ^6^ Department of Sports Medicine, the First Affiliated Hospital Jinan University China; ^7^ State Key Lab of Brain and Cognitive Sciences The University of Hong Kong Hong Kong SAR China; ^8^ Key Laboratory of CNS Regeneration, Guangdong‐Hongkong‐Macau CNS Regeneration Institute, Ministry of Education Jinan University Guangzhou China; ^9^ Orthopedics Center The University of Hong Kong‐Shenzhen Hospital Shenzhen China

**Keywords:** 5xFAD mice, Alzheimer's pathology, central nervous system, *Lycium barbarum* extract, neuroinflammation, neuroprotective microglial phenotype, retina, spinal cord

## Abstract

**Objective:**

Alzheimer's disease (AD) is the most prevalent neurodegenerative disease with limited treatment options. This study aimed to investigate the effects of *Lycium barbarum* extract (LBE), a Chinese herb, on the central nervous system (CNS)—including the retina, brain, and spinal cord—in 5xFAD transgenic mice after the onset of AD.

**Methods:**

Starting at 6 months of age, 5xFAD mice received daily intragastric gavage of LBE (2 g/kg) for 2 months. At 8 months, behavioral tests were conducted to assess cognition, motor function, and visual function. These included the Morris water maze, novel object recognition, and Y‐maze tests for cognition; the beam walking balance and clasping tests for motor function; and electroretinogram (ERG) for visual function. Immunohistochemistry, western blotting, and ELISA were used to evaluate Aβ deposition, microglial morphology, neuroinflammation, and neuroprotective signaling pathways. Primary microglia and the IMG cell line were used to study LBE's effects on Aβ uptake and degradation in vitro.

**Results:**

After 2 months of LBE treatment, the decline in cognition, motor, and visual functions in 5xFAD mice was significantly slowed. Microglia in the brain, spinal cord, and retina exhibited a neuroprotective state, with reduced Aβ deposition, decreased inflammatory cytokine levels (e.g., TNF‐α, IL‐1β, IL‐6), increased Arg‐1/iNOS ratio, and enhanced phagocytic capacity. LBE also promoted Aβ uptake and degradation in primary microglia and the IMG cell line. Neuroprotective signals such as p‐Akt, p‐Erk1/2, and p‐CREB were elevated. Additionally, LBE treatment restored synaptic protein expression and enhanced neuroplasticity.

**Conclusion:**

The findings suggest that LBE treatment can enhance neuroplasticity, reduce systemic inflammation, and improve phagocyte clearance of Aβ deposition via inducing a neuroprotective microglial phenotype throughout CNS. As an upper‐class Chinese medicine, appropriate intake of LBE may serve as a beneficial antiaging strategy for AD.

## Graphical abstract

LBE administration preserved cognition, movement, and visual function in 5xFAD mice. It reversed systemic inflammation and reduced Aβ load by enhancing microglial phagocytosis through modulating microglia into a neuroprotective state, thereby ameliorating the neurological pathology of the CNS (brain, spinal cord, and retina).

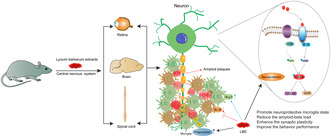

## 1 Introduction

Alzheimer's disease (AD) is the primary cause of dementia and poses a significant healthcare challenge for families and society [1, 2]. In China, the prevalence of dementia accounts for approximately 25% of the global dementia population, exceeding 10 million [3], yet effective treatments for AD remain limited. The pathological hallmarks of AD in the brain include extracellular Aβ plaques, hyperphosphorylated tau tangles, chronic neuroinflammation, and massive neural cell loss [2]. Although significant advances have been in understanding the pathology of AD, there are few disease‐modifying therapies available in clinical trials. Recently, two monoclonal antibodies (Aducanumab and Lecanemab) have been approved for clinical use with preliminary effects [4], but their high cost and potential side effects may limit their widespread application.

Aβ deposition drives chronic neuroinflammation by stimulating microglia, which is strongly associated with the development of AD. Activated microglia exhibit reduced neurotrophic secretion, weakened phagocytosis, and increased expression of inflammatory mediators, including TNF‐α, IL‐6, and IL‐1β [5, 6]. This inflammatory response is observed not only in the brain but also in the spinal cord and retina [7, 8, 9]. In the spinal cord, Aβ‐activated microglia contribute to the axonopathy and motor impairment [8]. In the retina, Aβ deposition, retinal ganglion cell loss, NFL atrophy, and thinning of the macular ganglion cell complex have been observed in postmortem retinas of AD patients at various stages [10]. In transgenic AD mice, Aβ deposition is associated with neuroinflammation, visual function impairments, and loss of retinal ganglion cells (RGC) [11, 12].

*Lycium barbarum* (LB; also known as goji berry, wolfberry, or gouqizi in Chinese), a traditional Chinese medicine, has been used for thousands of years to improve vision and nourish the liver and kidney by balancing “yin” and “yang” in the body [13]. LB contains various components, such as polysaccharides, betaine, taurine, cerebroside, vitamin, flavonoids, and fatty acids, which exhibit immunomodulatory, antioxidant, and neuroprotective properties [13, 14]. Studies have shown beneficial effects of LB extracts/LB polysaccharide (LBP) in stroke, spinal cord injury (SCI), glaucoma, and age‐related macular degeneration. LBP also preserves the cognitive functions and attenuate neuropathology of AD transgenic mice [15, 16, 17, 18]. Additionally, LBP exhibits an anti‐inflammatory effect, reducing the production of inflammatory mediators partly through inhibition of NF‐κB in the liver injury models [19, 20]. Posttreatment with LBP significantly increases the ratio of M2/M1 microglia/macrophages and promotes M2 polarization (anti‐inflammatory) activity in the SCI [21]. In our previous study, LBE promoted the activation of microglia with anti‐inflammatory character and reduced oligomeric Aβ‐induced inflammatory reactions in a microglia cell line [22]. It also preserved retinal function through synaptic stabilization in 3xTg mice when used as a preventative agent [23]. These findings encouraged us to comprehensively investigate the effect of LBE on CNS of AD mice.

In this study, we aimed to investigate the efficacy of 2 months of oral LBE administration in 5xFAD mice. We assessed behavioral performance, Aβ deposition, and microglial status in CNS, including brain, spinal cord, and retina. The study period covered the major progression stages of AD in 5xFAD mice, providing encouragement for the future clinical application of LBE as a treatment for AD patients.

## 2 Methods

### 2.1 Animals

Transgenic 5xFAD mice were obtained from Jackson Laboratory (stock no: 34848) without retina degeneration allele Pde6b^rd1^. These mice overexpress family Alzheimer's disease mutations of human APP (695) with the Swedish mutation (K670N/M671L), Florida mutation (I716V), and London mutation (V717I), and human PS1 harboring two FAD mutations, M146L and L286V, resulting in abundant production of amyloid plaques in the cerebral cortex, hippocampus, spinal cord, and retina. C57BL/6J mice were purchased from Laboratory Animal Unit of the University of Hong Kong to maintain the colony by breeding with 5xFAD mice. Hemizygous 5xFAD mice and nontransgenic wild‐type littermates were used. Mice were housed in groups no more than five on a standard 12‐h light/12‐h dark cycle and were supplied with regular feed and water. For all experiments, mice from the same litter were divided into different conditions accordingly. Mice handling and all other procedures were conducted in accordance with the National Institutes of Health guide for the care and use of laboratory animals and the Animals (Control of experiments) Ordinance, Hong Kong, China. The use of animals was approved by the Committee on the Use of Live Animals in Teaching and Research of the University of Hong Kong (CULATR, no: 5724‐21). All efforts were made to minimize animal numbers and their suffering.

### 2.2 Treatment With LBE

LBE, prepared and provided by Eu Yan Sang (HK) Ltd [22], was derived from dried, mature wolfberry fruit sourced from Zhongning in the Ningxia Hui Autonomous Region, China. The extraction method involved washing 2.5 kg of wolfberry fruit, soaking it in 40°C warm water for 15 min, boiling it for 1 h, and then boiling the filtered residue again. The extracts from both boils were combined and concentrated to 1.25 kg. This LBE extract was weighed and diluted in ultrapure water (w/v) to create a stock solution (100 mg/mL). Before use, the LBE extract was filtered with a 0.22‐μm filter membrane and further diluted in 0.9% saline to a final concentration of 2 mg/mL. Six‐month‐old 5xFAD mice received daily oral dose of LBE (2 g/kg) for 2 months, with water serving as the control. Mice were sacrificed after behavioral assessment.

### 2.3 Cell Culture

Primary microglia were isolated from cerebral cortices of postnatal day 1–3 C57BL/6J wild‐type mouse pups [24]. Meninges were carefully removed, and the dissected cortical tissue was digested using papain at 37°C. The titrated single cell suspension was plated on 100‐mm culture dish in DMEM/F12 medium supplemented with 10% FBS, 20% L‐929 conditioned medium, and penicillin and streptomycin. After 24 h, the medium was changed to eliminate dead cells and myelin debris. On days 10–14, microglia were isolated by mild trypsin treatment and cultured until the cell reached confluence. L‐929 cells (American Type Culture Collection) were cultured in DMEM/F12 medium supplemented with 10% FBS and penicillin and streptomycin antibiotics. L‐929 conditioned medium was collected and filtered using 0.22‐μM syringe filter. The filtered condition medium was stored at −80°C for long‐term use.

The immortalized microglial cell line (IMG) was purchased from Sigma‐Aldrich (SCC134, St. Louis, MO, USA). It is derived from adult mouse brain infected with the v‐raf/v‐myc retrovirus [25]. IMG were cultured in DMEM‐high glucose supplemented with 10% FBS and penicillin and streptomycin.

For confocal imaging, cells at 80% confluence were harvested by scraping, washed with fresh medium, and spun down (500 rpm in Eppendorf microfuge) for cell counting. Cells were then seeded into a 35‐mm confocal culture dish (Mat‐Tek) at a density of about 20 × 10^4^ cells/mL in 2‐mL seeding volume.

### 2.4 Aβ Preparation

Aβ_1‐42_ peptide was purchased from the ERI Amyloid Laboratory (Oxford, CT, USA) and prepared according to a previous protocol [22]. The peptide was initially dissolved to 1 mM in 1,1,1,3,3,3‐hexafluoro‐2‐propanol (HFIP, Sigma‐Aldrich, St. Louis, MO, USA) and left in a biological safety cabinet with lids open overnight to remove the HFIP. The dried peptide was resuspended in DMSO to a concentration of 5 mM and further diluted in F12 medium without red to 100 μM. After incubation for 24 h at 4°C, the Aβ solution was centrifuged at 15,000 × *g* for 10 min at 4°C to harvest the supernatant, which was enriched with oligomeric Aβ_1–42_ and quantitated using a micro‐BCA assay (Thermo, #23325, Waltham, MA, USA). To obtain fibrillar Aβ, 5 mM Aβ_1–42_ in DMSO was diluted to 200 μM in 100 mM HEPES buffer (pH 7.5) and incubated for 1 week at 37°C. After aging, the Aβ sample was centrifuged at 15,000 *g* for 10 min at room temperature and the supernatant was removed. The pellet fraction was redissolved in HEPES buffer and quantitated using the BCA assay. Successful oligomeric Aβ (o‐Aβ) was validated with Tris‐Tricine sodium dodecyl sulfate–polyacrylamide gel electrophoresis (SDS‐PAGE) and western blot [22].

### 2.5 Immunofluorescence Staining for Cell Culture

Primary microglia and IMG cells were treated with untreated or treated with different doses of LBE before being incubated with 5 μM o‐Aβ for 1 and 24 h. After treatment, cells were washed twice and fixed with 4% paraformaldehyde (PFA) for 10 min, blocked with 5% goat serum (Invitrogen, Carlsbad, CA, USA) in PBS, and then incubated with the following primary antibodies: 4G8 (Biolegend, 800,701), Iba‐1(Wako, 019‐19741) overnight at 4°C. The next day, cells were washed with PBS three times and then added the second antibodies for 2 h at room temperature. DAPI was used to counterstain the nuclei. Images were taken using a Zeiss LSM 880 Confocal Microscope (Zeiss, Oberkochen, Baden‐Württemberg, Germany).

### 2.6 Behavioral Tests

#### 2.6.1 Morris Water Maze Test

Spatial learning and memory were assessed using the Morris water maze (MWM) according to the established protocol. The MWM was performed in a circular tank (110 cm in diameter and 60 cm in height) and filled with white opaque water at approximately 22°C. Reference cues consisting of different colors and shapes were placed along the walls surrounding the tank. During the initial training stage, a platform (10 cm in diameter) was placed in a target quadrant, submerged 1 cm below the water surface. The mice were placed into the tank facing the side wall and near the edge at one of four points randomly. Mice were allowed to search for the platform for 60 s; if not found, they were gently guided to it. Animals were allowed to stay on the platform for 15 s. Four trials per day were conducted with a 1‐h intertrial interval. Between the trails, mice were gently padded dry and warmed on a heating pad. The training stage lasted for 5 days, recording the time needed to find the platform (escape latency) and swimming speed. The escape latency of mice that could not find the platform was recorded as 60 s. On the sixth day, the platform was removed, and the mice were placed in the pool from the opposite side of the platform. The time spent in target quadrants and the number of crossings of the area where the platform used to be recorded. Collected data were analyzed by Smart 3.0 Video Tracking Software (Panlab; Harvard Apparatus).

#### 2.6.2 Y‐Maze Test

Y‐maze testing was adapted to evaluate the short‐term spatial memory from the published protocols. The Y‐shape maze apparatus consisted of three light‐colored, opaque arms orientated at 120° angles from each other. The mouse was placed into the distal part of the labeled arm, facing toward the center of the maze. The mouse was allowed to explore the arms freely during an 8‐min period with no training, reward, or punishment. An entry occurred when the four paws of the mouse were completely within the arm. Consecutive entries into all three arms constituted an alternation. The number of all arm entries and alternation were recorded. The percentage of alternation was calculated using the following formula: %Alternation = (Number of Alternation/ [total number of arms entries−2]) × 100.

#### 2.6.3 Open‐Field Test

The open‐field test was adapted to assess locomotor activity, anxiety, and depression in mice. Mice were brought into the dim light behavior assessment room and were allowed to habituate for 30 min. Mice were gently put into the middle of a square box (40 cm by 40 cm by 40 cm) and allowed to freely explore the area for 10 min. The field was virtually divided into central and peripheral zones. During the analysis, the arena was divided into 25 zones (16 peripherals and 9 central). The spontaneous activity was digitally recorded and analyzed with Smart 3.0 video‐tracking software (Panlab, Harvard Apparatus). The distance traveled, mean velocity, and time spent in the center were documented as indicators of locomotor activity.

#### 2.6.4 Novel Object Recognition Test

The novel object recognition (NOR) test was performed to assess the mouse's ability to recognize a novel object in a familiar environment. After 24 h of habituation in the open‐field arena in the absence of objects, two identical objects in opposite corners were placed in the habitual arena, the mice were allowed to explore the two objects freely for 10 min. After an interval of 60 min, one of the objects was replaced with a novel one in its place, and the location of novel versus familiar object was alternated with each batch of test such that the number of times the familiar and novel object was placed at a particular corner was similar. The mice were allowed to explore the objects for 5 min and the interactions of mouse with the objects were video recorded. An interaction was defined by the nose of the mouse pointing to the object within a distance of 2 cm. Time spent exploring each object was quantified using the Smart 3.0 Video Tracking Software (Panlab; Harvard Apparatus). To determine the percent time with novel object, we calculate (time with novel object)/(time with trained object + time with novel object) × 100. Mice that did not explore both objects during the training phase were excluded from the analysis.

#### 2.6.5 Balance Beam Task

Balance and general motor function were assessed using the balance beam task. A 1‐cm dowel beam was attached to two support columns 44 cm above a padded surface. At the end of the 100 cm long beam, a 9 × 15 cm escape platform was attached. The animal was placed at the side of away from the escape platform of the beam and released. Each animal was given three trials during a single day of testing, with a 10‐min rest in their home cages between training sessions on the two beams. The following day, mice were placed on the side of away from the escape platform, and the escape latencies to enter the safe platform were recorded. The two types of beams were used in this test: the bar chart beam and the round beam. If an animal remained on the beam for entire 60‐s trial or escaped to the platform, the maximum time of 60 s was recorded.

#### 2.6.6 Clasping Test

The limb clasping test was used to quantify deficits in corticospinal function. To test clasping behavior, mice were suspended by the tail for 5–10 s, recording for at least 5 s. The score of limb clasping was assigned based on the following criteria:

0—No limb clasping, normal escape extension;

1—one hind limb exhibits complete splay and loss of mobility, toes exhibit normal splay;

2—Both hind limbs exhibit incomplete splay and loss of mobility, toes exhibit normal splay;

3—Both hind limbs exhibit clasping with curled toes and immobility;

4—Forelimbs and hind limbs exhibit clasping and are crossed, curled toes and immobility.

Suspension of mice by the tail elicits an escape response (“0”). Deficits in the ability to splay the hind limbs and extend the toes are scored based on their severity on a scale from 0 to 4.

#### 2.6.7 String Suspension Task

This motor performance test was performed essentially as described previously. In brief, the mice were permitted to grasp the string with their forepaws and then were released, recording the escape time before the mice fell. A rating system from 0 to 5 was used during the single 60‐s trial to assess each mouse's performance in this task:

0—unable to remain on the string;

1—hang only by fore or hind paws;

2—same as for 1 but attempts to climb onto string;

3—sits on string and is able to hold balance;

4—four paws and tail around string with lateral movement;

5—escape.

#### 2.6.8 Electroretinogram

Following overnight dark adaptation (> 12 h), animals were injected with a mixture of ketamine–xylazine. The eyes were then dilated using Mydrin‐P eye drops (Santen, higa, Japan) and cornea was kept hydrated using a lubricating gel (Lacryvisc gel, Alcon, Rueil‐Malmaison, France). All preparations were done under dim red light to maintain the dark‐adapted state of the animal. The visual electrophysiological measurement of electroretinogram (ERG) was conducted in each animal. During the recording period, the animal was placed on a warm platform to maintain the body temperature at around 37°C. For ERG recordings, needle electrodes inserted into the lateral canthi of each eye and base of tail served as reference and ground, respectively. A pair of gold ring electrodes (2 mm diameter) placed on the corneal surface of each eye served as active. An impedance of less than 5 KΩ for active electrode was maintained during the recording period for ERG. The ERG responses were measured using a full‐field Ganzfeld (Q450; RETI Animal, Roland Consult, Brandenburg an der Havel, Germany) with light‐emitting diode (LED) light source. Firstly, the scotopic ERG responses were measured using three flash intensities (0.01 and 3.0 cd.s/m^2^) and light‐adapted photopic flash responses (3.0 cd.s/m^2^ with background light of 30 cd/m^2^).

### 2.7 Tissue Preparation for Brain, Spinal Cord, and Retina

Brains were removed from the skull and dissected at the midline. Spinal cords were dissected out from different groups and removed from the skull. The eyes from different groups were removed from the socket. For biochemical analysis, half of the hemisphere was dissociated into different brain areas, including the frontal cortex and hippocampus. A small piece of the spinal cord close to cervical spine was cut, the left eye was dissociated into different eye areas including retina and optic nerve, and immediately snap‐frozen in the liquid nitrogen, and stored at −80°C before use.

For immunohistochemistry analysis, the other hemisphere of brain and spinal cords were fixed in 4% PFA overnight at 4°C, followed by transferring to 30% sucrose at 4°C for 48 h, before embedded in OCT. Brains were cut at a thickness of 25 μm, and sections were mounted on glass slides for future processing. Cross and horizontal sections of the spinal cord were cut at a thickness of 25 μm, and collected in wells containing 0.1% NaN3 in PBS buffer.

For immunohistochemistry analysis, the right eye was fixed in 4% PFA overnight at 4°C. Following fixation, the eyes were rinsed three times in PBS and processed for paraffin embedding with a tissue processor. Five‐micrometer paraffin sections were obtained and mounted on slides for immunohistochemistry.

### 2.8 Immunocytochemistry Staining of Brain, Spinal Cord, and Retina

Brain sections and free‐floating sections of the spinal cord were washed three times for 5 min with 0.5% Triton X‐100 in PBS (0.5% PBST) and incubated in 5% normal goat serum in 0.5% PBST for 1 h at room temperature, followed by incubation with primary antibodies overnight at 4°C. The following day, brain slices and spinal cord were incubated with the second antibodies for 2 h at room temperature. For nuclear staining, sections were incubated with DAPI (1:1000, Sigma‐Aldrich, St. Louis, MO, USA) for 5 min at room temperature. Sections were then washed, mounted, and sealed under coverslips.

The eyes sections were deparaffinized, followed by hot antigen retrieval. The samples were then washed three times for 5 min in PBS and incubated at room temperature with 5% normal goat serum in PBS containing 0.5% Triton X‐100 (0.5% PBST) for 1 h to block nonspecific binding. Following blocking, sections were incubated with the following primary antibodies overnight at 4°C. The following day, slices were incubated with secondary antibody for 2 h at room temperature. DAPI was utilized to counterstain the nuclei. Images were taken using a Zeiss LSM 800 confocal microscope (Zeiss, Oberkochen, Baden‐Württemberg, Germany).

For thioflavin‐S (Thio‐S) staining for brain and spinal cord slices, the sections were blocked by 5% normal goat serum in 0.5% PBST for 1 h, followed with 0.1 mg/mL Thio‐S for 8 min, washed with 70% alcohol twice, then with PBS three times. Slices were mounted with glass coverslips.

The following antibodies were used for immunocytochemistry detection in this study: anti‐Iba‐1 (Abcam, ab5076), anti‐Iba‐1 (Wako 019‐19741), anti‐LAMP‐1 (Abcam ab24170), anti‐PSD95 (Abcam ab18258), anti‐MAP2 (Abcam ab232454), anti‐synaptophysin (SYP, Abcam ab32127), anti‐4G8 (Biolegend, 800701), and anti‐NeuN (Abcam ab108319).

### 2.9 ELISA Analysis

For Aβ ELISA, the tissues from hippocampus, frontal cortex, spinal cord, and retina were homogenized in RIPA lysis buffer supplemented with protease inhibitor mix to obtain a soluble fraction for assessing cytokines and soluble Aβ. The resulting pellet containing insoluble amyloid‐β was extracted with 70% formic acid solution and neutralized with 1 M Tris–HCl, pH 11, for measuring insoluble Aβ. The levels of Aβ1‐42 were measured using sandwich ELISA techniques as described in the published protocols. The ELISA was developed using ELISA TMB. Synthetic human Aβ1‐42 peptide was used to generate the standard curves for each assay.

For cytokine levels in the frontal cortex and hippocampus, the ELISA kits (R & D systems, Minneapolis, MN, USA) were used to evaluate the expression of TNF‐α, IL‐1β, and IL‐6. The plates were coated with antibodies for TNF‐α, IL‐1β, and IL‐6 in coating buffer (0.5% PBST) overnight at room temperature. The plates were then blocked in blocking buffer for 2 h at room temperature, washed four times with washing buffer, and incubated with samples diluted 1:3 with blocking buffer for 2 h at room temperature. Plates were washed four times with washing buffer before incubation for 2 h at room temperature with the detection of biotinylated antibodies for TNF‐α, IL‐1β, and IL‐6. After the washing steps, the plates were incubated with Streptavidin HRP for 1 h in the dark. After four washing steps, the plates were treated with the TMB substrate, and the chromogenic reaction was monitored by detecting the absorbance at 450 nm using a Microplate reader EnSpire (PerkinElmer, Waltham, MA, USA).

### 2.10 Imaging Analysis

Immunostaining images were captured using a Zeiss LSM 800 confocal microscope (Zeiss, Oberkochen, Baden‐Württemberg, Germany). To measure Aβ deposition, Thio‐S positive areas were detected in frontal cortex, hippocampus, and spinal cord from a series of systematically selected brain sections. Images were captured with a 20× objective lens and two‐fourth sections were taken for each mouse brain. The number and area of Aβ deposits were quantified using ImageJ (NIH, Bethesda, MD, USA) software.

To quantify microglia in the brain and spinal cord, images were collected in a Z series of 20–30 μm depth with 2‐ to 3‐μm intervals between images. Ten to fifteen pictures of 20–30 μm thickness were captured from 3 to 4 sections for each mouse brain, starting from the specific areas of dentate gyrus (DG), CA1, cortex, and spinal cord. The maximum pictures were obtained with the Zeiss software (Zeiss, Oberkochen, Baden‐Württemberg, Germany) and MetaMorph 7.8.0.0 (MetaMorph Inc. Tennessee, USA), and ImageJ (NIH, Bethesda, MD, USA) was used to analyze the number, lengths, and the soma of microglia.

To quantify microglial activation in the retinal sections, image was captured from the central (100–300 μm from the optic nerve), mid‐central (300–500 μm from the optic nerve), and peripheral areas (beyond 500 μm from the optic nerve) of the retinal sections. In the analysis, we defined ROI that included all retinal layers (including nerve fiber layer, ganglion cell layer, inner plexiform layer, inner nuclear layer, outer plexiform layer, outer nuclear layer) within each image. ImageJ (NIH, Bethesda, MD, USA) was used to analyze the fluorescence intensity and Iba‐1‐positive area of retinal microglia.

To assess the correlation between LAMP‐1 and Iba‐1 using Pearson's coefficient, start by opening the images in ImageJ software (NIH, Bethesda, MD, USA). Separate the two channels featuring the target proteins into distinct black‐and‐white images. If the images are in 16‐bit, convert them to 8‐bit for easier handling. Optionally, pseudo‐colors can be added to facilitate distinction between the channels. Subsequently, a specific ROI should be selected to minimize background interference. Then, open the Coloc two plugin in ImageJ, choose the two relevant channels, and enable all algorithm options. Adjust the Threshold setting as necessary. Click “OK” to initiate the analysis. Upon completion, a report containing results such as Pearson's correlation coefficient will be generated. The results can then be saved as a PDF or exported as data for further analysis.

To quantify presynapse and postsynapse, images were captured with a 40× objective lens and collected in a Z series of 20 μm depth with 2‐μm intervals between images. The maximum pictures were obtained using the Zen software, and the relative density and dot points of PSD95 and SYP were quantified using ImageJ (NIH, Bethesda, MD, USA) software.

### 2.11 Western Blot

Briefly, tissues from hippocampus, frontal cortex, spinal cord, and retina were extracted using RIPA lysis buffer supplemented with a protease inhibitor mix, and centrifuged at 80,000 for 30 min. The supernatant was collected, and protein concentration was analyzed using the BCA protein assay kit (Thermo Fisher Scientific, 23225) according to the manufacturer's instruction. Equal amounts of protein (25 μg/lane) were resolved on an SDS‐polyacrylamide gel and transferred onto a PVDF membrane. The membrane was blocked with fresh blocking buffer containing 5% skim milk powder at room temperature for 1 h, followed by incubation with primary antibodies at 4°C overnight. The following day, the membranes were incubated with the horseradish peroxidase (HRP)‐conjugated secondary antibody (1:3000) for 2 h at room temperature. Finally, protein band intensities were detected by the chemiluminescent imaging system (Bio‐Rad, Hercules, CA, USA). The gray value of each band was analyzed by ImageJ software (NIH, Bethesda, MD, USA).

The following antibodies were used: anti‐synaptophysin (SYP, Abcam ab32127), anti‐4G8 (Biolegend, 800,701), anti‐p‐Akt(s473, CST, #4046 s), anti‐AKT(CST, #2920 s), anti‐p‐44/42(T202, CST, #4370 s), anti‐44/42 Erk1/2(CST, #4695 s), anti‐p‐CREB(s133, CST, #9198 s), anti‐CREB (CST, #9104), anti‐GAPDH(CST, #5174P), anti‐inducible nitric oxide synthase (iNOS) (CST, # 13120), anti‐Arg‐1 (CST, # 93668), anti‐IL‐1β (CST, #12242), anti‐TNF‐α (CST, # 6954), and anti‐IL‐6 (CST, # 12912).

### 2.12 Statistical Analysis

Statistical analysis was performed using Prism 9.0 software (GraphPad software, San Diego, California, USA). Comparison among multiple groups was conducted using one‐way ANOVA by Tukey's multiple comparison test, or by Kruskal–Wallis test followed by Dunn's multiple comparison test when the data did not meet a Gaussian distribution. Comparisons between two groups were performed using an unpaired Student's *t*‐test, or by Mann–Whitney test when the data did not meet a Gaussian distribution. For the MWM test, two‐way ANOVA followed by Tukey's multiple comparison test was applied for statistical analysis. Data were shown as mean ± SEM. Significant differences were considered when *p* < 0.05.

## 3 Results

### 3.1 LBE Improved Cognitive Function, Motor Function, and Retinal Response in 5xFAD Mice

**FIGURE 1:**
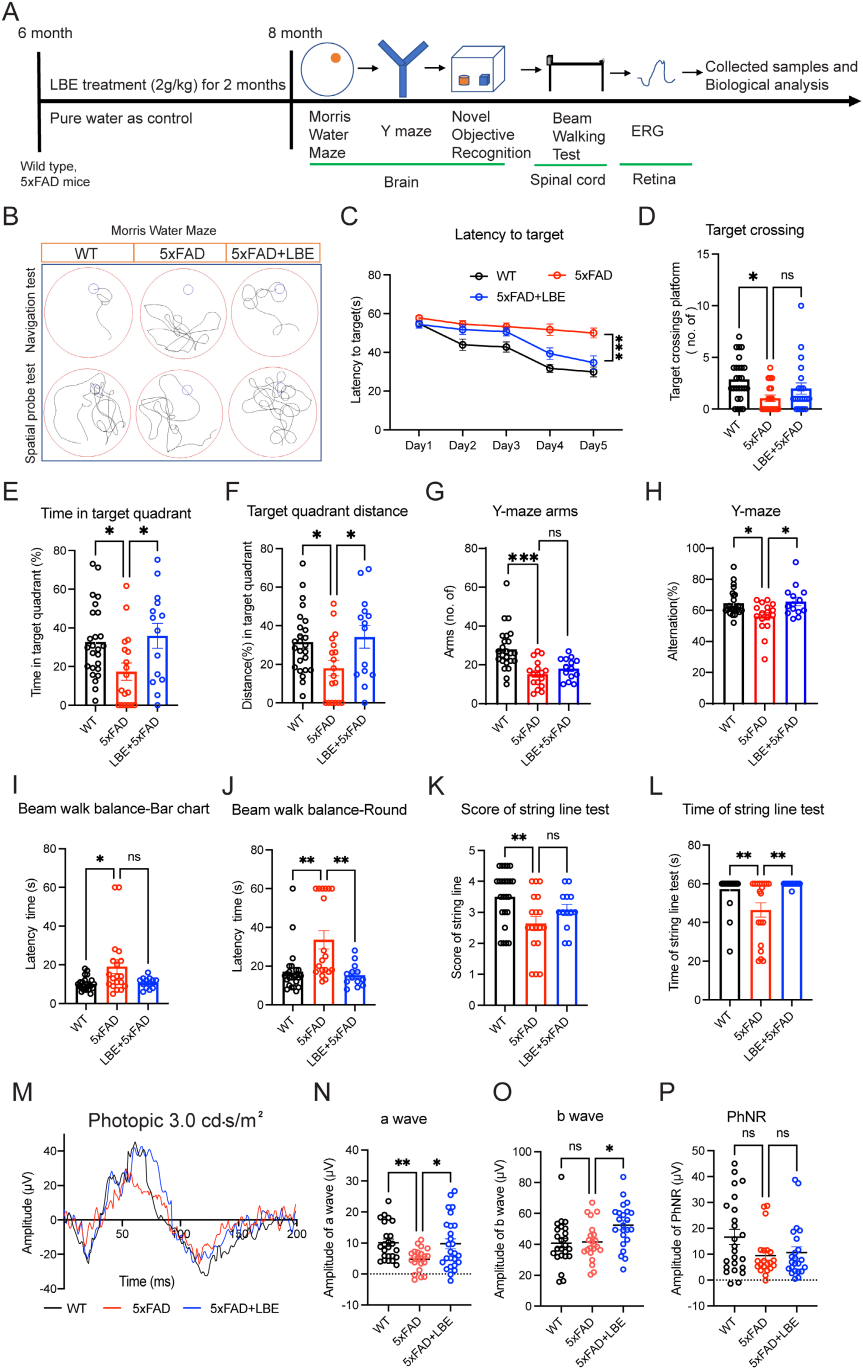
LBE promoted cognitive memory, motor movement, and retinal responses in 5xFAD mice. (A) Timeline of experiments design of LBE treatment in 5xFAD mice. (B) Representative pictures of navigation and spatial probe tests in 5xFAD mice using the MWM. (C) Latency for the mice to reach the platform during the first 5 days of trials (*n* = 25 WT, *n* = 19 water treatment, *n* = 21 LBE treatment, **p < 0.01, two‐way ANOVA with Tukey's multiple comparison test). (D) Number of platform crossings during probe trail (*n* = 25 WT group, *n* = 19 5xFAD group, *n* = 21 5xFAD + LBE group, Kruskal–Wallis test with Dunn's multiple comparison test). (E) Time spent in the target quadrant during the probe trial. (F) Distances covered in the target quadrant during the probe trial. (G) Total arm entries in Y‐maze task were comparable among groups. (H) Spontaneous alternation in Y‐maze task of 5xFAD mice (*n* = 24 WT group, *n* = 17 5xFAD group, *n* = 14 5xFAD + LBE group, Kruskal–Wallis test with Dunn's multiple comparison test). (I) Qualification of the latency time in the beam balance walking test (bar chart). The latency time of 5xFAD mice significantly decreased compared to WT mice. (J) Qualification of the latency time in the beam balance walking test(round). (K) Qualification of the score in string suspension task among WT, 5xFAD, and 5xFAD + LBE mice. (L) Qualification of the time in the string suspension task. (M) Representative ERG waveform to a photopic 3.0 cd.s/m^2^ flash under light adaptation of WT group (dark), 5xFAD (red), and 5xFAD + LBE (blue). (N, O) Scattered plots of the amplitude of a‐wave and b‐wave in the ERG of different groups under light adaptation and flash in 3.0 cd.s/m^2^. (P) Scattered plot of the amplitude of photopic‐PhNR in the different groups under light adaptation and flash in 3.0 cd.s/m^2^. Data are shown as mean ± SEM. *p < 0.05; **p < 0.01; ***p < 0.001 by one‐way ANOVA test with Dunn's multiple comparison test. WT, the littermate of 5xFAD that does not carry the mutations; 5xFAD mice treated with water; 5xFAD mice treated with LBE at the dose of 2 g/kg body weight. Numbers within the bars indicate the number of mice recorded for each animal group.

Previous studies have established the 5xFAD mouse model as a reliable simulation of AD pathology, characterized by the presence of amyloid plaques, glial activation, and cognitive impairment [9, 26]. To evaluate the therapeutic potential of LBE for AD treatment, we initiated daily intragastric gavage with LBE in 6‐month‐old 5xFAD mice and assessed its therapeutic effect at 8 months (Figure 1A). After 2 months of LBE administration, from 6 to 8th month of age, we evaluated the mouse's cognitive, motor, and visual functions through various behavioral tests.

We first assessed spatial learning and memory function using the MWM test. As expected, untreated 5xFAD mice showed deficits in finding the hidden platform compared to wild‐type (WT) mice (Figure 1B,C). However, LBE‐treated 5xFAD mice exhibited a significant reduction in latency to reach the platform during training days, indicating improved spatial learning (***p < 0.001; Figure 1C). During the spatial‐probe test, LBE‐treated mice spent more time in the target quadrant (35.91% ± 6.39% vs. 16.47 ± 4.29, *p = 0.0216; Figure 1E), as well as covered more distance in target quadrant (*p = 0.0376 vs. water‐feeding group; Figure 1F), further confirming their improved memory retention. The total arms showed an increased trend after LBE treatment in the Figure 1G. LBE‐treated 5xFAD mice exhibited a significantly higher alternation rate compared to untreated 5xFAD mice (65.80% ± 2.60% vs. 56.18% ± 2.39%, *p = 0.0346; Figure 1H), approaching levels similar to WT mice. This suggested that LBE treatment improved cognition in 5xFAD mice. The open‐field test revealed no significant difference in center time between LBE‐fed and untreated 5xFAD mice (16.13% ± 1.90% vs. 15.52% ± 1.90%, *p* = 0.1609; Figure S1E), but LBE‐fed mice traveled faster, indicating preserved locomotor activity (Figure S1G). In the NOR test, the time exploring two objects and the recognition index did not reveal significant improvements in object recognition among all testing groups (Figure S1H–J).

Then, the beam balance test was used to assess motor balance and coordination. The mice were trained to traverse the elevated and narrow beams to reach an enclosed escape platform, and the escape latency was measured. The LBE treatment group spent less time traveling on the bar‐chart beam (10.69 ± 0.79 vs. 19.16 ± 3.64, *p* = 0.4458; Figure 1I) and the round beam (15.38 ± 1.63 vs. 33.63 ± 4.70, **p = 0.0085; Figure 1J) to the escape platform compared to the control group. The limb clasping test evaluated the deficits in corticospinal function. The limbs exhibited clasping with curled toes in the 5xFAD mice at the position indicated by the white arrow (Figure S2A). The score of 5xFAD mice significantly increased compared to WT mice (1.92 ± 0.21 vs. 0.75 ± 0.10, ****p < 0.0001; Figure S2B), while the LBE feeding did not affect the clasping test score. Another behavioral test, the string suspension task, allowed the mice to grasp the string with their forepaws and then measured the escape time before falling. Compared to WT mice, 5xFAD mice exhibited a significantly lower score (3.50 ± 0.18 vs. 2.64 ± 0.23, **p = 0.0066; Figure 1K), and reduced escape time (57.29 ± 1.67 vs. 46.47 ± 3.72, **p = 0.0026; Figure 1L). LBE feeding significantly enabled the 5xFAD mice to stay longer on string suspension task, improving from 46.47 ± 3.72 to 59.69 ± 0.31 (**p = 0.0037; Figure 1L).

Visual function was assessed using the ERG test. LBE significantly increased the amplitude of the a‐wave (22.61 ± 3.45 μV vs. 7.75 ± 2.01 μV, *p = 0.00420; Figure S2C–E), while no significant difference in b‐wave amplitude was observed compared to the water‐fed group at low flash intensity (0.01 cd.s/m^2^). The response was similar between LBE‐treated group and the water‐fed group at high flash intensity (3.0 cd.s/m^2^) (Figure S2F–H). Photopic ERG was measured after 5 min of light adaption. The average amplitude of a‐wave in LBE‐treated group was larger than in the water‐fed group (11.06 ± 1.63 μV vs. 4.49 ± 0.74 μV, *p = 0.0128; Figure 1M), and the b‐wave was significantly higher (52.35 ± 2.95 μV vs. 41.49 ± 2.76 μV, *p = 0.0301; Figure 1N) in photopic ERG. No significant difference between the LBE‐treated group and water‐fed group was evident at the 3.0 cd.s/m^2^ for the photopic‐PhNR amplitude (Figure 1P). These results indicated that LBE treatment in 5xFAD mice leads to improvements in cognitive function, motor performance, and retinal response.

### 3.2 LBE Treatment Reduced Amyloid‐β Load in CNS of 5xFAD Mice

**FIGURE 2:**
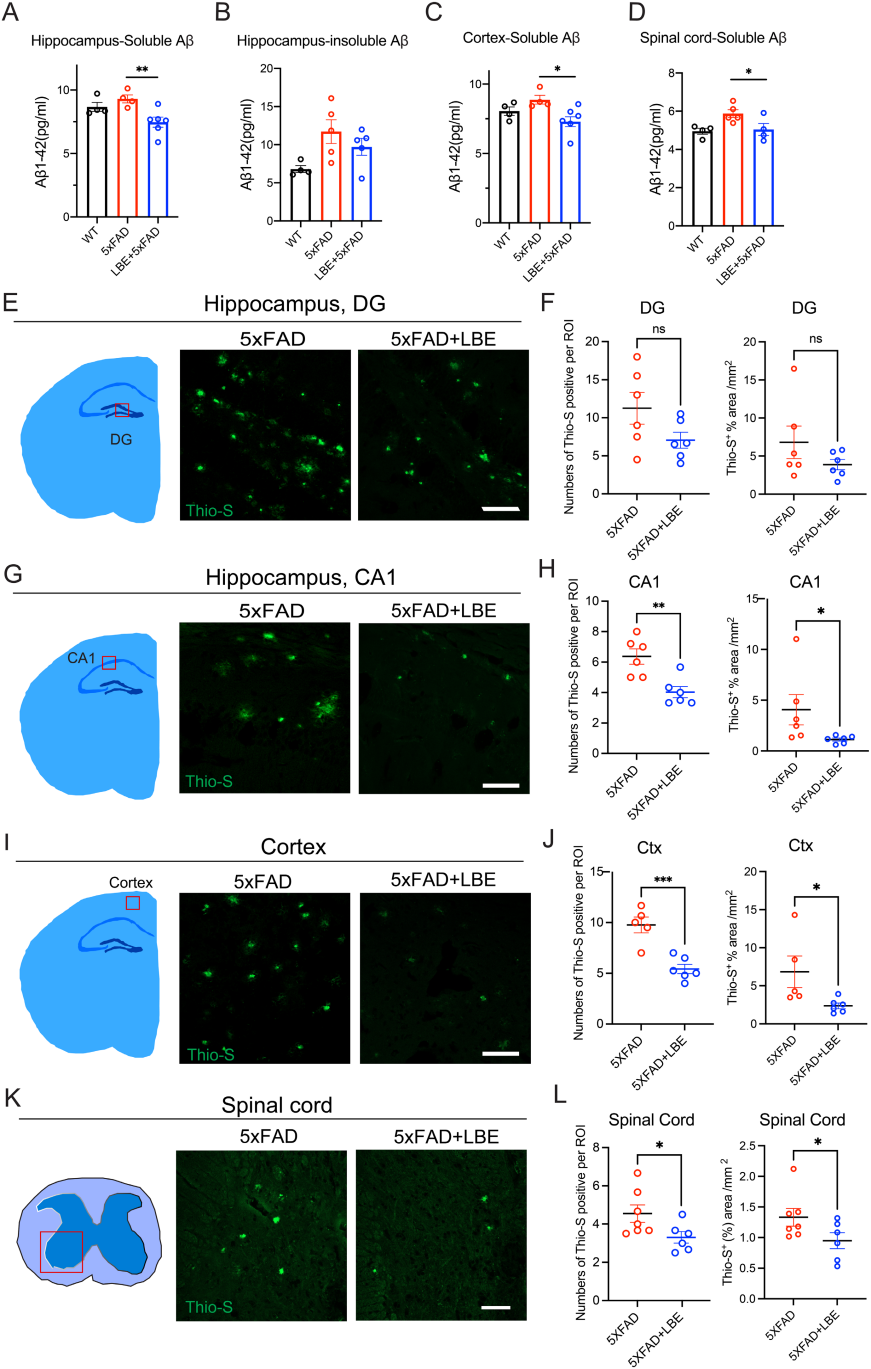
LBE treatment reduced Aβ load in the brain and spinal cord of 5xFAD mice. (A) Qualification protein level of soluble Aβ1‐42 in the hippocampus using ELISA. (B) Qualification protein level of insoluble Aβ1‐42 in the hippocampus using ELISA. (C) Qualification of protein level of soluble Aβ1‐42 in the cortex using ELISA. (D) Qualification of protein level of soluble Aβ1‐42 in the spinal cord using ELISA. (E) Schematic diagram of selected fields of view in the hippocampus and images of DG region of the hippocampus slices stained for Thio‐S, labeling Aβ plaques in 5xFAD and 5xFAD + LBE mice. Scale bar: 50 μm. (F) Qualification of the number and area of Thio‐S‐positive staining in DG. (G) Schematic diagram of selected fields of view in the hippocampus and images of CA1 of the hippocampus slices stained for Thio‐S, labeling Aβ plaques in 5xFAD and 5xFAD + LBE mice. Scale bar: 50 μm. (H) Qualification of the number and area of Thio‐S‐positive staining in CA1. (I) Schematic diagram of selected fields of view in the cortex and images of cortex slices stained for Thio‐S, labeling Aβ plaques in 5xFAD and 5xFAD + LBE mice. Scale bar: 50 μm. (J) Qualification of the number and area of Thio‐S‐positive staining in the cortex. (K) Schematic diagram of selected fields of view in the spinal cord and images of spinal cord slices stained for Thio‐S, labeling Aβ plaques, and merged images with DAPI in 5xFAD and 5xFAD + LBE mice. Scale bar: 50 μm. (L) Qualification of the number and area of Thio‐S‐positive staining in the spinal cord. Data are shown as mean ± SEM. *p < 0.05; **p < 0.01; ***p < 0.001 by one‐way ANOVA test with Dunn's multiple comparison test and unpaired two‐tailed Student's *t*‐test with two groups.

To assess the effect of LBE treatment on AD‐related pathology, we examined the amyloid‐plaque load in the CNS, including the hippocampus, cortex and spinal cord, using the ELISA test and Thio‐S staining. Soluble Aβ1‐42 levels in the LBE group were significantly reduced in the hippocampus compared to 5xFAD mice, and there was a downward trend in insoluble Aβ1‐42 in the hippocampus (Figure 2A,B). Similar results were observed in the cortex and spinal cord, where the expression of Aβ load was dramatically reduced after LBE treatment (Figure 2C,D). In the DG region, no significant differences were detected in the number and area of Thio‐S‐positive staining (Figure 2E,F and Figure S3A). In the CA1 region, LBE treatment significantly decreased the Aβ load in both number (4.03 ± 0.37 vs. 6.36 ± 0.50, **p = 0.0038; Figure 2G,H) and area (1.13% ± 0.15% vs. 4.06% ± 1.49%, *p = 0.0152; Figure 2G,H). In the cortex, compared to the water‐fed group, LBE treatment significantly reduced the number (5.43 ± 0.46 vs. 9.77 ± 0.78, ***p < 0.001; Figure 2I,J) and area (2.37 ± 0.37 vs. 6.83 ± 2.07, *p = 0.0451; Figure 2I,J) of plaques. In the spinal cord, Thio‐S‐positive Aβ plaques were primarily observed in the ventral horn (Figure 2K and Figure S3B). LBE feeding significantly reduced the number from 4.55 ± 0.46 to 3.30 ± 0.30 (*p = 0.0256; Figure 2L) and the area of Aβ plaques from 1.33 ± 0.14 to 0.95 ± 0.13 (*p = 0.0393; Figure 2L).

Next, immunoblotting was used to further evaluate the Aβ load. LBE treatment significantly reduced Aβ load in the hippocampus with the two specific Aβ antibodies, 4G8 (*p = 0.0193 vs. water‐fed group; Figure S4A) and Aβ1‐42 (**p = 0.0017 vs. water‐fed group; Figure S4B). Similar results were observed in the cortex, where the expression of Aβ load was dramatically reduced after LBE treatment (*p = 0.0032 vs. water‐feeding group; Figure S4C). In the spinal cord, LBE treatment reduced the expression of 4G8 from 1.01 ± 0.08 to 0.65 ± 0.10 (*p = 0.0385; Figure S4D) and decreased the level of the Aβ1‐42 from 1.28 ± 0.065 to 0.83 ± 0.15 (*p = 0.0305; Figure S4E). Additionally, LBE treatment significantly reduced the expression of Aβ load (*p = 0.0136; Figure S4F) in the retina. In summary, these results revealed that LBE treatment significantly reduced the Aβ load in the CNS of 5xFAD mice.

### 3.3 LBE Modified the Morphological Feature of Microglia in the CNS (Brain, Spinal Cord, and Retina) of 5xFAD Mice

**FIGURE 3:**
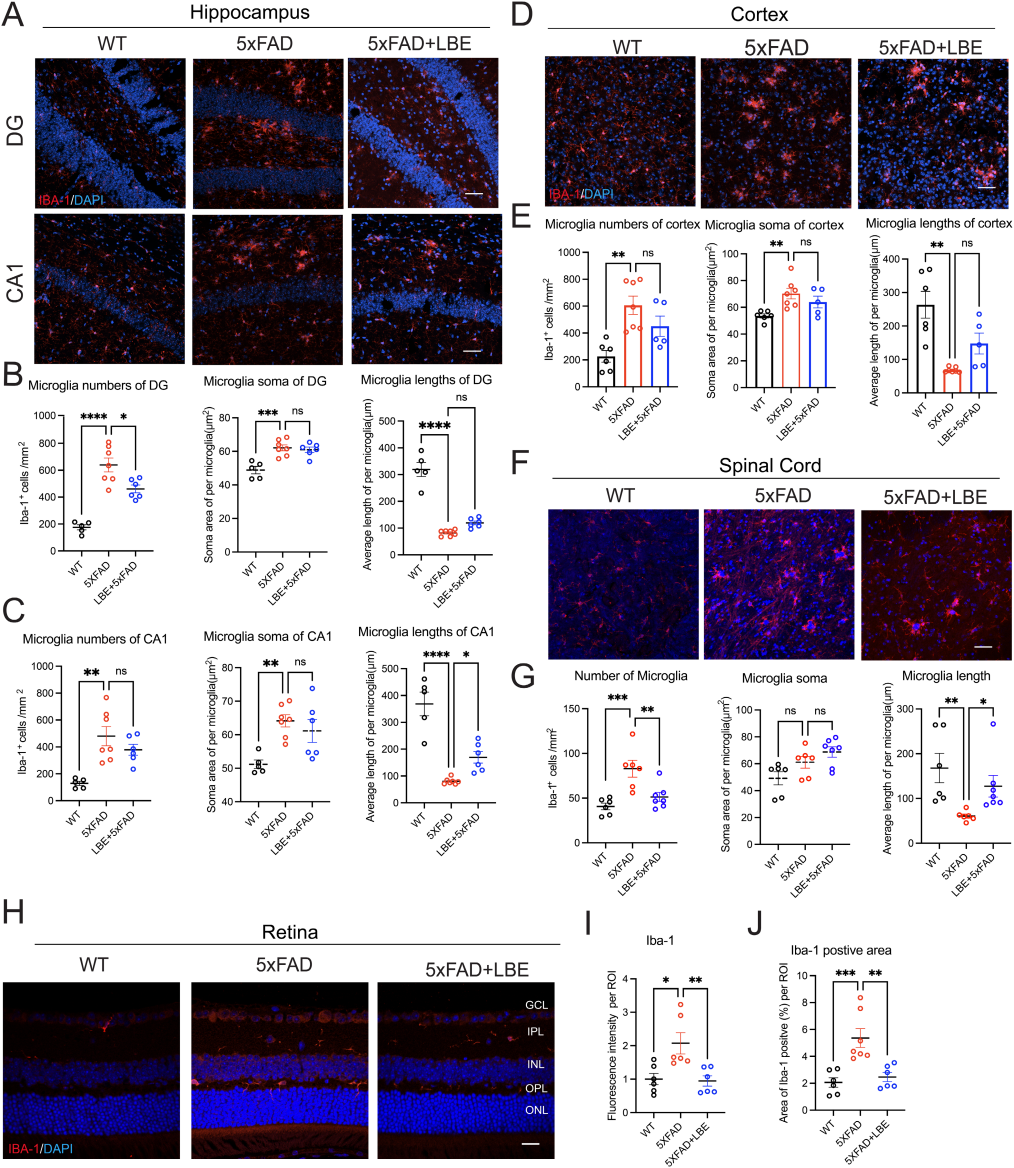
LBE treatment modified the morphological feature of microglia in the CNS (brain, spinal cord, and retina) of 5xFAD mice. (A) Images of brain slices stained for Iba‐1 antibody (red) from WT, 5xFAD and 5xFAD + LBE mice. Brain regions including DG and CA1 in the hippocampus were showed in the different rows. (B) Quantification of the number, soma size, and average length of Iba‐1‐positive microglia in the DG area. (C) Quantification of the number, soma size, and average length of Iba‐1‐positive microglia in the CA1 area. (D) Images of brain slices stained for Iba‐1 antibody (red) labeling microglia in the cortex from WT, 5xFAD and 5xFAD + LBE mice. Scale bar: 50 μm. (E) Quantification of the average length, number, and soma size of Iba‐1‐positive microglia in the cortex. (F) Images of spinal cord slices stained for Iba‐1 antibody (red) from WT, 5xFAD, and 5xFAD + LBE mice. (G) Quantification of the number, soma size, and average length of Iba‐1‐positive microglia in the spinal cord. Scale bar: 50 μm. (H) Representative images of Iba‐1‐positive microglia in the retina of 5xFAD mice. (I) Quantification of the mean fluorescence intensity of Iba‐1‐positive microglia in the retina. (J) Quantification of the area of Iba‐1‐positive microglia in the retina. Scale bar: 20 μm. Data are shown as mean ± SEM. *p < 0.05; **p < 0.01; ***p < 0.001 by one‐way ANOVA test with Dunn's multiple comparison test, and unpaired two‐tailed Student's *t*‐test with two groups.

Our previous study demonstrated that LBE attenuated the oligomeric Aβ‐induced neuroinflammatory response of microglia by suppressing the release of inflammatory factors and promoting the release of anti‐inflammatory factors [22]. After confirming the reduction of Aβ load in the brain, we examined whether the effects of LBE on the amyloid‐related pathology were mediated through the regulation of neuroinflammation. Firstly, we performed immunostaining for the Iba‐1‐positive microglia in the hippocampus. The microglial morphology in 5xFAD mice showed enlargement of soma, thickening, and an increased number and average length in DG and CA1 regions compared to WT mice (Figure 3A). LBE induced minor changes in the microglial morphology of the hippocampus. Compared to water‐fed group, LBE significantly decreased the number of microglia in the DG region (461.2 ± 29.11 vs. 624.1 ± 58.70, *p = 0.0269; Figure 3B). Less thickening and longer average length were observed in the LBE group in the CA1 region (169.5 ± 21.59 vs. 80.36 ± 4.29, *p = 0.0427; Figure 3C). Although the average length of the microglia exhibited an upward trend in the DG region, there was no statistical difference (Figure 3B). Next, we tested the immunostaining for the Iba‐1‐positive microglia in the cortex (Figure 3D). Compared to WT mice, 5xFAD showed a significant increase in the number (**p = 0.0014; Figure 3E), and soma size (**p = 0.0089; Figure 3E) of microglia, as well as a remarkable reduction in the average length of microglia (**p = 0.0015; Figure 3E). Although LBE treatment reversed these changes, with less thickening and longer average length (147.6.00% ± 31.33% vs. 68.31% ± 2.89%, *p* = 0.0848; Figure 3E), there is no statistical difference.

Similar to the microglial changes observed in the brain of 5xFAD mice, there was a significant increase in the number of microglia in the spinal cord compared to WT mice (82.91 ± 9.42 vs. 40.73 ± 3.71, ***p < 0.001; Figure 3F,G). Although no significant difference was found in the soma size of microglia, the average length of the microglia was notably reduced in 5xFAD mice compared to WT mice (60.71 ± 24.11 vs. 168.0 ± 32.56, **p = 0.0031; Figure 3G). LBE feeding considerably reduced the number of microglia from 82.91 ± 9.42 to 51.37 ± 5.04 (**p = 0.0075) and increased the average lengths from 60.71 ± 24.11 to 127.4 ± 24.11 (*p = 0.0199; Figure 3G). In AD, reactive microglia were also observed in the retina of 5xFAD mice. The fluorescence intensity in the LBE‐fed group significantly decreased compared to the 5xFAD mice, and the positive area in 5xFAD was much larger than that in the LBE‐fed and WT mice (Figure 3H–J). Collectively, these results suggested that LBE may decrease the microglial activation, thereby influencing amyloid‐related pathology.

### 3.4 LBE Inhibited the Pro‐Inflammatory Response and Promoted the Anti‐Inflammatory Expression in the CNS (Brain, Spinal Cord, and Retina) of 5xFAD Mice

**FIGURE 4:**
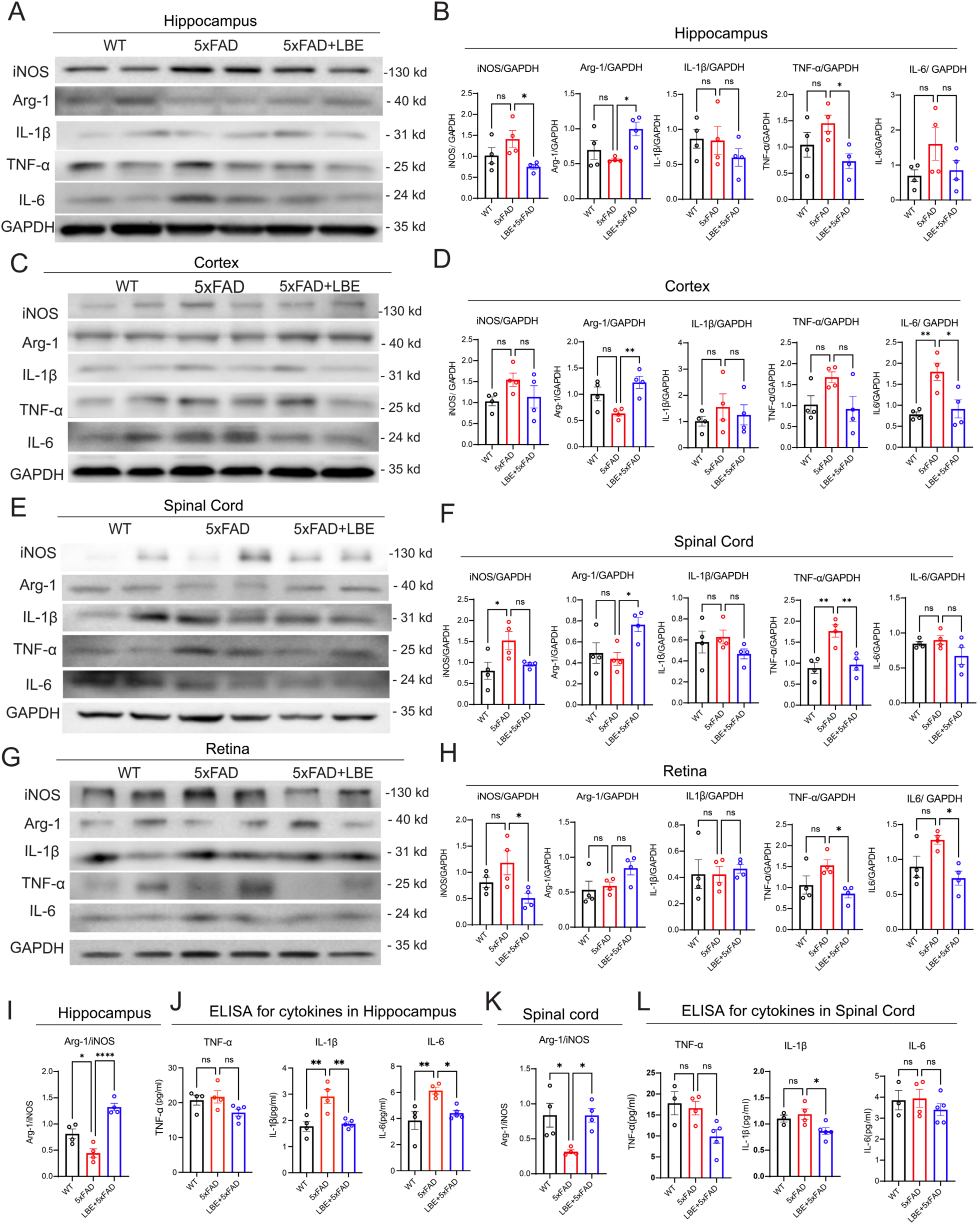
LBE reduced the pro‐inflammatory cytokines and increased the expression of anti‐inflammatory‐Arg‐1 in the CNS of 5xFAD mice. (A) Representative images of antibodies for pro‐inflammatory factors and anti‐inflammatory protein in the hippocampus after LBE treatment. (B) Qualification of protein levels of iNOS, Arg‐1, IL‐1β, TNF‐α, and IL‐6 in the hippocampus. (C) Representative images of antibodies for pro‐inflammatory factors and anti‐inflammatory protein in the cortex after LBE treatment. (D) Qualification of protein levels of iNOS, Arg‐1, IL‐1β, TNF‐α, and IL‐6 in the cortex. (E) Representative images of antibodies for pro‐inflammatory factors and anti‐inflammatory protein in the spinal cord after LBE treatment. (F) Qualification of protein levels of iNOS, Arg‐1, IL‐1β, TNF‐α, and IL‐6 in the spinal cord. (G) Representative images of antibodies for pro‐inflammatory factors and anti‐inflammatory protein in the retina after LBE treatment. (H) Qualification of protein levels of iNOS, Arg‐1, IL‐1β, TNF‐α, and IL‐6 in the retina. (I) Qualification of the ratio of Arg‐1/iNOS in the hippocampus. (J) Qualification of protein levels of TNF‐α, IL‐1β, and IL‐6 in the hippocampus using ELISA. (K) Qualification of the ratio of Arg‐1/iNOS in the spinal cord. (L) Qualification of protein levels of TNF‐α, IL‐1β, and IL‐6 in the spinal cord with ELISA. Data are shown as mean ± SEM. *p < 0.05; ***p < 0.001 by one‐way ANOVA test with Dunn's multiple comparison test, and unpaired two‐tailed Student's *t*‐test with two groups.

LBE induced minor changes in the microglial morphology within the CNS and partially reversed the microglia state to a protective phenotype. Immunoblotting was used to further evaluate the neuroinflammatory response in the brain, spinal cord, and retina (Figure 4). Compared to water‐fed group, LBE treatment significantly increased the expression of Arg‐1 in the hippocampus (1.00 ± 0.10 vs. 0.56 ± 0.02, *p = 0.026; Figure 4B) and cortex (1.18 ± 0.12 vs. 0.62 ± 0.04, **p = 0.0098; Figure 4D), and significantly decreased iNOS expression (0.75 ± 0.056 vs. 1.41 ± 0.20, *p = 0.0421; Figure 4B) in the hippocampus. LBE also significantly decreased the pro‐inflammatory cytokines TNF‐α in the hippocampus and IL‐6 in the cortex (Figure 4B,D).

Consistent with the findings in the brain, the expression of Arg‐1 in LBE treatment was increased (*p = 0.0373; Figure 4E) in the spinal cord, while the iNOS expression and pro‐inflammatory cytokine TNF‐α was deceased (Figure 4F). In the retina, LBE treatment significantly decreased iNOS expression and the pro‐inflammatory cytokine TNF‐α compared to 5xFAD mice (Figure 4G,H). Additionally, the ratio of Arg‐1/iNOS significantly increased both in the hippocampus (****p < 0.0001; Figure 4I) and the spinal cord (*p = 0.0477; Figure 4K), suggesting increased Arg‐1^+^ microglial activation. The ELISA test showed that LBE significantly decreased the levels of the pro‐inflammatory cytokines with IL‐1β and IL‐6 in the hippocampus (Figure 4J), and TNF‐α and IL‐1β in the spinal cord (Figure 4L). The downregulation of pro‐inflammatory markers and upregulation of anti‐inflammatory markers in the brain, spinal cord, and retina suggested that LBE switched the microglial phenotype in the CNS (brain, spinal cord, and retina) from pro‐inflammatory(iNOS^+^) to neuroprotective (Arg‐1^+^), which may contribute to delaying AD pathology.

### 3.5 LBE Promote Microglia Phagocytosis and Clearance of Aβ

**FIGURE 5:**
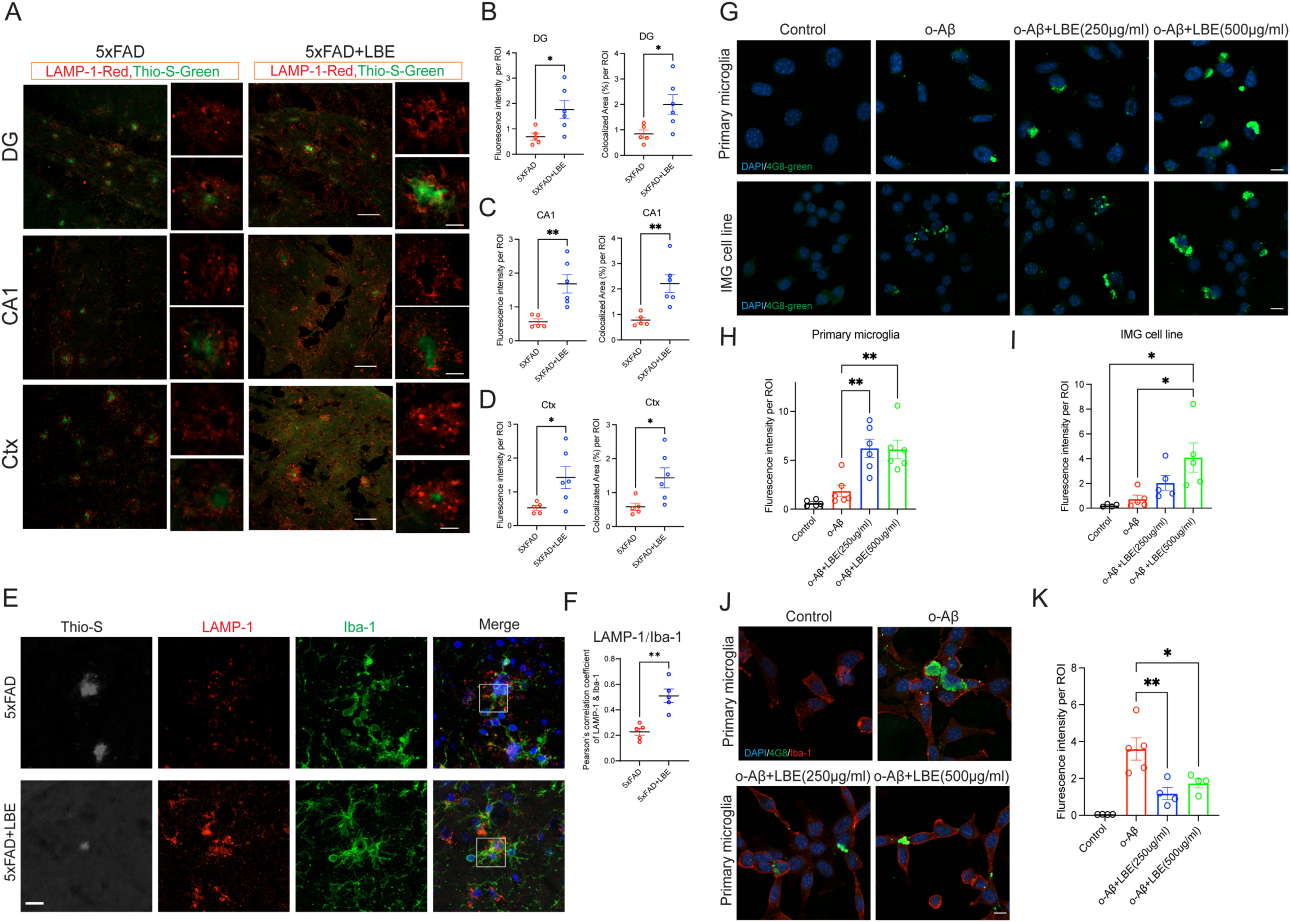
LBE promoted o‐Aβ uptake and degradation in the brain of 5xFAD mice and culture microglial cells. (A) Images of brain slices stained for Thio‐S (green) labeling Aβ plaques and LAMP‐1 (red) labeling lysosome from 5xFAD and 5xFAD + LBE mice. Brain regions including DG and CA1 of the hippocampus are shown in the different rows. Scale bar: 50 μm, large scale bar: 10 μm. (B) Quantification of the fluorescence intensity and colocalized area of LAMP‐1 positive in the DG region. (C) Quantification of the fluorescence intensity and colocalized area of LAMP‐1 positive in the CA1 region. (D) Quantification of the fluorescence intensity and colocalized area of LAMP‐1 positive in the cortex region. *n* = 5 5xFAD group, *n* = 6 5xFAD + LBE group, unpaired Student's *t*‐test. ns, not significant. *, *p* < 0.05; ***, *p* < 0.001. (E) Representative images of brain slices with Thio‐S labeled Aβ plaques and LAMP‐1 labeled lysosome, as well as Iba‐1 for microglia. Scale bar: 10 μm. (F) Colocalization analysis using Pearson's correlation coefficient with LAMP‐1 and Iba‐1. (G) Primary microglia and IMG cells were pretreated with different doses of LBE for 1 h, then coincubated with o‐Aβ for another hour. Cells were then collected and examined for intracellular Aβ using the specific 4G8 antibody with immunofluorescence staining. Scale bar: 10 μm. (H) Quantification of the fluorescence intensity of 4G8‐positive signal labeling intracellular o‐Aβ in primary microglia. (I) Quantification of fluorescence intensity of 4G8‐positive signal labeled with intracellular o‐Aβ in IMG microglial cell line. (J) Primary microglia were pretreated with different doses of LBE for 1 h, then coincubated with o‐Aβ for 23 h. Intracellular Aβ was then examined using the specific 4G8 antibody with immunofluorescence staining. (K) Quantification of the fluorescence intensity of 4G8‐positive signal labeled intracellular o‐Aβ in primary microglia. Scale bar: 10 μm. Data are presented as mean ± SEM. *, *p* < 0.05; **, *p* < 0.01, significant differences were determined using one‐way ANOVA followed by Tukey's multiple comparison test.

Given the increase in Arg‐1^+^ activated microglia and the reduction of amyloid load following LBE treatment, we investigated whether the Arg‐1‐positive activated microglia enhanced phagocytosis to clear Aβ. Microglia clustering around the plaques suggests an attempt to remove Aβ plaques through phagocytosis and degradation. Compared to the water‐fed group, LBE significantly increased the fluorescence intensity of LAMP‐1 in the DG (*p = 0.0290; Figure 5A,B), CA1(**p = 0.0055; Figure 5A,C), and cortex (*p = 0.0374; Figure 5A,D). Quantitative assessment of the colocalized areas of Aβ plaques (Thio‐S positive) and LAMP‐1 showed significant increases in the DG (*p = 0.0308; Figure 5B), CA1 (**p = 0.0060; Figure 5C), and the cortex (*p = 0.0336; Figure 5D). Additionally, LAMP‐1 immunoreactivity was observed surrounding Aβ plaques and colocalized with Iba‐1‐positive microglia (Figure 5E). Colocalization analysis using Pearson's correlation coefficient with LAMP‐1 and Iba‐1 showed a noticeable increase in LBE‐treated when compared to water‐fed group (Figure 5F).

We next investigated the effects of LBE on the uptake and degradation of Aβ in culture microglia. Primary microglia and IMG microglial cells were pretreated with 250 μg/mL and 500 μg/mL for 1 h, followed by coincubation with o‐Aβfor another hour. Cells were then collected and examined for intracellular Aβ using specific 4G8 antibody with immunofluorescence staining (Figure 5G). The fluorescence intensity significantly increased in both primary microglia (**p = 0.0030; Figure 5H) and IMG cells (*p = 0.0197; Figure 5I) in the LBE‐treated group compared to the o‐Aβ alone group, suggesting that LBE enhanced the o‐Aβ uptake. To examine the role of LBE in microglia‐mediated endocytic clearance, primary microglia were incubated with o‐Aβ, o‐Aβ + 250 μg/mL LBE, o‐Aβ + LBE (500 μg/mL) for 24 h, with nontreatment primary microglia served as the control group. Fluorescence signals of 4G8 were found inside the cells (Figure 5J). Both doses of LBE treatment significantly reduced 4G8‐positive expression in the primary microglia (**p = 0.0045, Figure 5K). Taken together, LBE promoted microglial phagocytosis and degradation of Aβ, which may partially explain the LBE‐mediated reduction of Aβ load in 5xFAD mice.

### 3.6 LBE Preserved Synapses and Neuronal Survival in the CNS of 5xFAD Mice

**FIGURE 6:**
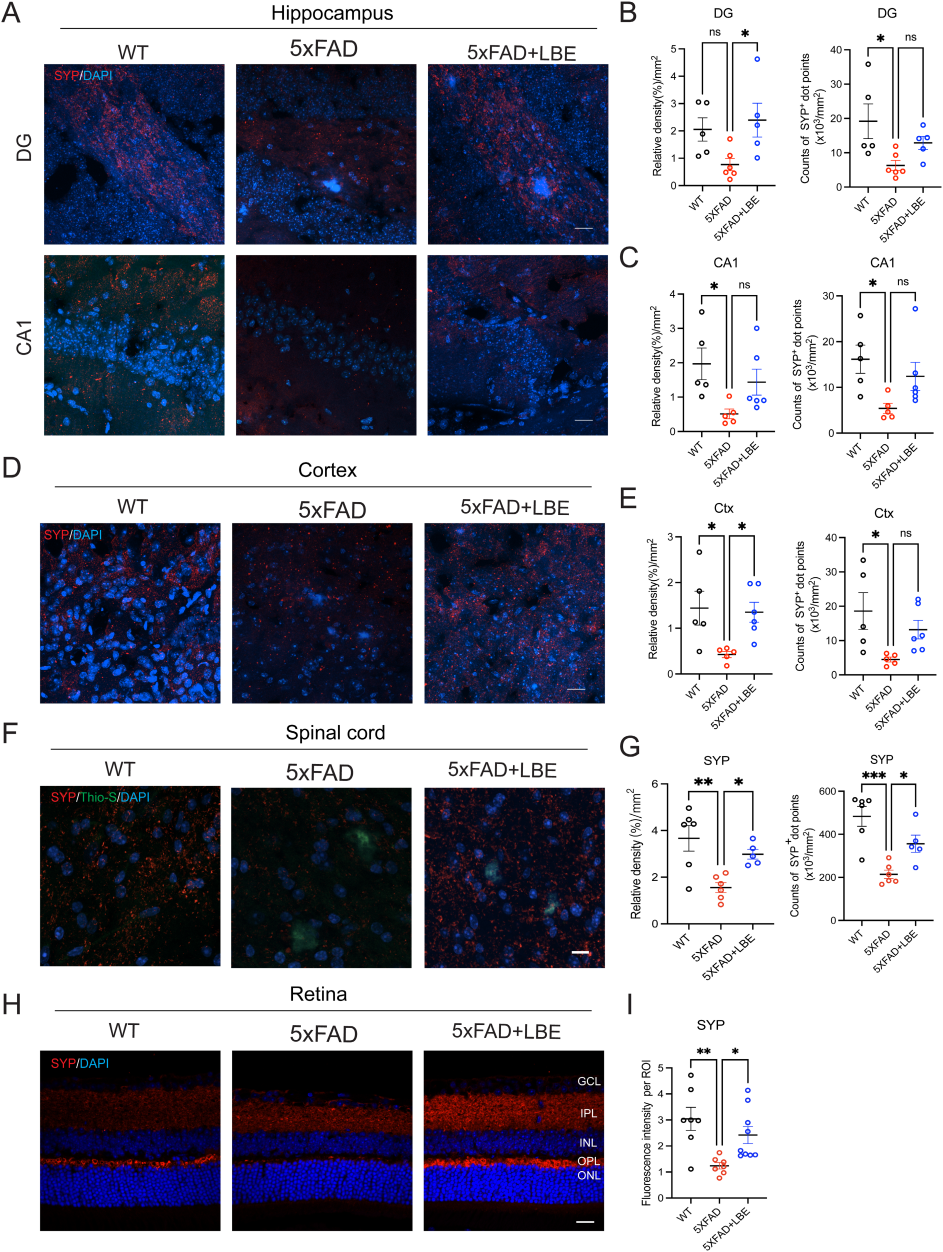
LBE treatment preserved presynaptic density in the CNS of 5xFAD mice. (A) Images of brain slices stained SYP (red) labeling presynaptic density from WT, 5xFAD, and 5xFAD + LBE mice. (B) Quantification of the relative density and counts of SYP‐positive dot points in the DG region. (C) Quantification of the relative density and counts of SYP‐positive dot points in the CA1 region. Scale bar: 20 μm. (D) Images of brain slices stained with SYP (red) labeling presynaptic density from WT, 5xFAD, and 5xFAD + LBE mice. (E) Quantification of the relative density and counts of SYP‐positive dot points in the cortex region. (F) Images of spinal cord slices stained with SYP (red) labeling presynaptic density from WT, 5xFAD, and 5xFAD + LBE mice. Scale bar: 20 μm. (G) Quantification of the relative density and counts of SYP‐positive dot points in the spinal cord region. (H) Images of retina slices stained with SYP (red) labeling presynaptic density merged with DAPI from WT, 5xFAD, and 5xFAD + LBE mice. (I) Quantification of the mean fluorescence intensity of SYP‐positive cells in the retina. Scale bar: 20 μm. Data are shown as mean ± SEM. *p < 0.05; **p < 0.01; ***p < 0.001 by one‐way ANOVA test with Dunn's multiple comparison test and unpaired two‐tailed Student's *t*‐test with two groups.

Previous studies have shown that microglial activation mediates synapse loss, leading to neurodegeneration and behavioral impairment. To determine whether the neuroprotective (Arg‐1^+^) microglia observed in 5xFAD mice treated with LBE were associated with changes in synaptic deficits, we evaluated the expression of presynaptic protein in the CNS, including brain, spinal cord, and retina (Figure 6). The relative density and number of SYP^+^ dot points in the hippocampus and cortex of 5xFAD mice were significantly decreased compared to WT mice (Figure 6A,D). After 2 months of LBE feeding, the relative density significantly increased in the DG (*p = 0.0444 vs. water‐fed group; Figure 6B) and cortex (*p = 0.0483 vs. water‐fed group; Figure 6E), and partially reversed the number of SYP^+^ dot points in the CA1 (*p* = 0.1876; Figure 6C) and cortex (*p* = 0.1977; Figure 6E). Additionally, LBE treatment increased the expression of SYP in the hippocampus (**p = 0.0017; Figure S5A,B).

The presynaptic protein SYP in the spinal cord of 5xFAD mice exhibited a significant reduction in both number and density at the ventral horn compared to WT mice (Figure 6F). The number reduced from 483.5 ± 46.16 (×10^3^/mm^2^) to 214.5 ± 19.29 (×10^3^/mm^2^) (***p < 0.001; Figure 6G), and the density reduced from 3.67 ± 0.55 to 1.55 ± 0.21(***p = 0.0030; Figure 6G). LBE feeding significantly reversed the SYP expression to 356.2 ± 40.10 (×10^3^/mm^2^) in number (*p = 0.0449; Figure 6G), and 2.98% ± 0.21% in density (*p = 0.0485; Figure 6G). In the retina, the fluorescence intensity of SYP at the IPL and OPL of LBE‐fed group significantly increased compared to water‐fed group (*p = 0.0457; Figure 6H,I).

For the postsynaptic protein of PSD95, 5xFAD mice demonstrated a slight decline in the relative density and number of PSD95+ dot points compared to WT mice (Figure S6A). Treatment with LBE significantly reversed the number of PSD95^+^ dot points in the CA1(**p = 0.0021 vs. water‐fed group; Figure S6C) and cortex (*p = 0.0313 vs. water‐fed group; Figure S6E), as well as the increased the relative density in the cortex (*p = 0.0113 vs. water‐fed group; Figure S6E). No significant difference was found in the DG region (Figure S6B). Similar to the changes in the brain, the number of PSD95^+^ dot points in the spinal cord of 5xFAD mice significantly decreased from a normal level 12.41 ± 1.87(×10^3^/mm^2^) to 6.24 ± 0.55(×10^3^/mm^2^) (**p = 0.0072; Figure 4F,G). LBE feeding significantly enhanced the PSD95 number to 10.65 ± 0.90 (×10^3^/mm^2^) (*p = 0.0296; Figure 4G). Considering that AD patients have reduced levels of PSD95 and SYP, these proteins were important regulation of synaptic plasticity. The increase of these protein levels in the CNS may contribute to improved cognitive behavior, motor movement, and visual function.

**FIGURE 7:**
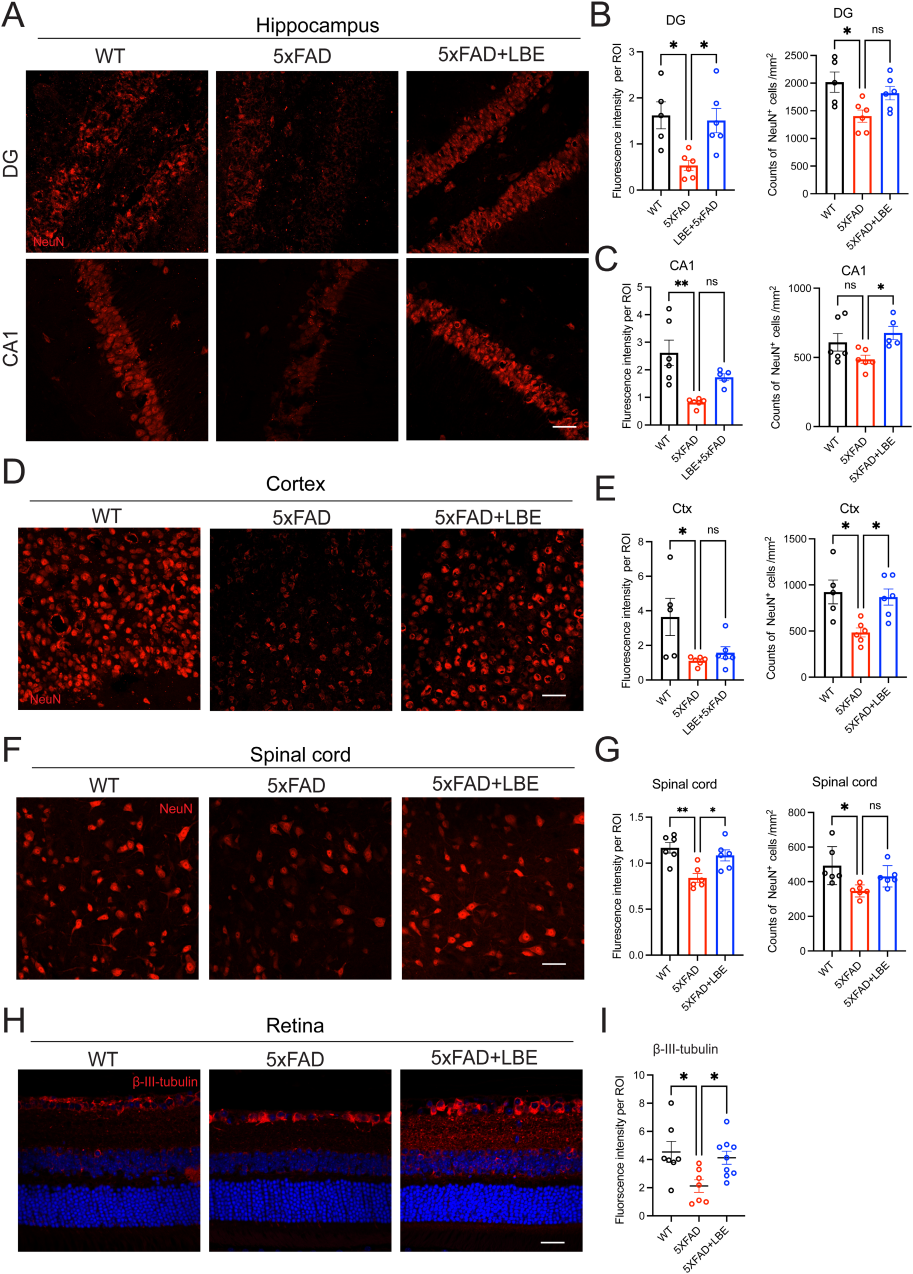
LBE treatment increased the number of neurons in CNS of 5xFAD mice. (A) Images of brain slices stained for NeuN labeling neurons from WT, 5xFAD, and 5xFAD + LBE mice. Brain regions including DG and CA1 in the hippocampus are shown in the different rows. Scale bar: 10 μm. (B) Quantification of the fluorescence intensity and number of NeuN‐positive neurons in the DG region. (*n* = 5 WT group, *n* = 6 5xFAD group, *n* = 6 5xFAD + LBE group). (C) Quantification of the fluorescence intensity and number of NeuN‐positive neurons in CA1 region. *n* = 6 WT group, *n* = 6 5xFAD group, *n* = 5 5xFAD + LBE group). (D) Images of brain slices stained for NeuN labeling neurons in the cortex from WT, 5xFAD, and 5xFAD + LBE mice. Scale bar: 50 μm. (E) Quantification of the fluorescence intensity and number of NeuN‐positive neurons in the cortex region. (F) Images of the spinal cord slices stained NeuN(red) from WT, 5xFAD, and 5xFAD + LBE mice. (G) Quantification of fluorescence intensity and number of NeuN‐positive neurons in the spinal cord. (H) Images of the retina slices stained for NeuN (red) that labeling neurons from WT, 5xFAD, and 5xFAD + LBE mice. (I) Quantification of the fluorescence intensity of β‐III tubulin‐positive in the retina. Scale bar: 10 μm Data are shown as mean ± SEM. *p < 0.05; ***p < 0.001 by one‐way ANOVA test with Dunn's multiple comparison test.

Neuron loss is the major pathological feature of AD. We next examined the neuronal condition in the CNS. Compared to WT mice, 5xFAD mice demonstrated a significant decline in the fluorescence intensity and number of NeuN‐positive neurons (Figure 7). The fluorescence intensity of NeuN‐positive neurons in LBE‐treated group showed a substantial increase in the DG (*p = 0.020; Figure 7B) and CA1 (*p* = 0.1099; Figure 7C) compared to the water‐fed group. The number of NeuN‐positive neurons in LBE‐treated group significantly increased in the CA1 (*p = 0.0429; Figure 7C) and cortex (*p = 0.0196; Figure 7E). Additionally, the fluorescence intensity of NeuN‐positive neurons significantly increased in the spinal cord of LBE‐treated group compared to water‐fed group (*p = 0.0183; Figure 7F,G). Although the number of NeuN‐positive neurons showed an upward trend in LBE‐treated group, no significant difference was found compared to the water‐fed group (Figure 7G). In the retina, LBE feeding increased the fluorescence intensity of β‐III tubulin (*p = 0.0430; Figure 7H,I), which may contribute to synapse stability and enhance the light response in the retina. The increased levels of presynaptic and postsynaptic proteins, along with the neuronal numbers may contribute to improved behavior outcome in 5xFAD mice.

### 3.7 LBE Increased the Expression of p‐Akt1/2, p‐Erk1/2, and p‐CREB to Exert the Neuroprotection in the CNS (Brain, Spinal Cord, and Retina) of 5xFAD Mice

**FIGURE 8:**
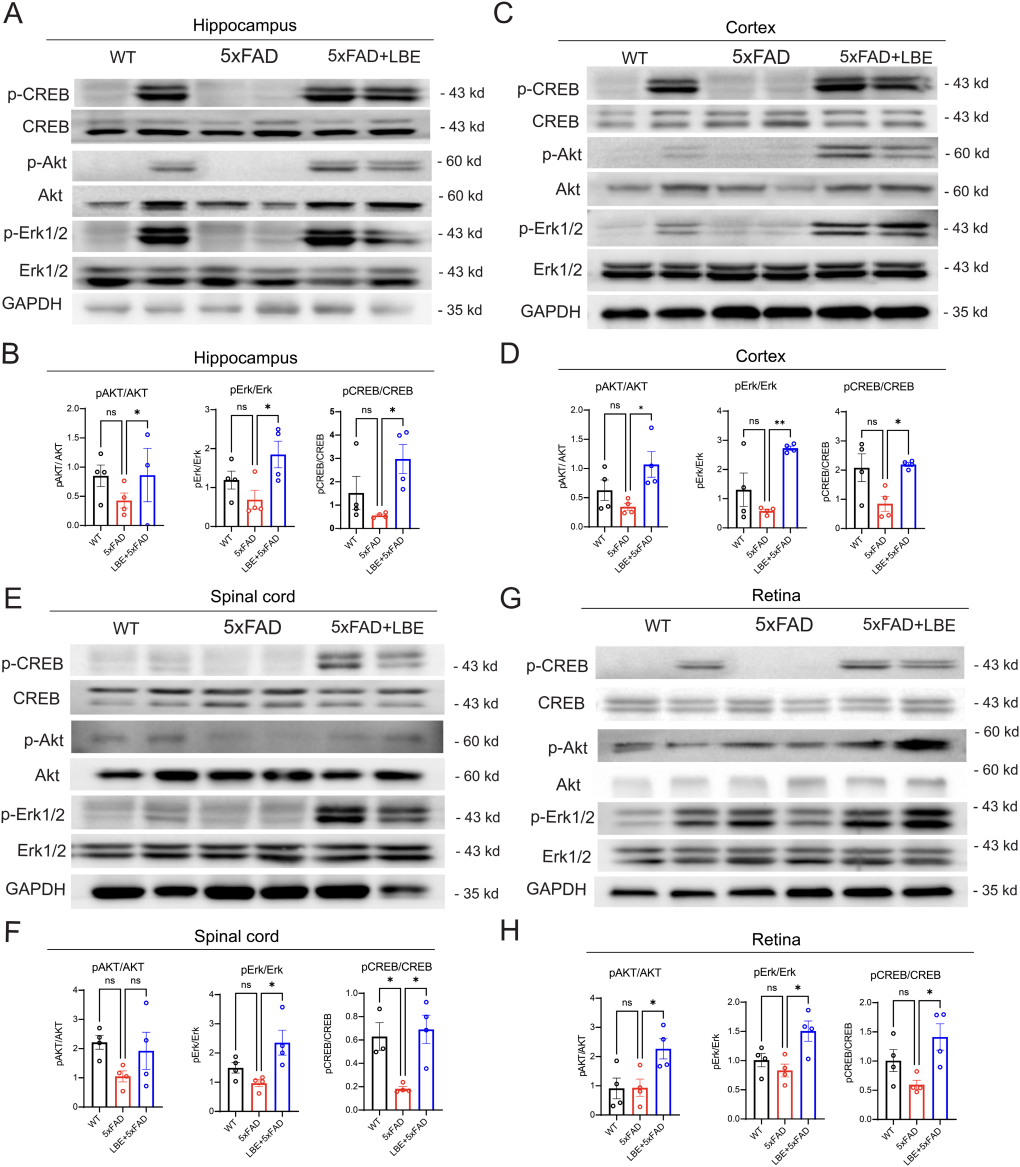
LBE treatment boosted the neuroprotective signaling pathways in CNS of 5xFAD mice. (A) Representative images of antibodies for p‐Erk1/2, p‐Akt, and p‐CREB in the hippocampus after LBE treatment. (B) Qualification of protein levels of p‐Erk1/2, p‐Akt, and p‐CREB expressions in the hippocampus. (C) Representative images of antibodies for p‐Erk1/2, p‐Akt, and p‐CREB in the cortex after LBE treatment. (D) Qualification of protein levels of p‐Akt, p‐Erk1/2, and p‐CREB in the cortex. (E) Representative images of antibodies for p‐Erk1/2, p‐Akt, and p‐CREB in the spinal cord after LBE treatment. (F) Qualification of protein levels of p‐Akt, p‐Erk1/2, and p‐CREB in the spinal cord. (G) Representative images of antibodies for p‐Erk1/2, p‐Akt, and p‐CREB in the retina after LBE treatment. (H) Qualification of protein levels of p‐Akt, p‐Erk1/2, and p‐CREB in the retina. Data are shown as mean ± SEM. *p < 0.05; **p < 0.01 by one‐way ANOVA test with Tukey's multiple comparison test.

To further conform the neuroprotective (Arg‐1^+^) microglia on the damaged neurons, we investigated the intracellular mechanisms associated with neuroprotection. Previous studies have shown that the serine–threonine kinase Akt, MAPK signaling, and downstream transcription factors such as cAMP‐response element binding protein (CREB) play a pivotal role in neuronal survival, protection, and spine formation. Their activation protects against cellular stress and injury [27, 28]. Akt and Erk1/2 activation promoted cellular survival, enhancing cognition and spine formation by phosphorylation of CREB, which results in the upregulation of CREB target genes [28]. In the current study, the expressions of phosphorylated Erk1/2 (p‐Erk1/2) and phosphorylated Akt1/2 (p‐Akt) were significantly increased by LBE treatment in the hippocampus (*p* = 0.0975 with p‐Akt; *p = 0.0377 with p‐Erk1/2; Figure 8A,B) and cortex (*p = 0.0317 with p‐Akt; **p = 0.0036 with p‐Erk1/2; Figure 8C,D). There was also a significant increase in phosphorylated CREB (p‐CREB) in both the hippocampus (*p = 0.0304; Figure 8B) and cortex (*p = 0.0354; Figure 8D).

In the spinal cord, p‐Erk1/2 was significantly increased in the LBE group (*p = 0.0155 vs. water‐fed group; Figure 8E,F), along with a significant increase of p‐CREB (*p = 0.0107 vs. water‐feeding group; Figure 8E,F), while p‐Akt showed no significant difference (Figure 8E,F). Additionally, results in the retina showed that p‐Erk1/2 and p‐Akt were significantly increased by LBE treatment, as well as a significant increase in p‐CREB (Figure 8G,H). These results suggested that LBE may exert neuroprotective effects through these signaling pathways, with regulatory factors in the PI3K/Akt and MAPK/ERK pathway being essential for cell survival and neuroprotection of damaged neurons [29]. The activation of CREB played a critical role in adaptive neuronal responses, enhancing synaptic transmission, facilitating synaptic plasticity, and regulating learning and memory functions.

## 4 Discussion

AD is characterized by a chronic inflammatory process with severe immune and inflammatory response in the pathological progression. Activated microglia exhibit limited phagocytic activity in amyloid clearance and instead promote the release of inflammatory factors, ultimately exacerbating degenerative processes. In our previous study, LBE promoted M2 polarization and reduced oligomeric Aβ‐induced inflammatory reactions in microglia [22]. In this study, following LBE treatment, there was a significant increase in the modified microglial morphology towards an alternative state. This alternative microglial state enhanced the phagocytic clearance of Aβ deposits, reduced inflammatory responses, and preserved synapses, which may contribute to the observed improvement in cognitive outcomes.

Neuroinflammation is a pathological feature in various neurodegenerative diseases, including AD, Parkinson's disease, glaucoma, Huntington's disease, and amyotrophic lateral sclerosis [30, 31]. Microgliosis is one of the critical features of these neurodegenerative diseases. This study may offer new insights into the roles of LBE as a potential neuroprotective agent via promoting the M2 polarization that restoring it to an anti‐inflammatory state. The neuroprotective microglia not only increased the phagocytosis of Aβ (Figure 5) and enhancement of the synaptic density (Figure 6) but also increased the expression of the classical neuroprotection signaling pathways (Figure 8). As herbal medicine has obtained increasing attention for the treatment of neurodegenerative diseases, these new findings on LBE will facilitate the widespread application of traditional Chinese medicine in neurodegenerative diseases.

Plaque‐associated microglia, characterized by a larger cell soma and shorter, thicker, and fewer branches, exhibited compromised phagocytic function and promoted the release of cytokines, including IL‐1β, IL‐6, and TNF‐α. This led to the assembly of the NLRP3 inflammasome, further activating the inflammatory pathway. Evidence from the study indicated that TNF‐α could induce neuronal apoptosis and immune response, and interfere with intracellular Ca^2+^ homeostasis. Cytokines including IL‐6 and IL‐1β stimulated neuroinflammation and played an important role in AD pathogenesis [22, 30]. In this study, after LBE treatment, cytokines such as IL‐1β, IL‐6, and TNF‐a significantly decreased, and the ratio of Arg1^+^/iNOS expression increased significantly compared to water‐fed group. LBE promoted microglia phagocytosis and clearance of Aβ in the brain of 5xFAD mice and cultured microglial cells (Figure 5). In this study, LBE induced alternative microglia that enhanced the phagocyte clearance of Aβ deposits, consistent with the actions of M2 microglia, which reduced the deposition of Aβ both in vitro and in vivo [32, 33, 34].

The MWM test is influenced by multiple factors, including water temperature, the environment, as well as the inherent locomotor abilities of the mice [35, 36, 37]. In this study, 5xFAD mice showed a motor dysfunction (Figure 1I–L) and a shorter distance in open‐field study compared to WT mice (Figure 1I–L and Figure S1F). Thus, it is crucial to acknowledge that we cannot entirely rule out the possibility that compromised motor function may have influenced the MWM results. Furthermore, our observation that LBE treatment significantly increased the swim speed (with a statistical significance of *p = 0.0234 when compared to the water‐fed group, as noted in the Figure S1C) raises an intriguing hypothesis. It prompts us to consider whether the observed improvement in cognitive function following LBE treatment could potentially be mediated, at least in part, by an enhancement in motor abilities. However, definitive conclusions on this matter require further investigation.

Although the previous studies demonstrated significant improvements in scotopic ERG with LBE in both 3xTg‐AD and early‐stage 5xFAD mice [23, 38], our findings indicate that while LBE enhanced scotopic a‐wave and b‐wave amplitudes in scotopic 1.0 of middle‐stage 5xFAD mice, its effects on PhNR and scotopic 3.0 responses were limited. This suggests that ERG response may not fully capture the impact of LBE on retinal function in this model. Several factors, such as disease stage, mechanism of action, dosing regimen, and inter‐individual variability, may contribute to these mixed results.

While the overall protective effect of LBE on the CNS system of 5xFAD mice was observed, inconsistencies were noted across various regions such as the hippocampus, cortex, spinal cord, and retina. These discrepancies may be attributed to the inherent anatomical and physiological differences among these regions. The progression of the disease in the 5xFAD model may not be uniform across all CNS regions, which could influence the effectiveness of LBE. For example, the hippocampus, crucial for memory, often exhibits early pathological changes, whereas cortical involvement progresses more gradually. The spinal cord and retina, due to their unique microenvironments and cell types, may respond differently to LBE. Moreover, variation in LBE's distribution and metabolism within these regions could also contribute to the observed inconsistencies. The complex mechanisms of LBE, including anti‐inflammatory and neuroprotective pathways, may display distinct manifestations in different regions.

Synapse deficits and neuron loss are critical characteristics in the pathological process of AD. In previous studies, microglia medicated synapse loss by activating the complement system and INF‐γ in the brain [31, 39]. LBE treatment altered the microglia phenotype to a neuroprotective state, promoted synapse density, and increased the expression of p‐CREB in brain, spinal cord, and retina. In previous studies, Aβ accumulation‐induced learning and memory deficits were mediated by alterations in CREB function, and its downregulation was assumed to result in synaptic atrophy and neuronal death. Akt and Erk1/2 activation promoted cellular survival, enhanced cognition, and spine formation by phosphorylation CREB, resulting in the upregulation of CREB target genes [28]. LBE increased p‐Erk1/2 and p‐Akt, along with the expression of p‐CREB. The phosphorylation of CREB at ser133 led to the triggering of CREB signaling and transcriptional activation of CREB, which play a crucial role in gene transcription during synaptic plasticity [29, 40].

AD exhibits multiple pathological processes, and it is a significant challenge for one drug to cover all the possible molecular mechanisms in AD treatment [1]. Chinese medicine, including LBE, has drawn great attention from the investigator's interest owing to their multiple effects, such as the inhibition of Aβ aggregation, prevention of mitochondrial dysfunction and apoptosis, as well as their antioxidant and anti‐inflammatory properties. In the present study, LBE is specifically derived from the water‐soluble components of wolfberries, encompasses polysaccharides, glycopeptides, certain vitamins, amino acids, and minerals. LBP are recognized for their immune‐modulatory, antioxidant, and neuroprotective functions. For example, LBP reduced Aβ deposit burden, prevented cognitive decline, and restored synaptic plasticity in a mouse model of AD partly through enhancing the BDNF/TrkB/CREB pathway in the hippocampus [15, 41], which were similar with our current results. Additionally, LBP modulated APP processing by downregulating the expression of BACE1 and upregulating the expression of ADAM10, and inhibited the production of excessive ROS by inducing the SKN‐1‐mediated antioxidant system and FSHR‐1‐mediated mitochondrial unfolded protein response (mtUPR) in a *C. elegans* model of AD [42, 43]. Furthermore, LB glycopeptide (LbGp) inhibited stress‐induced anxiety disorders by relieving oxidative stress and ferroptosis in the medial prefrontal cortex [44]. LbGp mitigated astrocyte damage, demyelination, and microglial activation in NMOSD models, enhancing motor and visual functions by inhibiting the release of proinflammatory factors via NF‐κB suppression [45]. LBE was made from *LB* by simple grinding containing different kinds of bioactive component, which may further explain that LBE can promote neuroprotection and anti‐inflammatory by exerting multiple functions.

## 5 Conclusions

The administration of LBE ameliorated the neurological pathology of CNS (brain, spinal cord, and retina) in 5xFAD mice. The therapeutic effects of LBE were evident, as it reduced systematic inflammatory and behavioral abnormalities, including cognitive disorders, movement issues, and retina responses. LBE treatment activated multiple signaling pathways, such as PI3K/AKT/CREB, and MAPK/Erk1/2/CREB, thereby promoting the expressions of PSD95 and SYP and regulating synaptic plasticity. As a homology of medicine and food, this study further confirmed the therapeutic effect of LBE in the middle stage of AD and demonstrated that LBE was not only a good candidate for prevention but also a supplementary drug for treatment.

## Author Contributions

Kin Chiu and Zhongqing Sun contributed to the study concept and design. Zhongqing Sun performed experiments. Kin Chiu, Yong Hu, Zhongqing Sun, Jinfeng Liu, Zihang Chen, and Kwok‐Fai So analyzed data, discussed results, and provided important intellectual content throughout the study. Yong Hu and Kwok‐Fai So critiqued data and helped in manuscript editing. All authors read and approved the final manuscript.

## Conflicts of Interest

The authors declare no conflicts of interest.

## Supporting information


**Figure S1.** LBE oral feeding restores cognitive memory in 5xFAD mice. (A) The latency for the mice to reach the platform during the first 5 days of trials (*n* = 25 WT, *n* = 19 water treatment, *n* = 21 LBE treatment, **p < 0.01, two‐way ANOVA with Tukey's multiple comparison test). (B)Total distance of day 6 in the swimming pool. (C) Mean speed of day 6 in the swimming pool (*n* = 25 WT group, *n* = 19 5xFAD group, *n* = 21 5xFAD + LBE group, one‐way ANOVA with Tukey's multiple comparison test). (D) Illustration of the open‐field test that measured the exploratory and spontaneous locomotor activity. (E) Time spent in the center of the square box (*n* = 25 WT group, *n* = 19 5xFAD group, *n* = 20 5xFAD + LBE group, Kruskal–Wallis test with Dunn's multiple comparison test). (F) Total distance in the center of the square box (*n* = 25 WT group, *n* = 19 5xFAD group, *n* = 20 5xFAD + LBE group, one‐way ANOVA with Tukey's multiple comparison test). (G) Mean traveling speed in the square box (*n* = 25 WT group, *n* = 19 5xFAD group, *n* = 20 5xFAD + LBE group, one‐way ANOVA with Tukey's multiple comparison test). (H) Illustration of the NOR test that measured the recognition memory. (I) Time spent on two objectives of the NOR test. (J) Recognition index in the NOR test.
**Figure S2.** LBE oral feeding restores motor movement and retina responses in 5xFAD mice. (A) Representative picture of 5xFAD and WT mice in clasping test. The limbs exhibit clasping with curled toes in the 5xFAD mice at the position of white arrow. The limb clasping test was used to quantify deficits in corticospinal function. (B) Qualify the score of clasping tests. (C) Representative ERG waveform to a scotopic 0.01 cd.s/m^2^ flash under light adaptation of WT group (dark), 5xFAD (red), and 5xFAD + LBE (blue). (D, E) Scattered plots of the amplitude of a‐wave and b‐wave in the ERG of different groups under dark adaptation and flash in 0.01 cd.s/m^2^. (F) Representative ERG waveform to a scotopic 3.0 cd.s/m^2^ flash under light adaptation of WT group (dark), 5xFAD (red), and 5xFAD + LBE (blue). (G, H) Scattered plots of the amplitude of a‐wave and b‐wave in the ERG of different groups under dark adaptation and flash in 3.0 cd.s/m^2^.
**Figure S3.** LBE treatment reduced Aβ load in the brain and spinal cord of 5xFAD mice. (A) Images of brain slices stained for Thio‐S (green) labeling Aβ plaques from WT mice. Regions including DG and CA1 of the hippocampus, and cortex are shown in the different rows for WT mice. (B) Images of spinal cord slices stained for Thio‐S (green) labeling Aβ plaques from WT mice. Scale bar: 50 μm.
**Figure S4.** LBE treatment reduced Aβ load in the brain 5xFAD mice. (A) Representative image of 4G8 antibody for amyloid plaques in the hippocampus. Qualification of 4G8 protein level in the hippocampus. (B) Representative image of Aβ1‐42 antibody for amyloid plaques in the hippocampus. Qualification of Aβ1‐42 protein level in the hippocampus. (C) Representative image of 4G8 antibody for amyloid plaques in the cortex. Qualification of 4G8 protein level in the cortex (*n* = 4 WT group, *n* = 4 5xFAD group, *n* = 4 5xFAD + LBE group, one‐way ANOVA with Tukey's multiple comparison test). (D) Representative image of 4G8 antibody for amyloid plaques in the spinal cord. Qualification of 4G8 protein level in the spinal cord. (E) Representative image of Aβ1‐42 antibody for amyloid plaques in the spinal cord. Qualification of Aβ1‐42 protein level in the spinal cord. (F) Representative image of 4G8 antibody for amyloid plaques in the retina. Qualification of 4G8 protein level in the retina (*n* = 4 WT group, *n* = 4 5xFAD group, *n* = 6 5xFAD + LBE group, one‐way ANOVA test with Tukey's post hoc test.) ns, not significant. *, *p* < 0.05; **, *p* < 0.01.
**Figure S5.** LBE treatment preserved presynaptic density in the CNS of 5xFAD mice. (A) Representative images of antibodies for SYP (presynapse) in brain after LBE treatment. (B) Qualification protein level of SYP in the hippocampus and cortex. Data are shown as mean ± SEM. *p < 0.05; **p < 0.01; ***p < 0.001 by one‐way ANOVA test with Dunn's multiple comparison test.
**Figure S6.** LBE treatment preserved postsynaptic density in the CNS of 5xFAD mice. (A) Images of brain slices stained PSD95 (red) labeling presynaptic density from WT, 5xFAD, and 5xFAD + LBE mice. (B) Quantification of the relative density and counts of PSD95‐positive dot points in the DG region. (C) Quantification of the relative density and counts of PSD95‐positive dot points in the CA1 region. Scale bar: 20 μm. (D) Images of brain slices stained PSD95 (red) labeling presynaptic density from WT, 5xFAD, and 5xFAD + LBE mice. (E) Quantification of the relative density and counts of PSD95‐positive dot points in the cortex region. (F) Images of spinal cord slices stained PSD95 (red) labeling presynaptic density from WT, 5xFAD, and 5xFAD + LBE mice. Scale bar: 20 μm. (G) Quantification of the relative density, and counts of PSD95‐positive dot points in the spinal cord. (H) Images of retina slices stained for PSD95 (red) labeling presynaptic density merged with DAPI from WT, 5xFAD, and 5xFAD + LBE mice. (I) Quantification of the fluorescence intensity of PSD95‐positive in the retina. Scale bar: 20 μm. Data are shown as mean ± SEM. *p < 0.05; **p < 0.01; ***p < 0.001 by one‐way ANOVA test with Tukey's multiple comparison test.

**Data S1.** Supporting Information.

## Data Availability

The data that support the findings of this study are available from the corresponding author upon reasonable request.

## References

[1] F. Leng and P. Edison , “Neuroinflammation and Microglial Activation in Alzheimer Disease: Where Do We Go From Here?,” Nature Reviews. Neurology 17, no. 3 (2021): 157–172. 33318676 10.1038/s41582-020-00435-y

[2] J. M. Long and D. M. Holtzman , “Alzheimer Disease: An Update on Pathobiology and Treatment Strategies,” Cell 179, no. 2 (2019): 312–339. 31564456 10.1016/j.cell.2019.09.001 PMC6778042

[3] L. Jia , M. Quan , Y. Fu , et al., “Dementia in China: Epidemiology, Clinical Management, and Research Advances,” Lancet Neurology 19, no. 1 (2020): 81–92. 31494009 10.1016/S1474-4422(19)30290-X

[4] C. H. van Dyck , C. J. Swanson , P. Aisen , et al., “Lecanemab in Early Alzheimer's Disease,” New England Journal of Medicine 388, no. 1 (2023): 9–21. 36449413 10.1056/NEJMoa2212948

[5] Y. Tang and W. Le , “Differential Roles of M1 and M2 Microglia in Neurodegenerative Diseases,” Molecular Neurobiology 53, no. 2 (2016): 1181–1194. 25598354 10.1007/s12035-014-9070-5

[6] G. Zhang , Z. Wang , H. Hu , M. Zhao , and L. Sun , “Microglia in Alzheimer's Disease: A Target for Therapeutic Intervention,” Frontiers in Cellular Neuroscience 15 (2021): 749587. 34899188 10.3389/fncel.2021.749587 PMC8651709

[7] H. Sarlus and M. T. Heneka , “Microglia in Alzheimer's Disease,” Journal of Clinical Investigation 127, no. 9 (2017): 3240–3249. 28862638 10.1172/JCI90606 PMC5669553

[8] O. Wirths , H. Breyhan , A. Marcello , M. C. Cotel , W. Bruck , and T. A. Bayer , “Inflammatory Changes Are Tightly Associated With Neurodegeneration in the Brain and Spinal Cord of the APP/PS1KI Mouse Model of Alzheimer's Disease,” Neurobiology of Aging 31, no. 5 (2010): 747–757. 18657882 10.1016/j.neurobiolaging.2008.06.011

[9] S. Jawhar , A. Trawicka , C. Jenneckens , T. A. Bayer , and O. Wirths , “Motor Deficits, Neuron Loss, and Reduced Anxiety Coinciding With Axonal Degeneration and Intraneuronal Aβ Aggregation in the 5XFAD Mouse Model of Alzheimer's Disease,” Neurobiology of Aging 33, no. 1 (2012): 196.e129–196.e140. 10.1016/j.neurobiolaging.2010.05.027 20619937

[10] S. Chiquita , A. C. Rodrigues‐Neves , F. I. Baptista , et al., “The Retina as a Window or Mirror of the Brain Changes Detected in Alzheimer's Disease: Critical Aspects to Unravel,” Molecular Neurobiology 56, no. 8 (2019): 5416–5435. 30612332 10.1007/s12035-018-1461-6

[11] F. Liu , J. Zhang , Z. Xiang , et al., “ *Lycium barbarum* Polysaccharides Protect Retina in rd1 Mice During Photoreceptor Degeneration,” Investigative Ophthalmology & Visual Science 59, no. 1 (2018): 597–611. 29372259 10.1167/iovs.17-22881 PMC6623178

[12] S. E. Perez , S. Lumayag , B. Kovacs , E. J. Mufson , and S. Xu , “Beta‐Amyloid Deposition and Functional Impairment in the Retina of the APPswe/PS1DeltaE9 Transgenic Mouse Model of Alzheimer's Disease,” Investigative Ophthalmology & Visual Science 50, no. 2 (2009): 793–800. 18791173 10.1167/iovs.08-2384 PMC3697019

[13] A. L. Manthey , K. Chiu , and K. F. So , “Effects of *Lycium barbarum* on the Visual System,” International Review of Neurobiology 135 (2017): 1–27. 28807155 10.1016/bs.irn.2017.02.002

[14] Y. S. Ho , M. S. Yu , X. F. Yang , K. F. So , W. H. Yuen , and R. C. Chang , “Neuroprotective Effects of Polysaccharides From Wolfberry, the Fruits of *Lycium barbarum* , Against Homocysteine‐Induced Toxicity in Rat Cortical Neurons,” Journal of Alzheimer's Disease 19, no. 3 (2010): 813–827. 10.3233/JAD-2010-1280 20157238

[15] Y. Zhou , Y. Duan , S. Huang , et al., “Polysaccharides From *Lycium barbarum* Ameliorate Amyloid Pathology and Cognitive Functions in APP/PS1 Transgenic Mice,” International Journal of Biological Macromolecules 144 (2020): 1004–1012. 31715236 10.1016/j.ijbiomac.2019.09.177

[16] M. Ye , J. Moon , J. Yang , et al., “The Standardized *Lycium chinense* Fruit Extract Protects Against Alzheimer's Disease in 3xTg‐AD Mice,” Journal of Ethnopharmacology 172 (2015): 85–90. 26102549 10.1016/j.jep.2015.06.026

[17] S. Y. Liu , S. Lu , X. L. Yu , et al., “Fruitless Wolfberry‐Sprout Extract Rescued Cognitive Deficits and Attenuated Neuropathology in Alzheimer's Disease Transgenic Mice,” Current Alzheimer Research 15, no. 9 (2018): 856–868. 29623840 10.2174/1567205015666180404160625

[18] K. Wang , J. Xiao , B. Peng , et al., “Retinal Structure and Function Preservation by Polysaccharides of Wolfberry in a Mouse Model of Retinal Degeneration,” Scientific Reports 4 (2014): 7601. 25535040 10.1038/srep07601 PMC4274520

[19] P. Teng , Y. Li , W. Cheng , L. Zhou , Y. Shen , and Y. Wang , “Neuroprotective Effects of *Lycium barbarum* Polysaccharides in Lipopolysaccharide‐Induced BV2 Microglial Cells,” Molecular Medicine Reports 7, no. 6 (2013): 1977–1981. 23620217 10.3892/mmr.2013.1442

[20] J. Xiao , E. C. Liong , Y. P. Ching , et al., “ *Lycium barbarum* Polysaccharides Protect Mice Liver From Carbon Tetrachloride‐Induced Oxidative Stress and Necroinflammation,” Journal of Ethnopharmacology 139, no. 2 (2012): 462–470. 22138659 10.1016/j.jep.2011.11.033

[21] Y. K. Zhang , J. Wang , L. Liu , R. C. Chang , K. F. So , and G. Ju , “The Effect of *Lycium barbarum* on Spinal Cord Injury, Particularly Its Relationship With M1 and M2 Macrophage in Rats,” BMC Complementary and Alternative Medicine 13 (2013): 67. 23517687 10.1186/1472-6882-13-67 PMC3618261

[22] Z. Q. Sun , J. F. Liu , W. Luo , et al., “ *Lycium barbarum* Extract Promotes M2 Polarization and Reduces Oligomeric Amyloid‐β‐Induced Inflammatory Reactions in Microglial Cells,” Neural Regeneration Research 17, no. 1 (2022): 203–209. 34100457 10.4103/1673-5374.314325 PMC8451572

[23] J. Liu , L. Baum , S. Yu , et al., “Preservation of Retinal Function Through Synaptic Stabilization in Alzheimer's Disease Model Mouse Retina by *Lycium Barbarum* Extracts,” Frontiers in Aging Neuroscience 13 (2021): 788798. 35095474 10.3389/fnagi.2021.788798 PMC8792986

[24] H. Lian , E. Roy , and H. Zheng , “Protocol for Primary Microglial Culture Preparation,” Bio‐Protocol 6, no. 21 (2016): e1989. 29104890 10.21769/BioProtoc.1989 PMC5669279

[25] R. C. McCarthy , D. Y. Lu , A. Alkhateeb , A. M. Gardeck , C. H. Lee , and M. Wessling‐Resnick , “Characterization of a Novel Adult Murine Immortalized Microglial Cell Line and Its Activation by Amyloid‐Beta,” Journal of Neuroinflammation 13 (2016): 21. 26819091 10.1186/s12974-016-0484-z PMC4730646

[26] F. Kosel , J. M. S. Pelley , and T. B. Franklin , “Behavioural and Psychological Symptoms of Dementia in Mouse Models of Alzheimer's Disease‐Related Pathology,” Neuroscience and Biobehavioral Reviews 112 (2020): 634–647. 32070692 10.1016/j.neubiorev.2020.02.012

[27] K. Tanaka , S. Nogawa , E. Nagata , et al., “Persistent CREB Phosphorylation With Protection of Hippocampal CA1 Pyramidal Neurons Following Temporary Occlusion of the Middle Cerebral Artery in the Rat,” Experimental Neurology 161, no. 2 (2000): 462–471. 10686068 10.1006/exnr.1999.7313

[28] M. R. Walton and I. Dragunow , “Is CREB a Key to Neuronal Survival?,” Trends in Neurosciences 23, no. 2 (2000): 48–53. 10652539 10.1016/s0166-2236(99)01500-3

[29] M. Amidfar , J. de Oliveira , E. Kucharska , J. Budni , and Y. K. Kim , “The Role of CREB and BDNF in Neurobiology and Treatment of Alzheimer's Disease,” Life Sciences 257 (2020): 118020. 32603820 10.1016/j.lfs.2020.118020

[30] A. Griciuc and R. E. Tanzi , “The Role of Innate Immune Genes in Alzheimer's Disease,” Current Opinion in Neurology 34, no. 2 (2021): 228–236. 33560670 10.1097/WCO.0000000000000911 PMC7954128

[31] W. Cao and H. Zheng , “Peripheral Immune System in Aging and Alzheimer's Disease,” Molecular Neurodegeneration 13, no. 1 (2018): 51. 30285785 10.1186/s13024-018-0284-2 PMC6169078

[32] J. D. Cherry , J. A. Olschowka , and M. K. O'Banion , “Arginase 1+ Microglia Reduce Aβ Plaque Deposition During IL‐1β‐Dependent Neuroinflammation,” Journal of Neuroinflammation 12 (2015): 203. 26538310 10.1186/s12974-015-0411-8 PMC4634600

[33] C. H. Latta , T. L. Sudduth , E. M. Weekman , et al., “Determining the Role of IL‐4 Induced Neuroinflammation in Microglial Activity and Amyloid‐β Using BV2 Microglial Cells and APP/PS1 Transgenic Mice,” Journal of Neuroinflammation 12 (2015): 41. 25885682 10.1186/s12974-015-0243-6 PMC4350455

[34] A. Lyons , R. J. Griffin , C. E. Costelloe , R. M. Clarke , and M. A. Lynch , “IL‐4 Attenuates the Neuroinflammation Induced by Amyloid‐Beta In Vivo and In Vitro,” Journal of Neurochemistry 101, no. 3 (2007): 771–781. 17250684 10.1111/j.1471-4159.2006.04370.x

[35] H. Tian , N. Ding , M. Guo , et al., “Analysis of Learning and Memory Ability in an Alzheimer's Disease Mouse Model Using the Morris Water Maze,” Journal of Visualized Experiments 152 (2019). 10.3791/60055 31736488

[36] C. V. Vorhees and M. T. Williams , “Morris Water Maze: Procedures for Assessing Spatial and Related Forms of Learning and Memory,” Nature Protocols 1, no. 2 (2006): 848–858. 17406317 10.1038/nprot.2006.116 PMC2895266

[37] N. Curdt , F. W. Schmitt , C. Bouter , et al., “Search Strategy Analysis of Tg4‐42 Alzheimer Mice in the Morris Water Maze Reveals Early Spatial Navigation Deficits,” Scientific Reports 12, no. 1 (2022): 5451. 35361814 10.1038/s41598-022-09270-1 PMC8971530

[38] L. Zhong , K. Chiu , G.‐S. He , and Y. Xu , “Wolfberry Extract Enhances the Retinal Light Responses of a Mouse Model of Alzheimer's Disease,” Journal of Pharmaceutical and Biomedical Sciences 11, no. 2 (2021).

[39] E. R. Roy , B. Wang , Y. W. Wan , et al., “Type I Interferon Response Drives Neuroinflammation and Synapse Loss in Alzheimer Disease,” Journal of Clinical Investigation 130, no. 4 (2020): 1912–1930. 31917687 10.1172/JCI133737 PMC7108898

[40] S. Cohen and M. E. Greenberg , “Communication Between the Synapse and the Nucleus in Neuronal Development, Plasticity, and Disease,” Annual Review of Cell and Developmental Biology 24 (2008): 183–209. 10.1146/annurev.cellbio.24.110707.175235 PMC2709812 18616423

[41] Y. Gao , Y. Wei , Y. Wang , F. Gao , and Z. Chen , “ *Lycium barbarum* : A Traditional Chinese Herb and a Promising Anti‐Aging Agent,” Aging and Disease 8, no. 6 (2017): 778–791. 29344416 10.14336/AD.2017.0725 PMC5758351

[42] L. Zhou , W. Liao , H. Zeng , Y. Yao , X. Chen , and K. Ding , “A Pectin From Fruits of *Lycium barbarum* L. Decreases β‐Amyloid Peptide Production Through Modulating APP Processing,” Carbohydrate Polymers 201 (2018): 65–74. 30241864 10.1016/j.carbpol.2018.08.050

[43] J. Meng , Z. Lv , M. Guo , et al., “A *Lycium barbarum* Extract Inhibits β‐Amyloid Toxicity by Activating the Antioxidant System and mtUPR in a *Caenorhabditis elegans* Model of Alzheimer's Disease,” FASEB Journal 36, no. 2 (2022): e22156. 35044707 10.1096/fj.202101116RR

[44] Y. Dai , J. Guo , B. Zhang , et al., “ *Lycium barbarum* (Wolfberry) Glycopeptide Prevents Stress‐Induced Anxiety Disorders by Regulating Oxidative Stress and Ferroptosis in the Medial Prefrontal Cortex,” Phytomedicine 116 (2023): 154864. 37182278 10.1016/j.phymed.2023.154864

[45] L. Xu , L. Yang , H. Xu , et al., “ *Lycium barbarum* Glycopeptide Ameliorates Motor and Visual Deficits in Autoimmune Inflammatory Diseases,” Phytomedicine 129 (2024): 155610. 38640861 10.1016/j.phymed.2024.155610

