# Supplementary material for: Lycium barbarum Extract Enhanced Neuroplasticity and Functional Recovery in 5xFAD Mice via Modulating Microglial Status of the Central Nervous System
Source: CNS Neurosci Ther. 2024 Nov 20;30(11):e70123. doi: 10.1111/cns.70123 (PMC11576918; doi:10.1111/cns.70123)
Supplement: Supplementary file 1 — Figure S1. LBE oral feeding restores cognitive memory in 5xFAD mice. (A) The latency for the mice to reach the platform during the first 5 days of trials (n = 25 WT, n = 19 water treatment, n = 21 LBE treatment, **p < 0.01, two‐way ANOVA with Tukey's multiple comparison test). (B)Total distance of day 6 in the swimming pool. (C) Mean speed of day 6 in the swimming pool (n = 25 WT group, n = 19 5xFAD group, n = 21 5xFAD + LBE group, one‐way ANOVA with Tukey's multiple comparison test). (D) Illustration of the open‐field test that measured the exploratory and spontaneous locomotor activity. (E) Time spent in the center of the square box (n = 25 WT group, n = 19 5xFAD group, n = 20 5xFAD + LBE group, Kruskal–Wallis test with Dunn's multiple comparison test). (F) Total distance in the center of the square box (n = 25 WT group, n = 19 5xFAD group, n = 20 5xFAD + LBE group, one‐way ANOVA with Tukey's multiple comparison test). (G) Mean traveling speed in the square box (n = 25 WT group, n = 19 5xFAD group, n = 20 5xFAD + LBE group, one‐way ANOVA with Tukey's multiple comparison test). (H) Illustration of the NOR test that measured the recognition memory. (I) Time spent on two objectives of the NOR test. (J) Recognition index in the NOR test. Figure S2. LBE oral feeding restores motor movement and retina responses in 5xFAD mice. (A) Representative picture of 5xFAD and WT mice in clasping test. The limbs exhibit clasping with curled toes in the 5xFAD mice at the position of white arrow. The limb clasping test was used to quantify deficits in corticospinal function. (B) Qualify the score of clasping tests. (C) Representative ERG waveform to a scotopic 0.01 cd.s/m2 flash under light adaptation of WT group (dark), 5xFAD (red), and 5xFAD + LBE (blue). (D, E) Scattered plots of the amplitude of a‐wave and b‐wave in the ERG of different groups under dark adaptation and flash in 0.01 cd.s/m2. (F) Representative ERG waveform to a scotopic 3.0 cd.s/m2 flash under light adaptation of [file CNS-30-e70123-s002.docx]

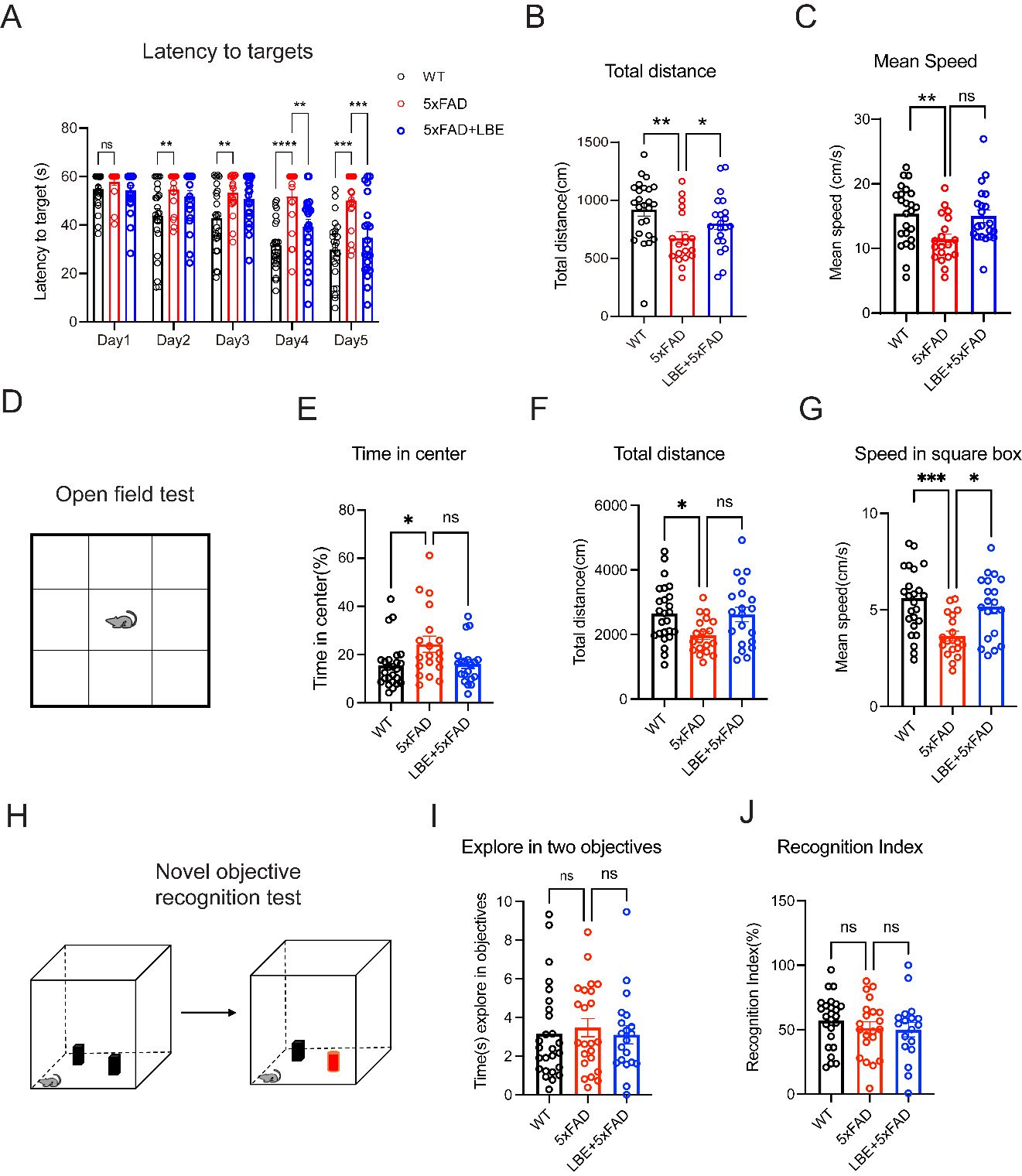


**SFigure 1. LBE oral feeding restores cognitive memory in 5xFAD mice.** (A) The latency for the mice to reach the platform during the first 5 days of trials (n=25 WT, n=19 water treatment, n=21 LBE treatment, **p<0.01, two-way ANOVA with Tukey’s multiple comparison test). (B)Total distance of day 6 in the swimming pool. (C) Mean speed of day 6 in the swimming pool. (n=25 WT group, n=19 5xFAD group, n=21 5xFAD+LBE group, one-way ANOVA with Tukey’s multiple comparison test). (D) Illustration of the open field test that measured the exploratory and spontaneous locomotor activity. (E) Time spent in the center of the square box. (n=25 WT group, n=19 5xFAD group, n=20 5xFAD+LBE group, Kruskal-Wallis test with Dunn’s multiple comparison test). (F) Total distance in the center of the square box. (n=25 WT group, n=19 5xFAD group, n=20 5xFAD+LBE group, one-way ANOVA with Tukey’s multiple comparison test). (G) Mean traveling speed in the square box. (n=25 WT group, n=19 5xFAD group, n=20 5xFAD+LBE group, one-way ANOVA with Tukey’s multiple comparison test). (H) Illustration of the NOR test that measured the recognition memory. (I) Time spent in two objectives of the NOR test. (J) Recognition index in the NOR test.

**
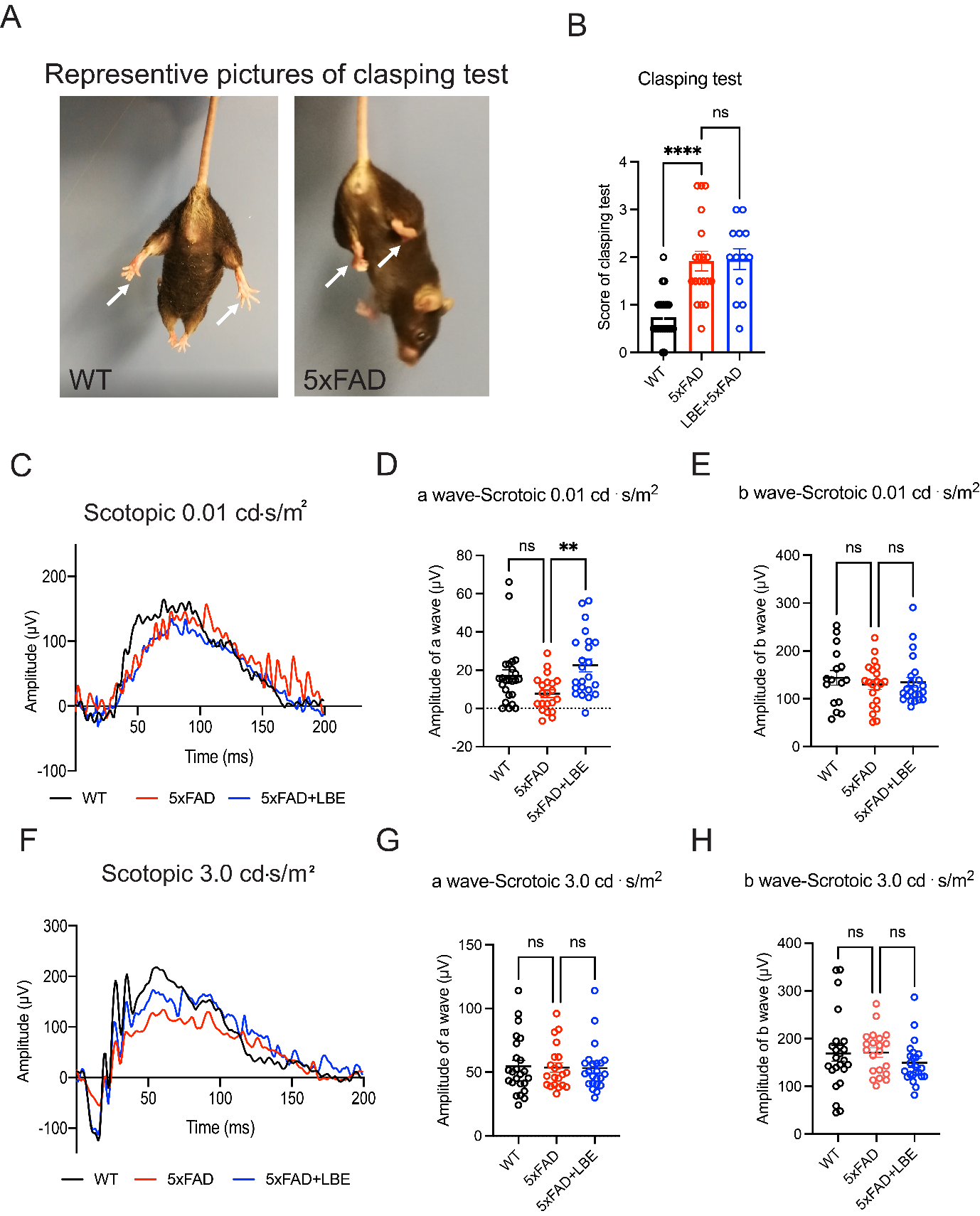
**

**SFigure 2. LBE oral feeding restores motor movement and retina responses in 5xFAD mice.** (A) Representative picture of 5xFAD and WT mice in clasping test. The limbs exhibit clasping with curled toes in the 5xFAD mice at the position of white arrow. The limb clasping test was used to quantify deficits in corticospinal function. (B) Qualify the score of clasping tests. (C) Representative ERG waveform to a Scotopic 0.01 cd.s/m^2^ flash under light adaptation of WT group(dark), 5xFAD(red), and 5xFAD+LBE(blue). (D, E) Scattered plots of the amplitude of a-wave and b-wave in the ERG of different group under dark adaptation and flash in 0.01 cd.s/m2. (F) Representative ERG waveform to a Scotopic 3.0 cd.s/m^2^ flash under light adaptation of WT group(dark), 5xFAD(red), and 5xFAD+LBE(blue). (G, H) Scattered plots of the amplitude of a-wave and b-wave in the ERG of different group under dark adaptation and flash in 3.0 cd.s/m^2^.


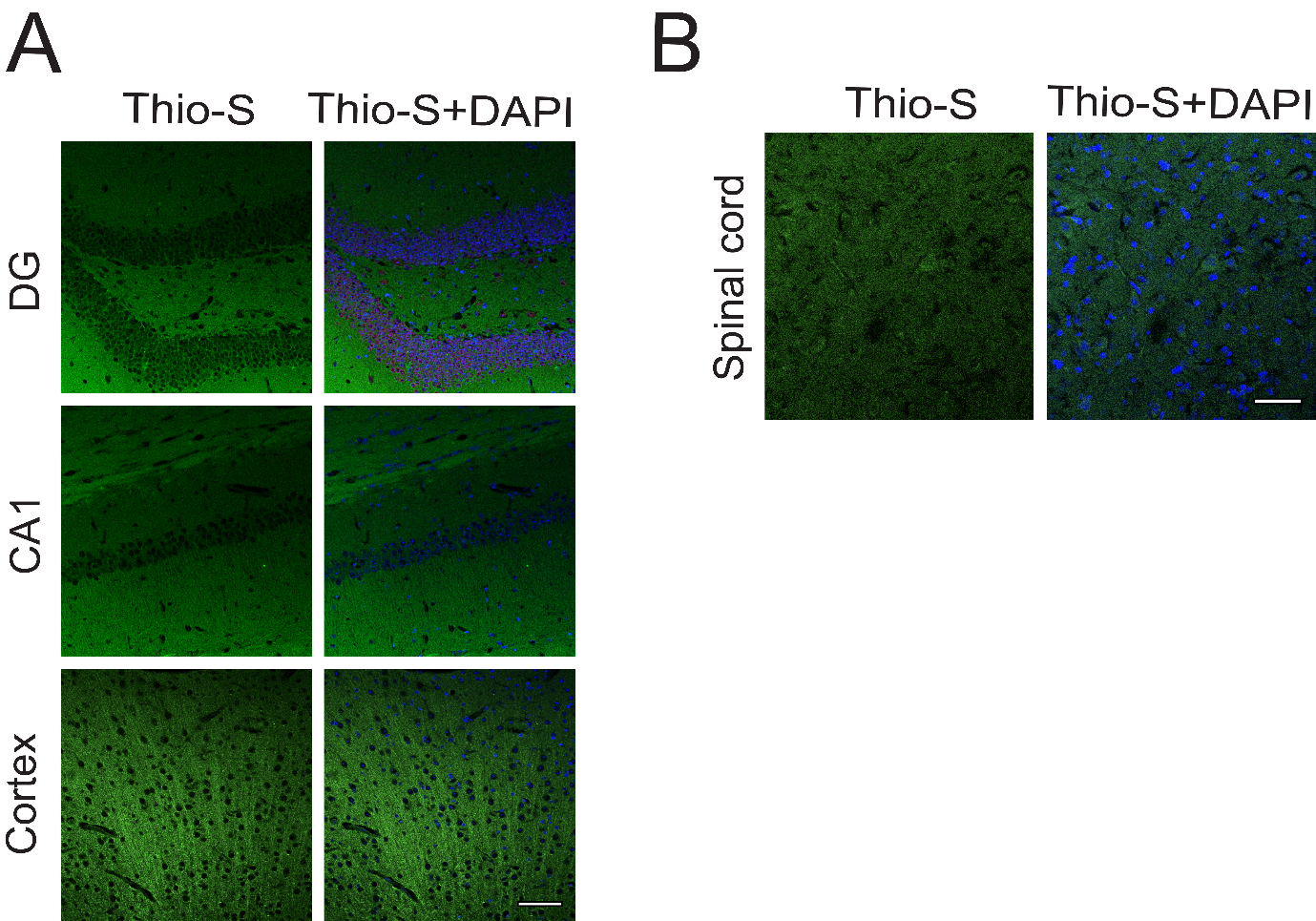


**SFigure 3. LBE treatment reduced Aβ load in the brain and spinal cord of 5xFAD mice.** (A) Images of brain slices stained for Thio-S (green) labeling Aβ plaques from WT mice. Regions including DG and CA1 of the hippocampus, and cortex were showed in the different rows for WT mice. (B) Images of spinal cord slices stained for Thio-S (green) labeling Aβ plaques from WT mice. Scale bar:50μm.


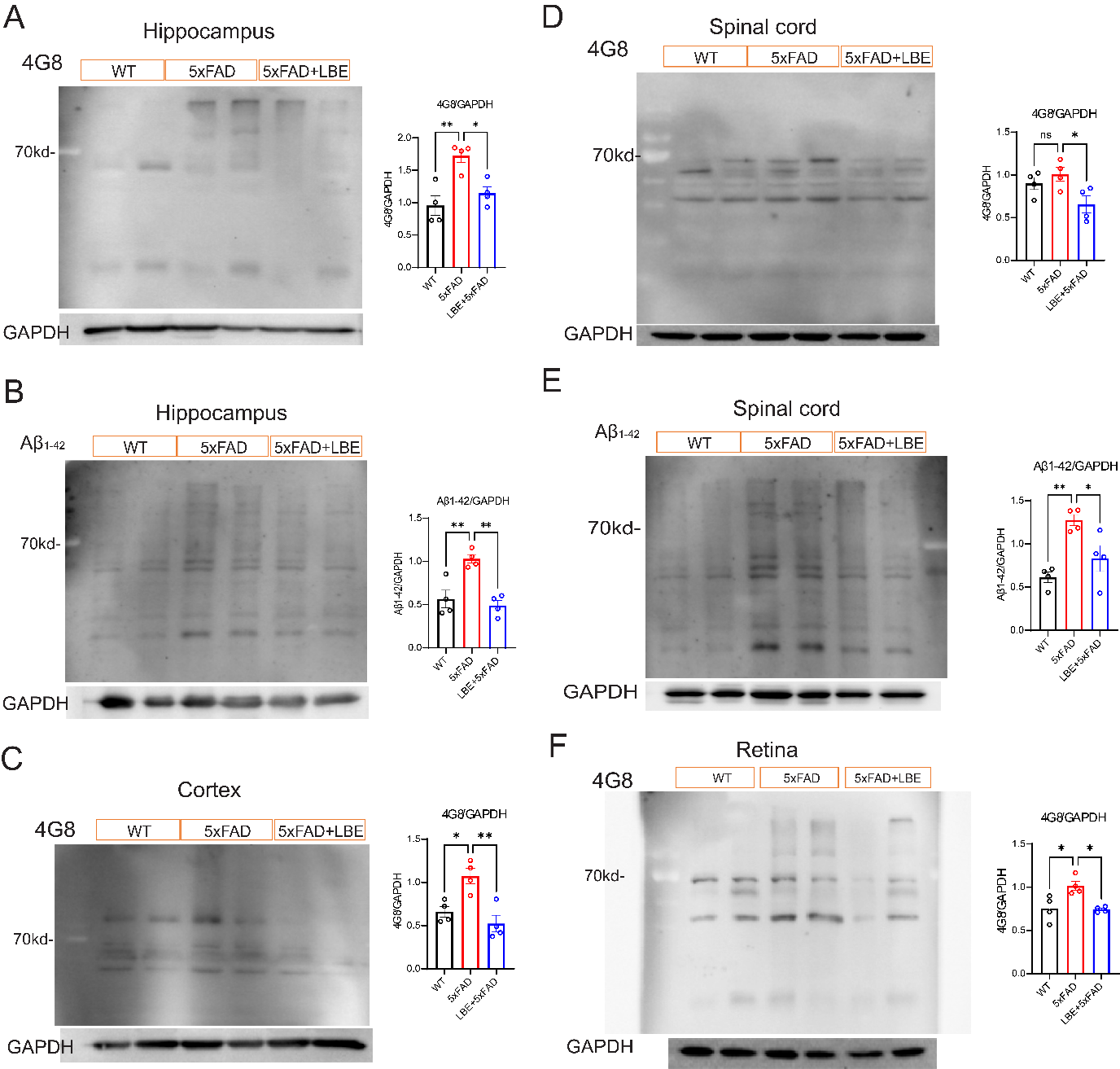


**SFigure 4. LBE treatment reduced Aβ load in the brain 5xFAD mice.** (A) Representative image of 4G8 antibody for amyloid plaques in the hippocampus. Qualification of 4G8 protein level in the hippocampus. (B) Representative image of Aβ1-42 antibody for amyloid plaques in the hippocampus. Qualification of Aβ1-42 protein level in the hippocampus. (C) Representative image of 4G8 antibody for amyloid plaques in the cortex. Qualification of 4G8 protein level in the cortex. (n=4 WT group, n=4 5xFAD group, n=4 5xFAD+LBE group, one-way ANOVA with Tukey’s multiple comparison test). (D) Representative image of 4G8 antibody for amyloid plaques in the spinal cord. Qualification of 4G8 protein level in the spinal cord. (E) Representative image of Aβ1-42 antibody for amyloid plaques in the spinal cord. Qualification of Aβ1-42 protein level in the spinal cord. (F) Representative image of 4G8 antibody for amyloid plaques in the retina. Qualification of 4G8 protein level in the retina. (n=4 WT group, n=4 5xFAD group, n=6 5xFAD+LBE group, one-way ANOVA test with Tukey’s post hoc test.) ns, not significant. *, p<0.05; **, p<0.01.


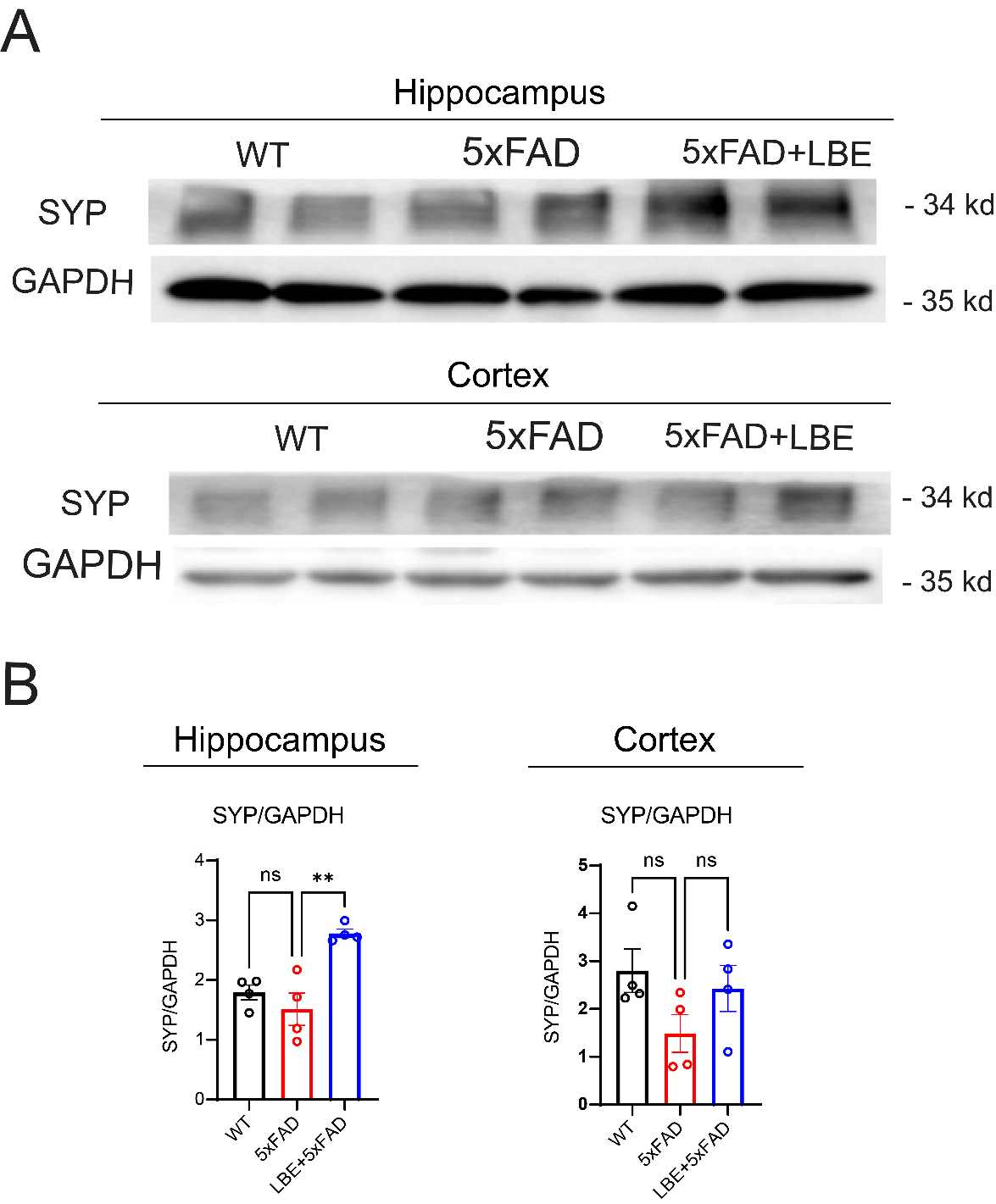


**SFigure 5. LBE treatment preserved presynaptic density in the CNS of 5xFAD mice.** (A) Representative images of antibodies for SYP (pre-synapse) in brain after LBE treatment. (B) Qualification proteins level of SYP in the hippocampus and cortex. Data was shown as mean ± SEM. *: p < 0.05; **: p < 0.01; ***: p < 0.001 by one-way ANOVA test with Dunn’s multiple comparison test.


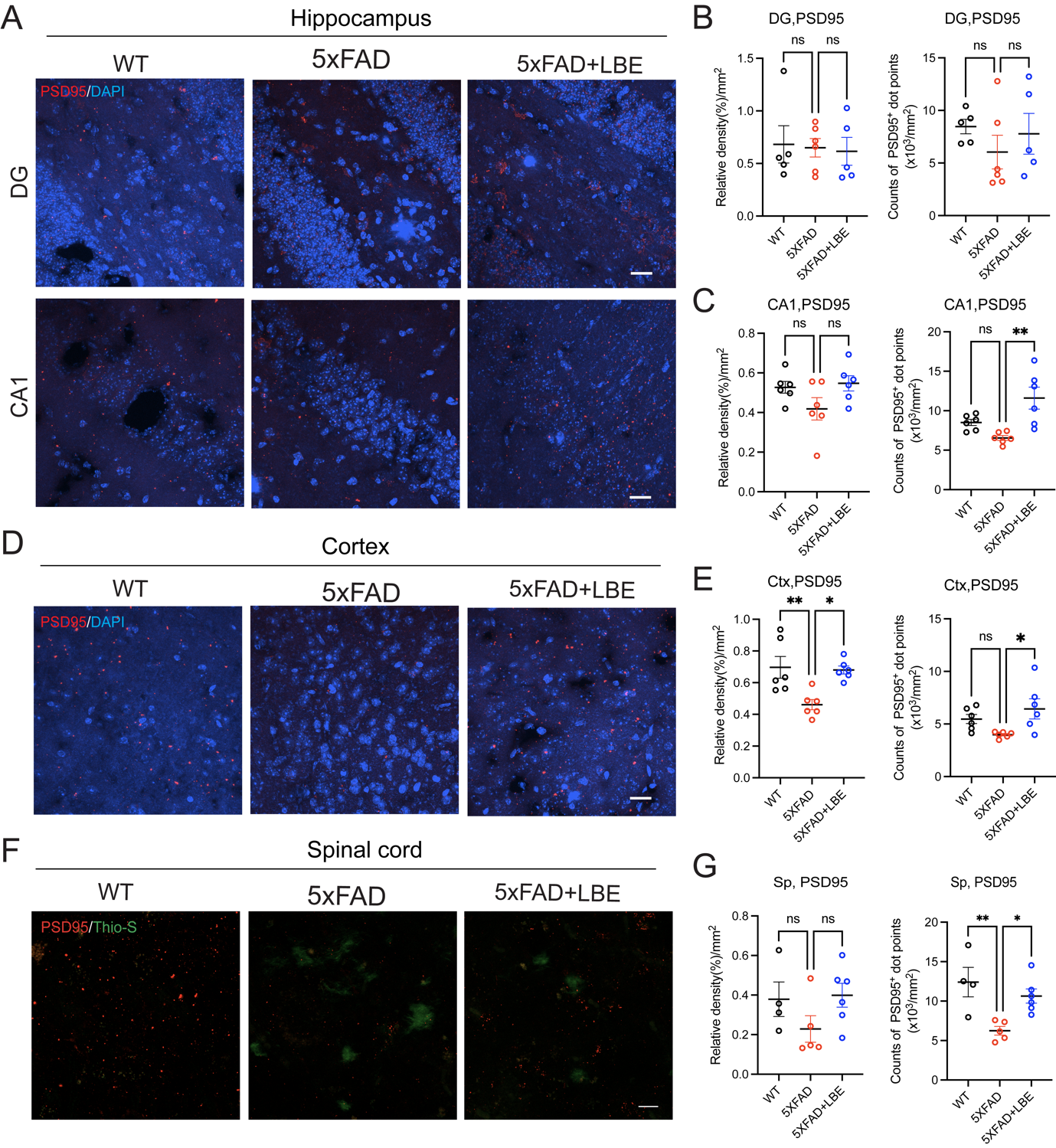


**SFigure 6. LBE treatment preserved post-synaptic density in the CNS of 5xFAD mice.** (A) Images of brain slices stained PSD95 (red) labeling presynaptic density from WT, 5xFAD and 5xFAD+LBE mice. (B) Quantification of the relative density and counts of PSD95-positive dot points in the DG region. (C) Quantification of the relative density and counts of PSD95-positive dot points in the CA1 region. Scale bar:20μm. (D) Images of brain slices stained PSD95 (red) labeling presynaptic density from WT, 5xFAD and 5xFAD+LBE mice. (E) Quantification of the relative density and counts of PSD95-positive dot points in the cortex region. (F) Images of spinal cord slices stained PSD95 (red) labeling presynaptic density from WT, 5xFAD and 5xFAD+LBE mice. Scale bar:20μm. (G) Quantification of the relative density, and counts of PSD95-positive dot points in the spinal cord. (H) Images of retina slices stained for PSD95 (red) labeling presynaptic density merged with DAPI from WT, 5xFAD and 5xFAD+LBE mice. (I) Quantification of the fluorescence intensity of PSD95-positive in the retina. Scale bar:20μm. Data was shown as mean ± SEM. *: p < 0.05; **: p < 0.01; ***: p < 0.001 by one-way ANOVA test with Tukey’s multiple comparison test.
