# Supplementary material for: Lycium barbarum Extract Enhanced Neuroplasticity and Functional Recovery in 5xFAD Mice via Modulating Microglial Status of the Central Nervous System
Source: CNS Neurosci Ther. 2024 Nov 20;30(11):e70123. doi: 10.1111/cns.70123 (PMC11576918; doi:10.1111/cns.70123)

Full unedited gel/blot for Figure X

For revised manuscript

Lycium barbarum extract enhanced neuroplasticity and functional recovery in 5xFAD mice via modulating microglial status of the central nervous system

Zhongqing Sun[1,2,3,4], Jinfeng Liu[3], Zihang Chen[5,6], Kwok-Fai So[3,7,8], Yong Hu[4,9*], Kin Chiu[3,5,7*]

*Corresponding Author:

Dr. Kin Chiu, State Key Lab of Brain and Cognitive Sciences, Department of Psychology, The University of Hong Kong, Room 409, Hong Kong Jockey Club Building for Interdisciplinary Research, 5 Sassoon Road, Pokfulam, Hong Kong SAR, P.R. China.

E-mail: datwai@hku.hk.

Dr. Yong Hu, Department of Orthopaedics & Traumatology, School of Clinical Medicine, Li Kai Shing Faculty of Medicine, The University of Hong Kong, Hong Kong SAR, P.R. China.

Email: yhud@hku.hk.

Tel.: (852) 2974-0336; Fax: (852) 2974-0335.

Figure4A-Hippocampus

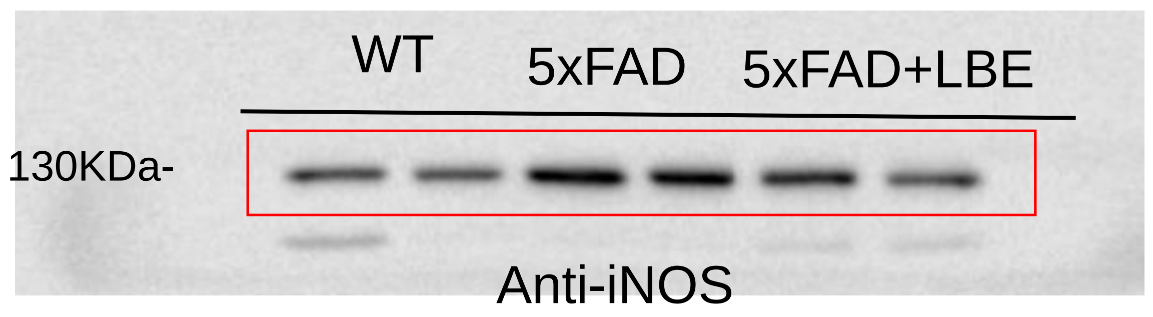

Figure4B	WT	5xFAD	LBE+5xFAD
iNOS/GAPDH	0.839344	1.955632	0.797447
	1.050949	1.488758	0.85453
	0.670829	1.136084	0.594614
	1.526973	1.077406	0.759445
Mean	1.022	1.414	0.7515
SEM	0.1854	0.202	0.05583

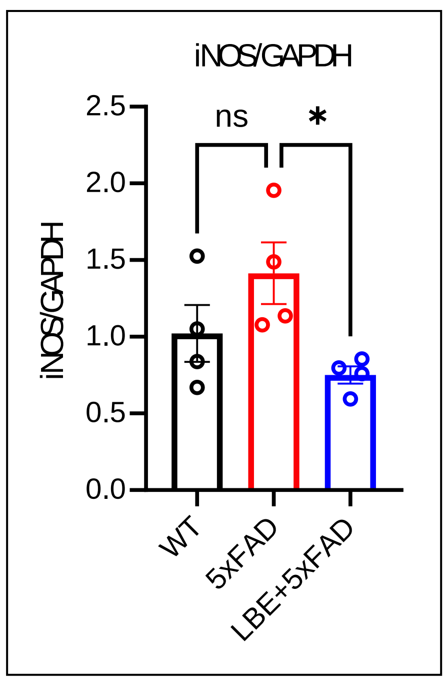

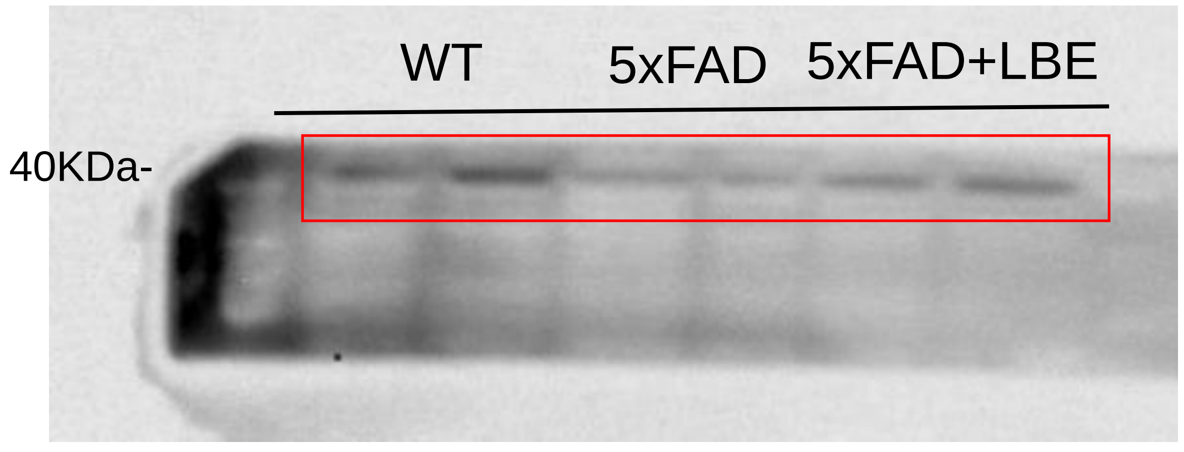

Anti-Arg-1

Figure4B	WT	5xFAD	LBE+5xFAD
Arg-1/GAPDH	0.792363	0.531346	0.972946
	1.036974	0.541497	1.124869
	0.472738	0.548861	0.731049
	0.487606	0.609542	1.159443
Mean	0.6974	0.5578	0.9971
SEM	0.135	0.01761	0.09749

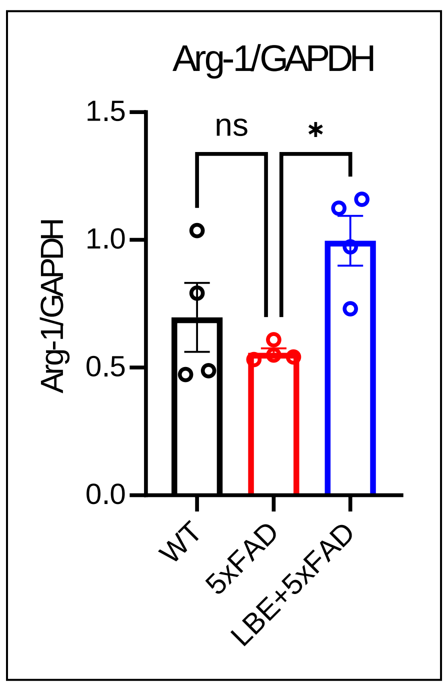

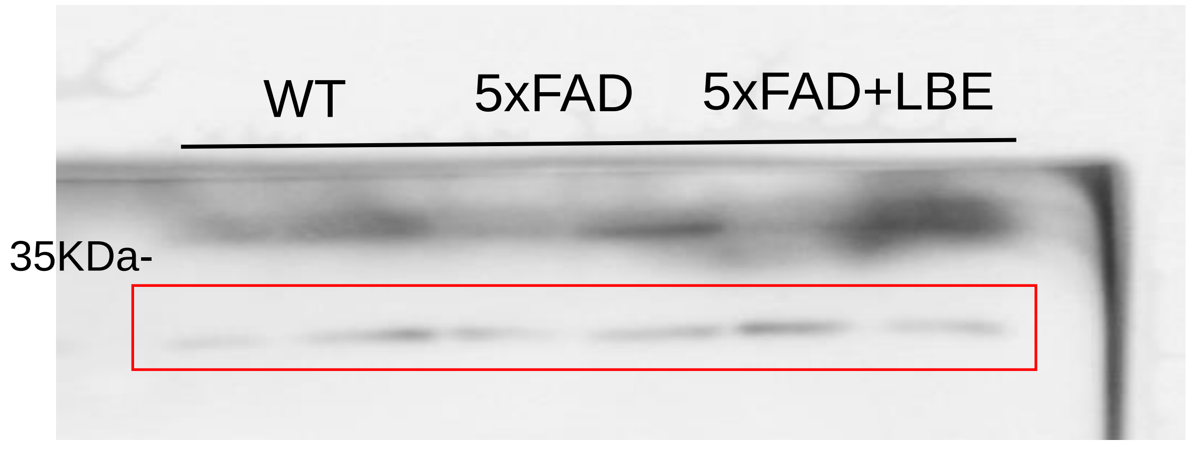

Anti-IL-1β

Figure4B	WT	5xFAD	LBE+5xFAD
IL-1β/GAPDH	0.768651	0.600479	0.894992
	0.980225	1.399013	0.616515
	0.546132	0.501743	0.602151
	1.168288	0.861517	0.279456
Mean	0.8658	0.8407	0.5983
SEM	0.1342	0.201	0.1258

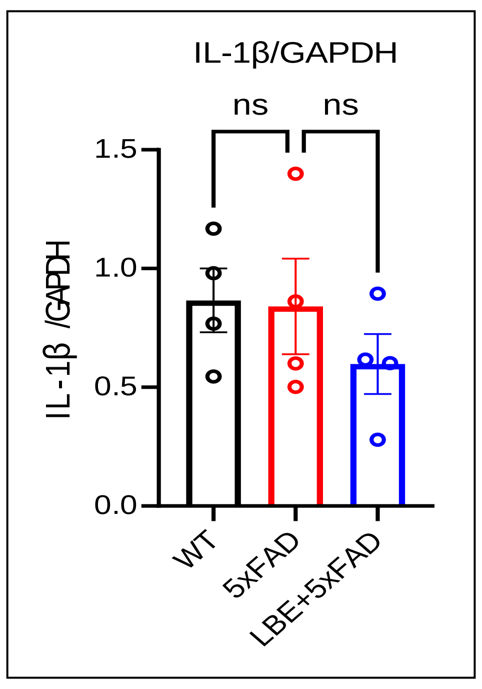

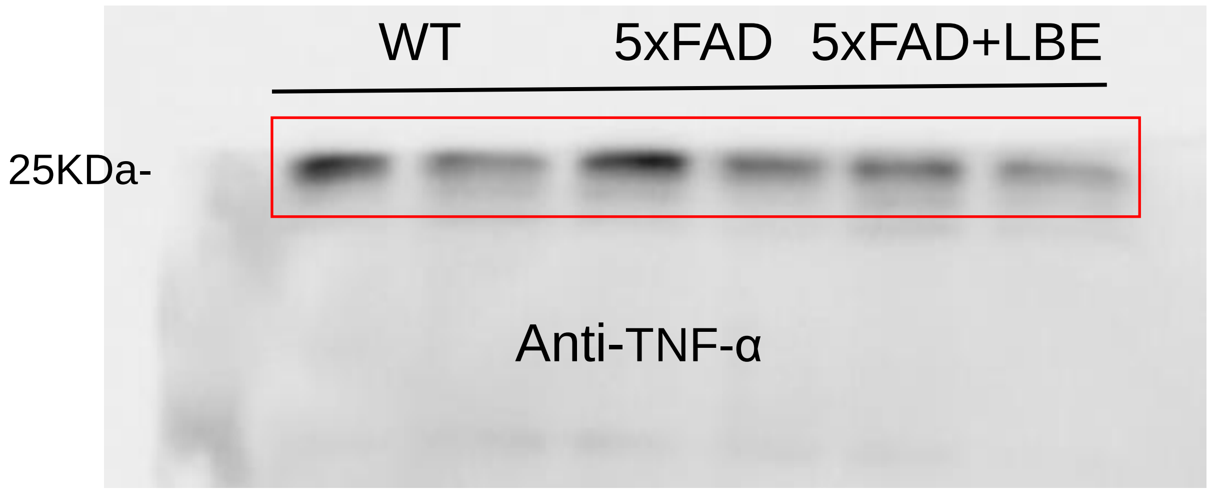

Figure4B	WT	5xFAD	LBE+5xFAD
TNF-α/GAPDH	0.444895	1.132156	1.049323
	0.94891	1.273587	0.376601
	1.566848	1.831622	0.673663
	1.212278	1.571208	0.820515
Mean	1.043	1.452	0.73
SEM	0.2362	0.1561	0.1409

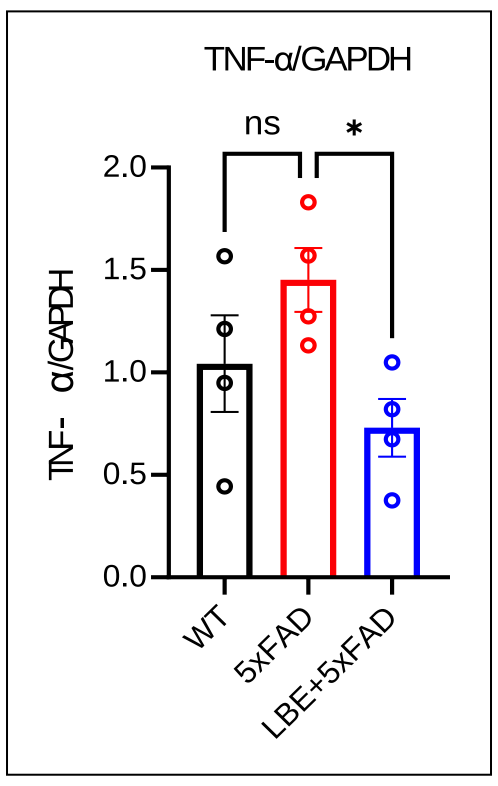

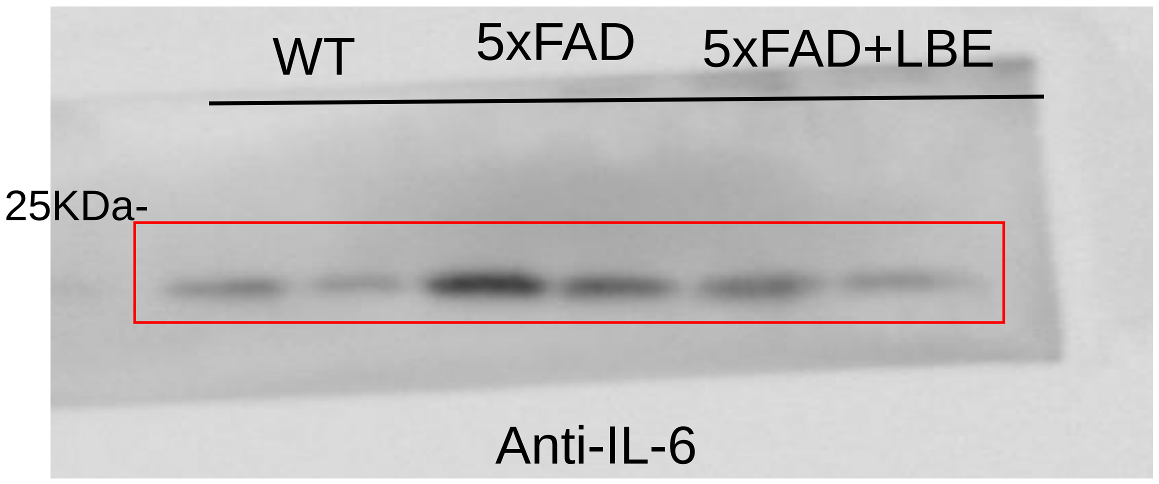

Figure4B	WT	5xFAD	LBE+5xFAD
IL-6/GAPDH	0.949965	0.809569	0.592781
	0.987791	0.816133	0.247606
	0.284662	2.149908	1.480476
	0.563755	2.634109	1.098277
Mean	0.6965	1.602	0.8548
SEM	0.1674	0.4665	0.272

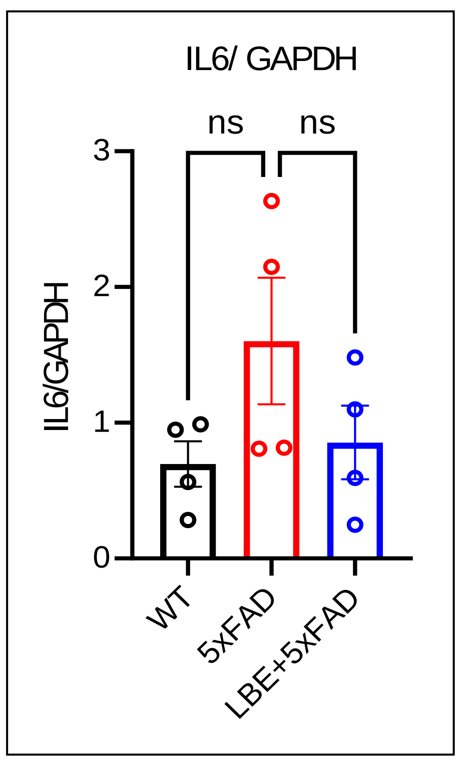

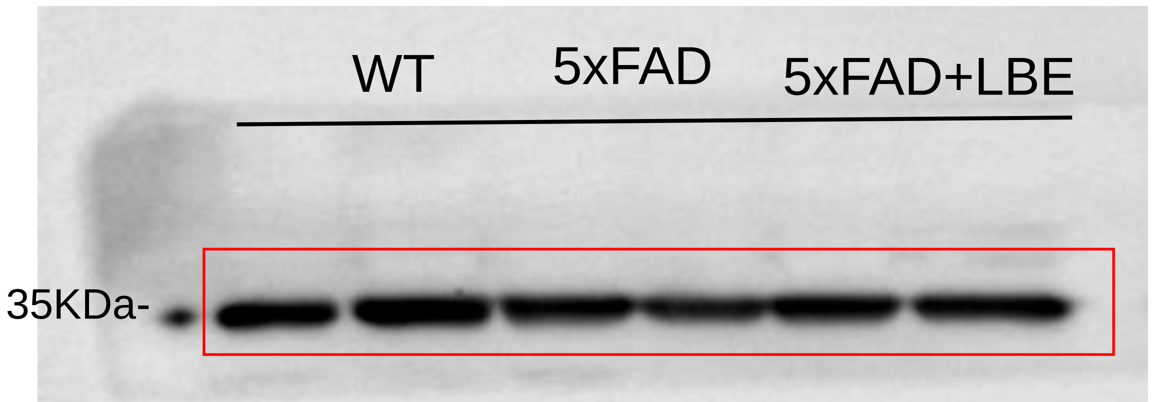

Anti-GAPDH

Figure4C-Cortex

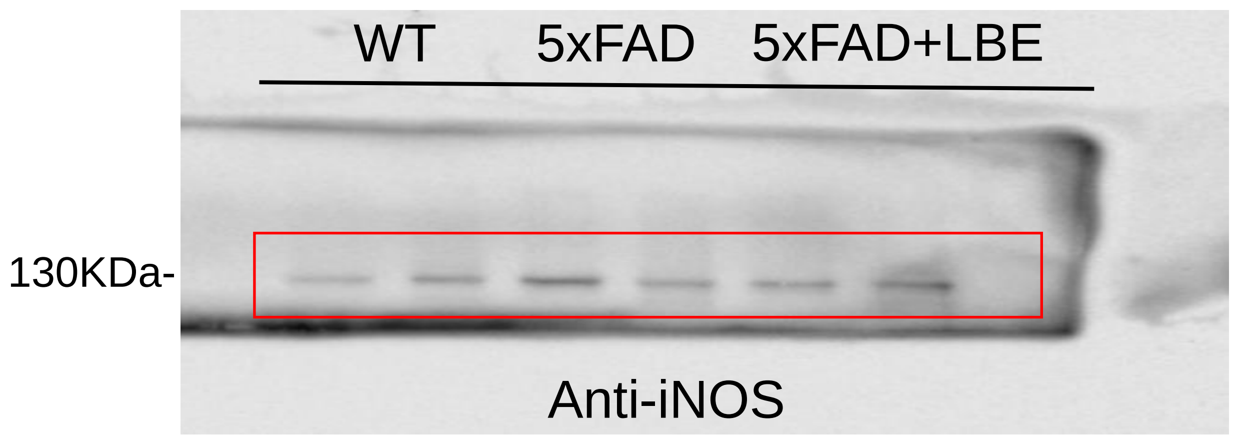

Figure4D	WT	5xFAD	LBE+5xFAD
iNOS/GAPDH	0.730675	1.477237	0.820577
	1.161871	1.205023	1.496033
	1.029649	1.971602	1.671423
	1.196142	1.524439	0.559092
Mean	1.03	1.545	1.137
SEM	0.1059	0.1588	0.2659

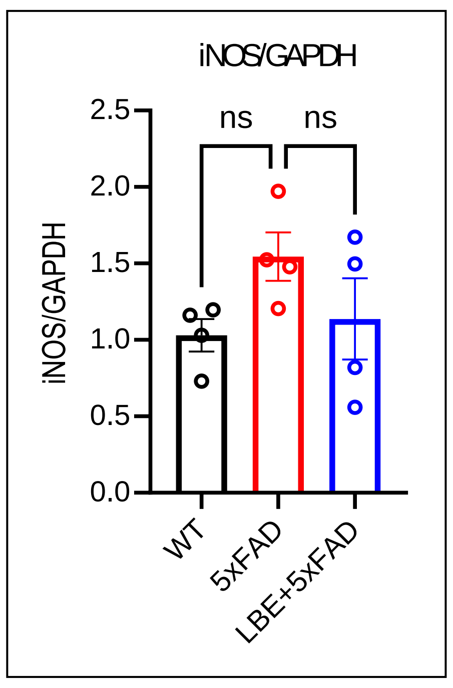

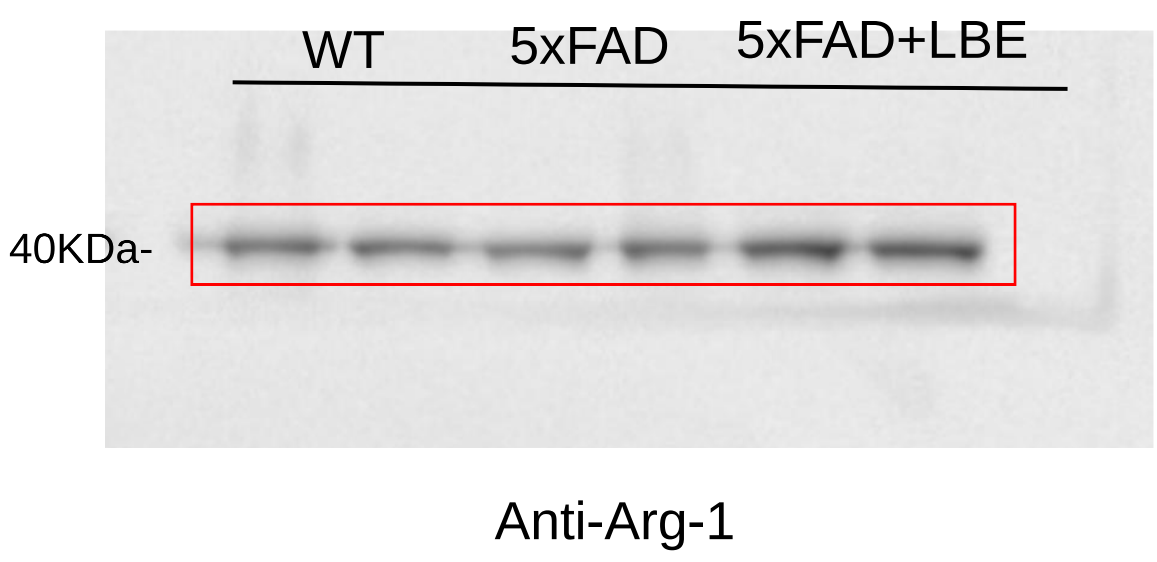

Figure4C	WT	5xFAD	LBE+5xFAD
Arg-1/GAPDH	1.243754	0.612576	1.234852
	0.660976	0.695687	1.479232
	1.178536	0.506501	1.278574
	0.947327	0.724628	0.918671
Mean	1.008	0.6348	1.228
SEM	0.1319	0.04893	0.116

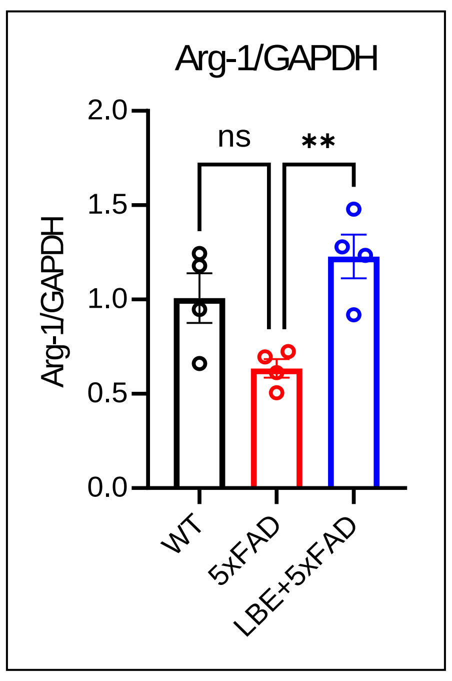

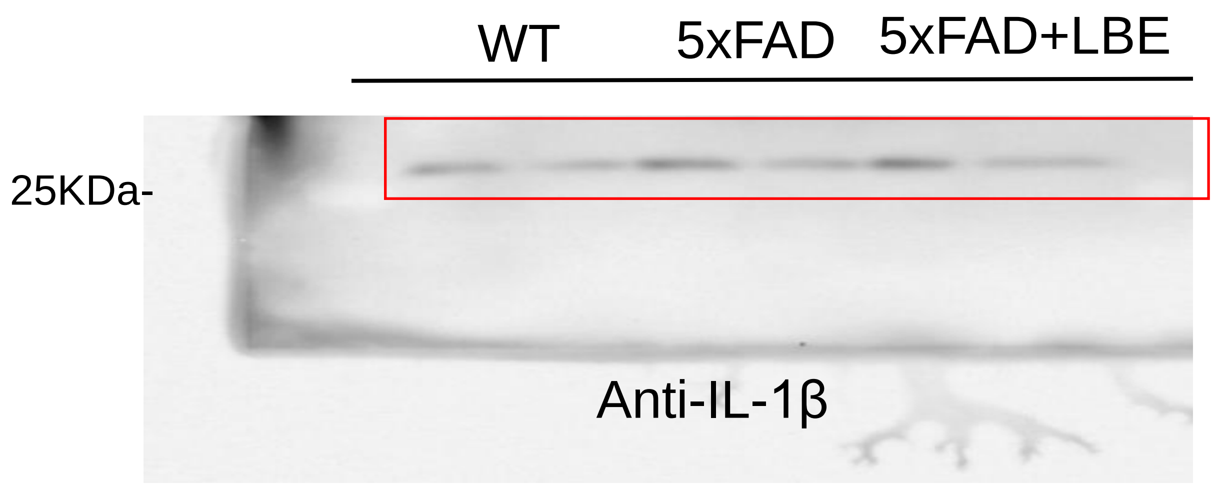

Figure4D	WT	5xFAD	LBE+5xFAD
IL-1β/GAPDH	1.00798	1.687794	2.319024
	1.112911	2.894843	0.772959
	0.523231	0.594247	1.314398
	1.418795	1.060936	0.617084
Mean	1.016	1.559	1.256
SEM	0.1859	0.4983	0.3846

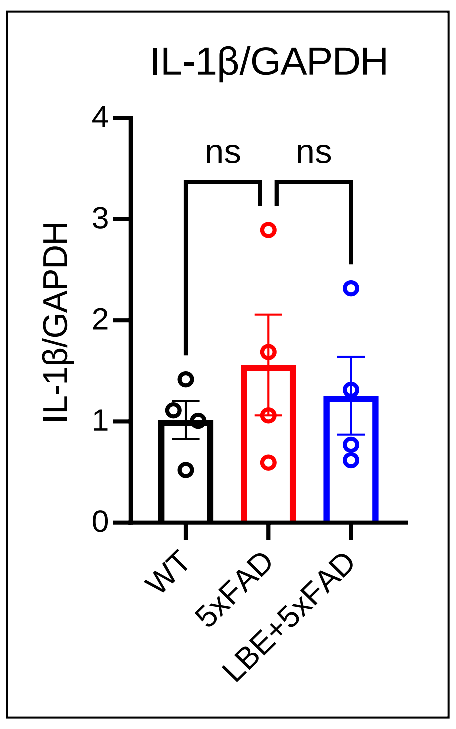

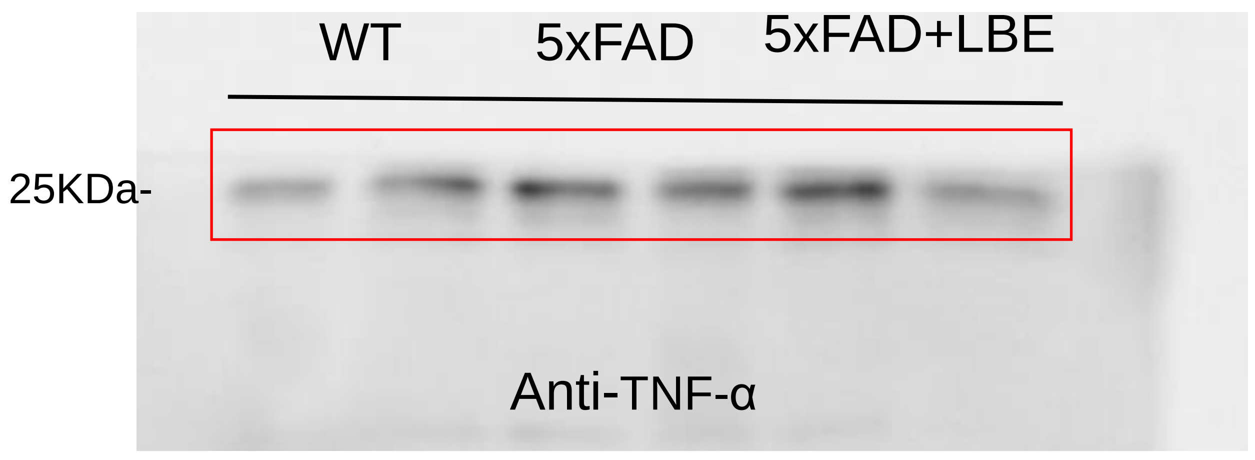

Figure4C	WT	5xFAD	LBE+5xFAD
TNF-α/GAPDH	1.651037	1.47831	0.904139
	0.877154	1.91172	0.342494
	0.850444	1.878675	1.751719
	0.711222	1.432172	0.68421
Mean	1.022	1.675	0.9206
SEM	0.2127	0.1275	0.3002

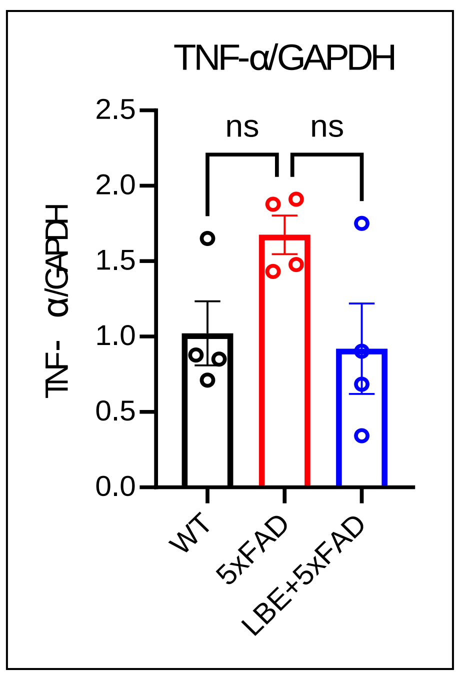

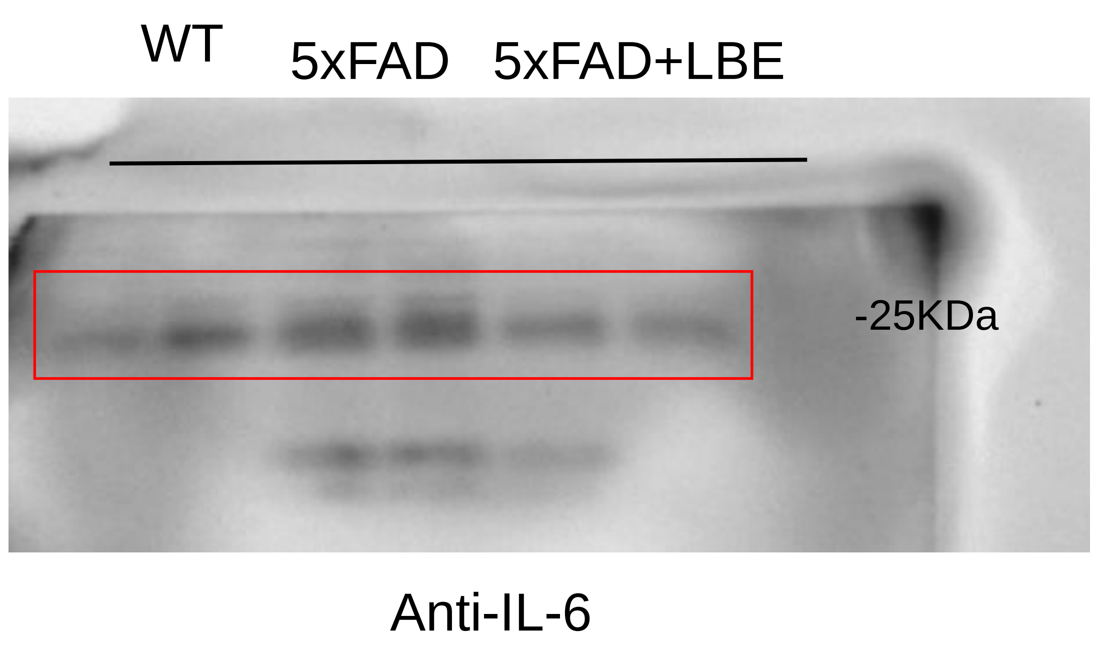

Figure4D	WT	5xFAD	LBE+5xFAD
IL-6/GAPDH	0.909839	2.25525	1.4416979
	0.662158	1.964215	0.5967942
	0.855827	1.685727	1.0863392
	0.697933	1.293346	0.5316246
Mean	0.7814	1.8	0.9141
SEM	0.06001	0.2049	0.2151

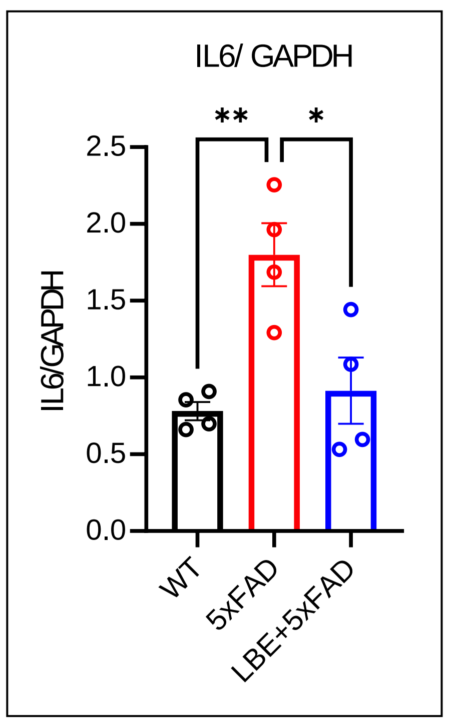

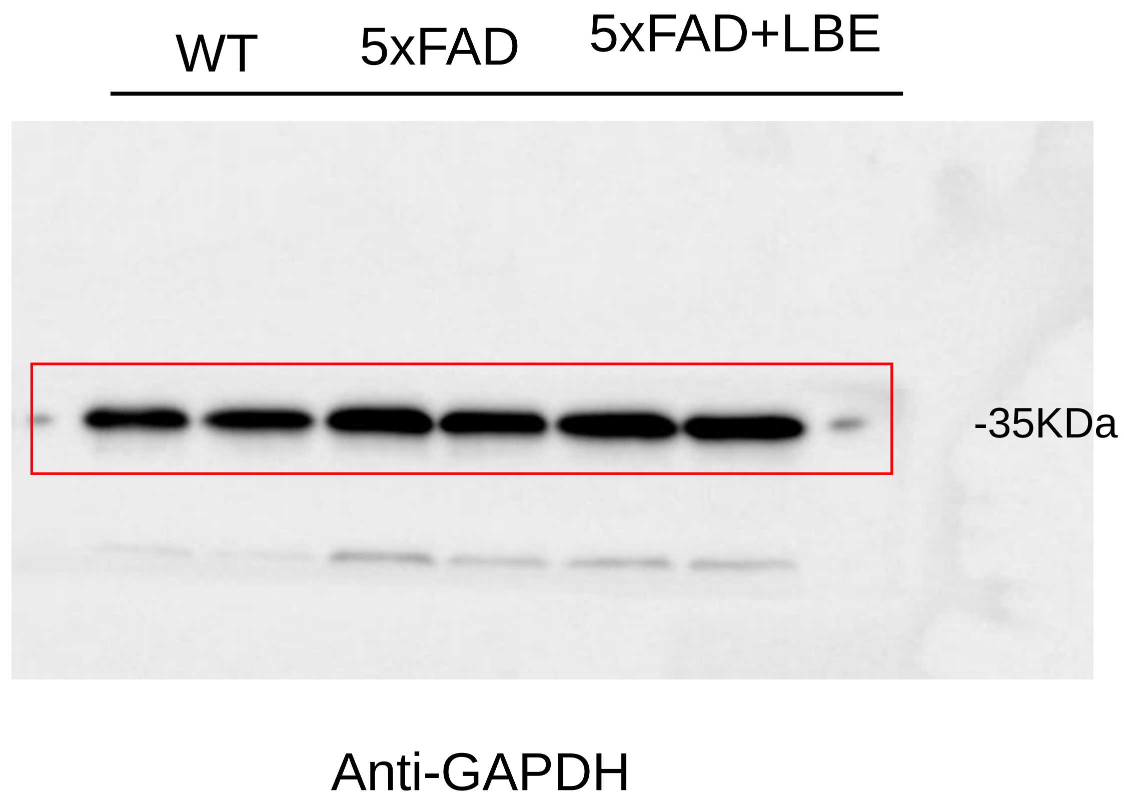

Figure4E-Spinal Cord

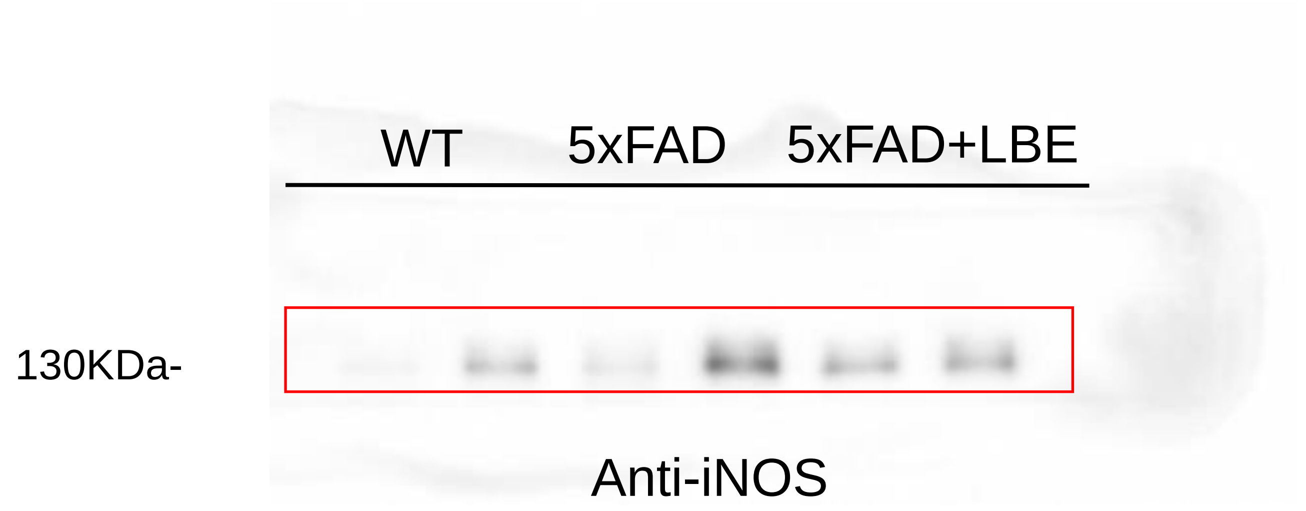

Figure4E	WT	5xFAD	LBE+5xFAD
	0.706758	1.092522	0.835283
iNOS/GAPDH	1.327111	1.96466	0.971369
	0.338774	1.231779	0.978782
	0.829889	1.801044	1.016976
Mean	0.8006	1.523	0.9506
SEM	0.2042	0.2126	0.03972

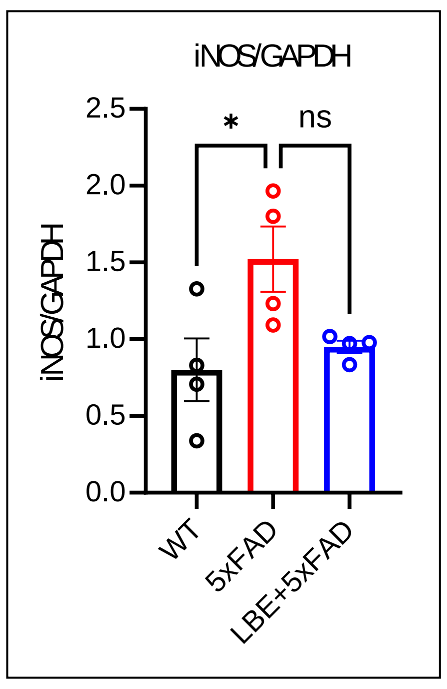

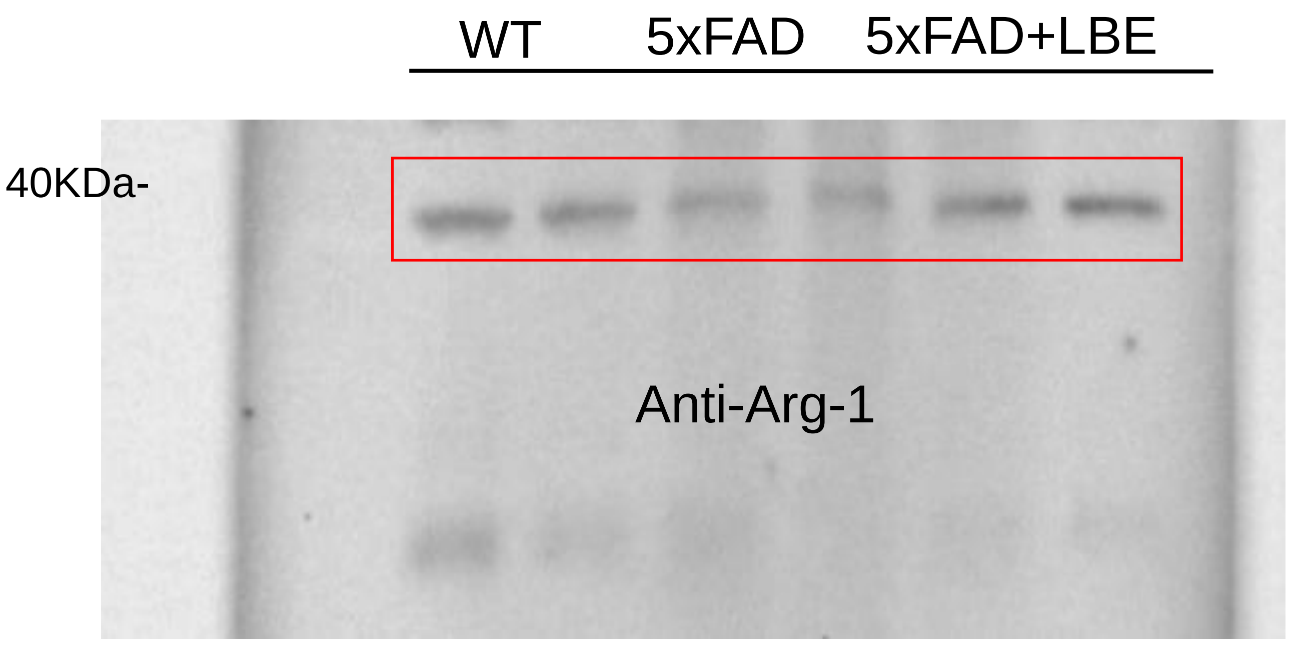

Figure4F	WT	5xFAD	LBE+5xFAD
Arg-1/GAPDH	0.680903	0.314227	0.888073
	0.76511	0.610876	0.875864
	0.427081	0.484521	0.75488
	0.457669	0.494694	0.617627
Mean	0.5827	0.4761	0.7841
SEM	0.08305	0.06109	0.06311

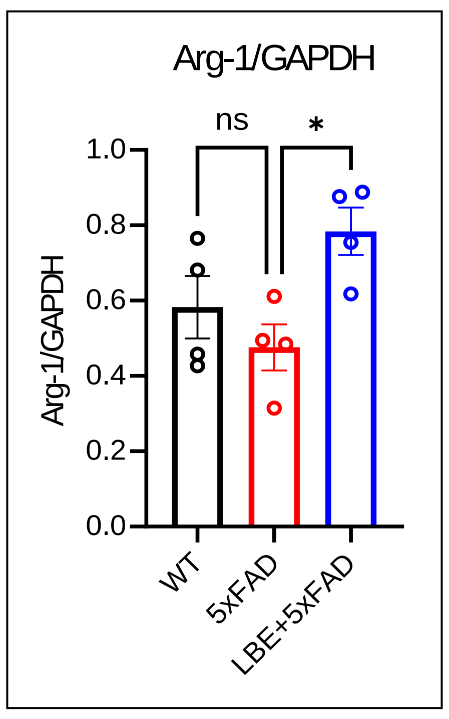

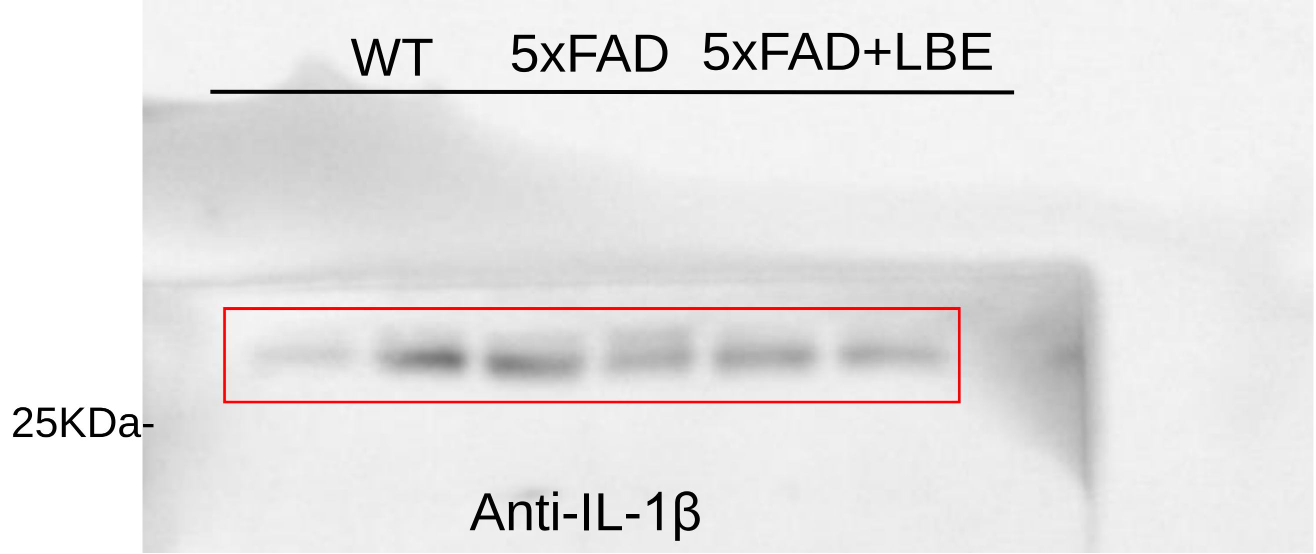

Figure4F	WT	5xFAD	LBE+5xFAD
IL-1β/GAPDH	0.310322	0.821998	0.560867
	0.812473	0.59443	0.336776
	0.629876	0.568171	0.487593
	0.568835	0.529778	0.483608
Mean	0.5804	0.6286	0.4672
SEM	0.1038	0.06582	0.04697

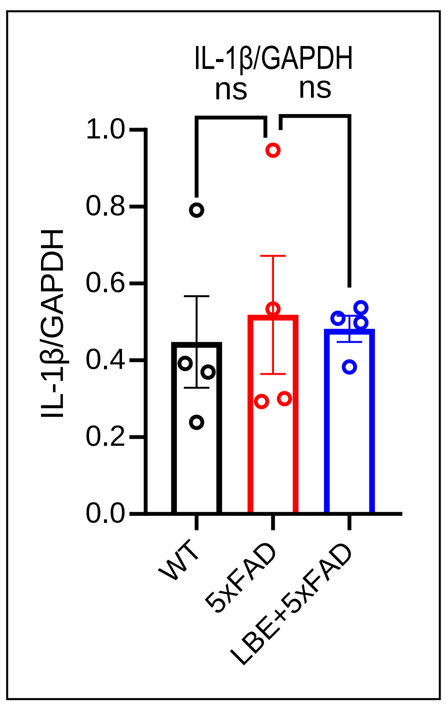

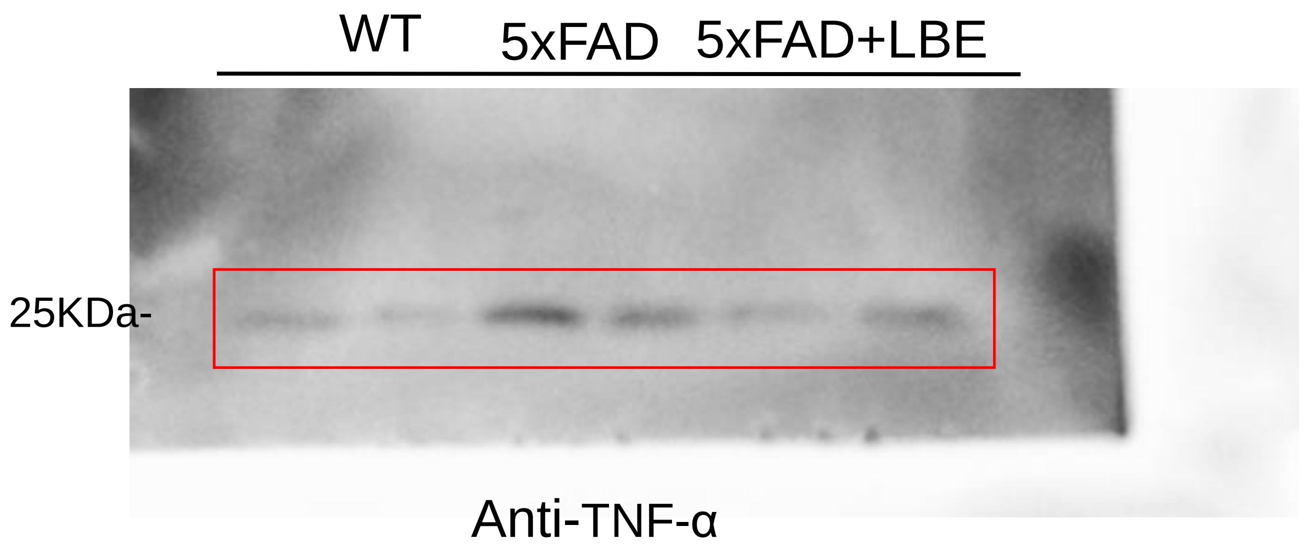

Figure4F	WT	5xFAD	LBE+5xFAD
TNF-α/GAPDH	1.059061	1.89957	1.111457
	0.828636	2.046819	1.23178
	1.099874	1.757911	0.864766
	0.543758	1.361908	0.645184
Mean	0.8828	1.767	0.9633
SEM	0.1278	0.1472	0.1307

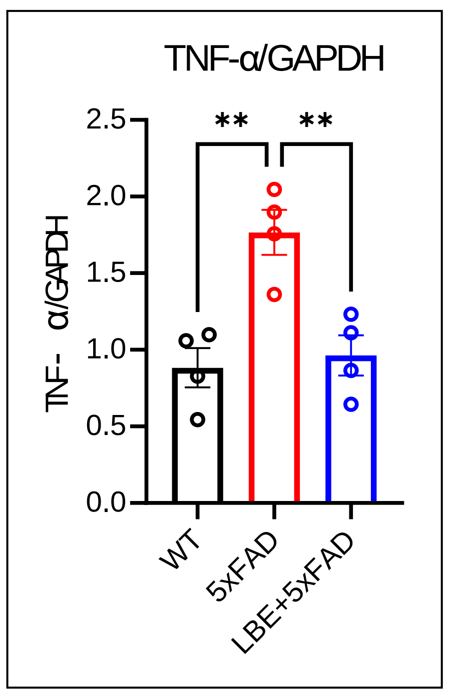

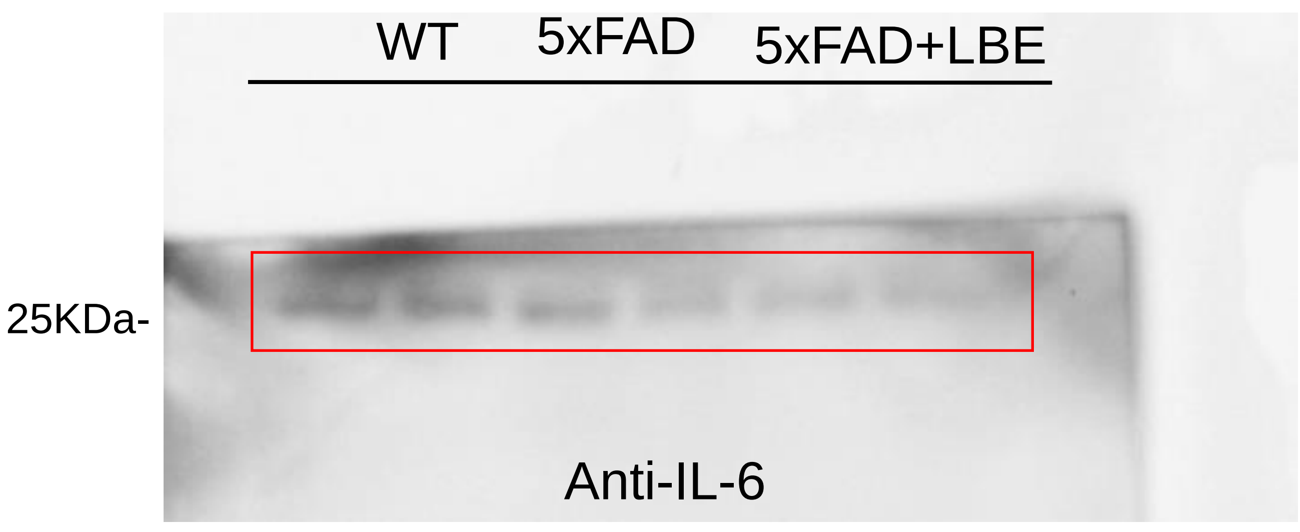

Figure4F	WT	5xFAD	LBE+5xFAD
IL-6/GAPDH	0.933312	0.915307	0.935233
	0.790837	0.834731	0.533628
	0.857821	1.067058	0.809023
	0.809224	0.7815	0.408487
Mean	0.8478	0.8996	0.6716
SEM	0.03181	0.06221	0.1213

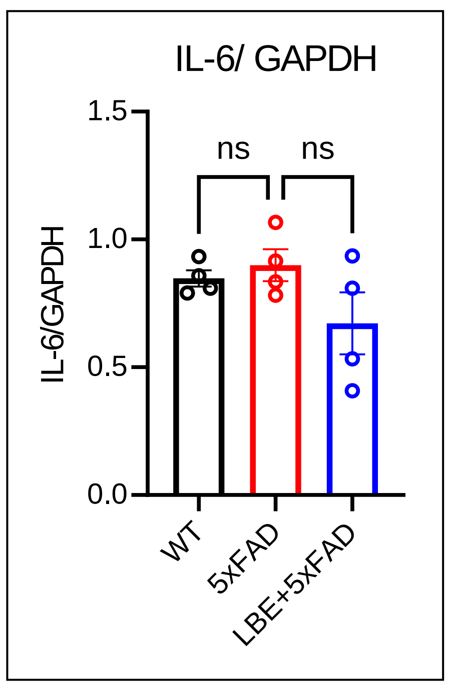

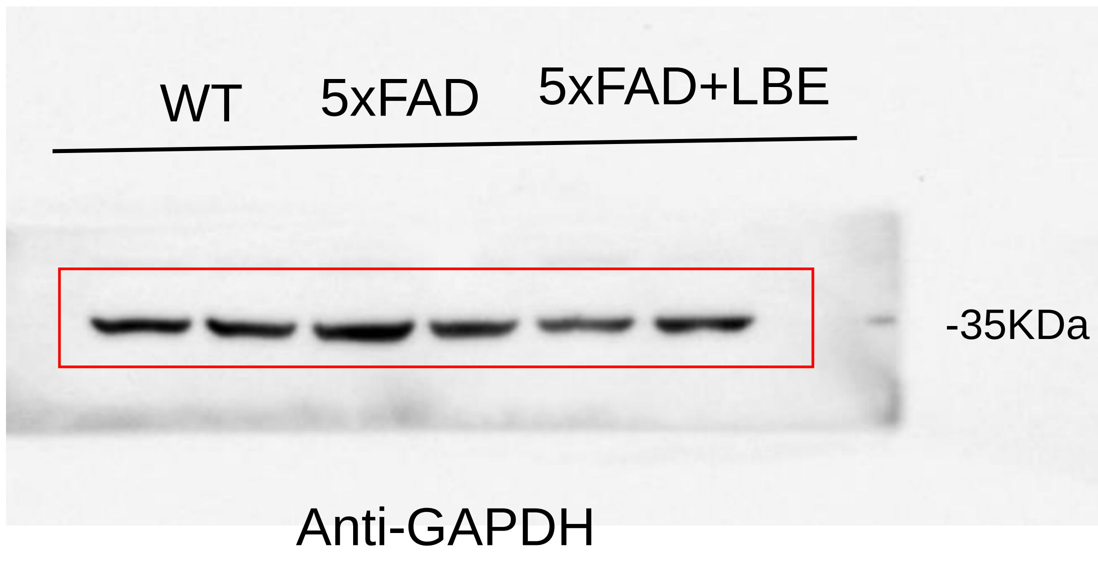

Figure 4G-Retina

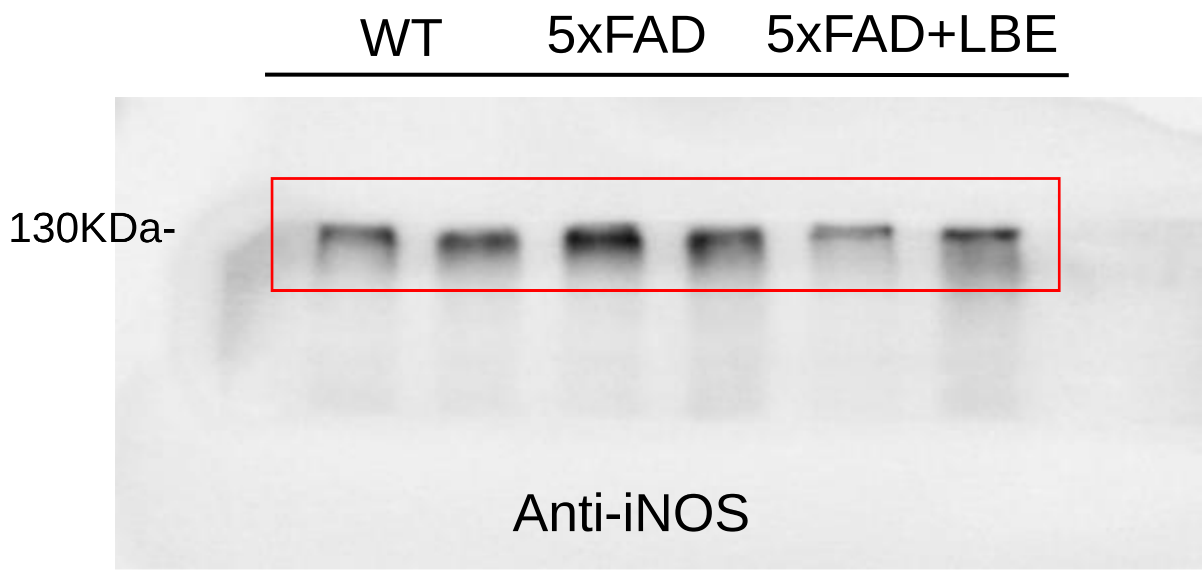

Figure4G	WT	5xFAD	LBE+5xFAD
	1.023938	0.882436	0.378613
iNOS/GAPDH	0.92846	1.698851	0.320776
	0.587923	0.722033	0.715886
	0.694593	1.425957	0.622248
Mean	0.8087	1.182	0.5094
SEM	0.101	0.2288	0.09489

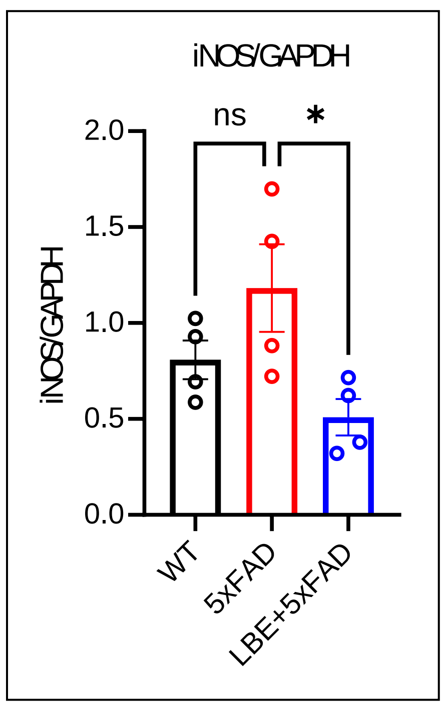

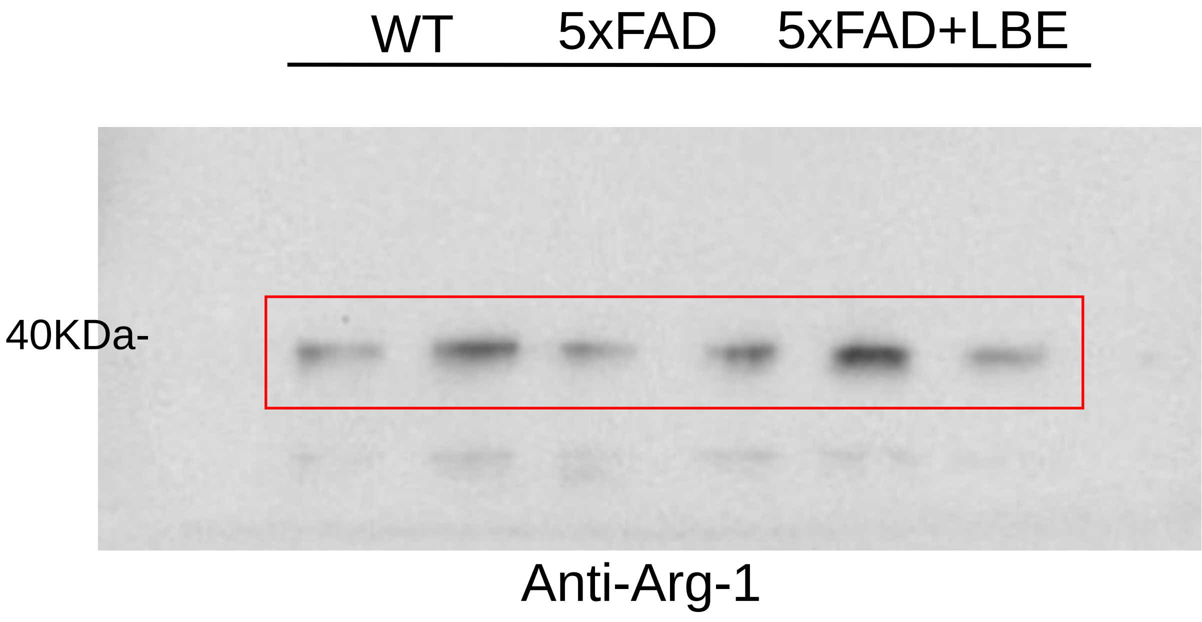

Figure4G	WT	5xFAD	LBE+5xFAD
Arg-1/GAPDH	0.406823	0.689223	0.571076
	0.905818	0.545389	0.985674
	0.451397	0.666308	0.859796
	0.3674	0.46226	0.956424
Mean	0.5329	0.5908	0.8432
SEM	0.1255	0.05321	0.09462

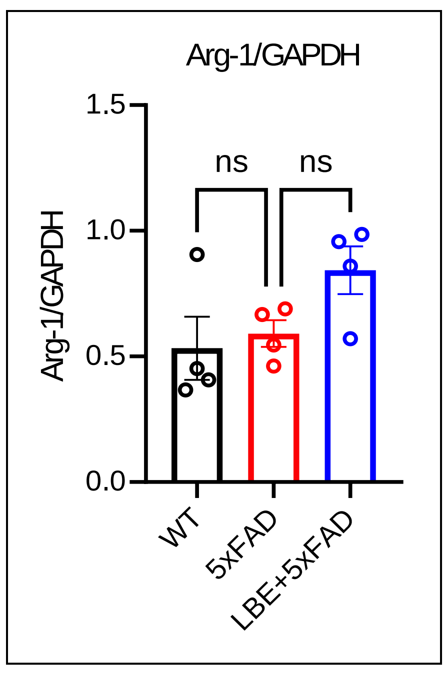

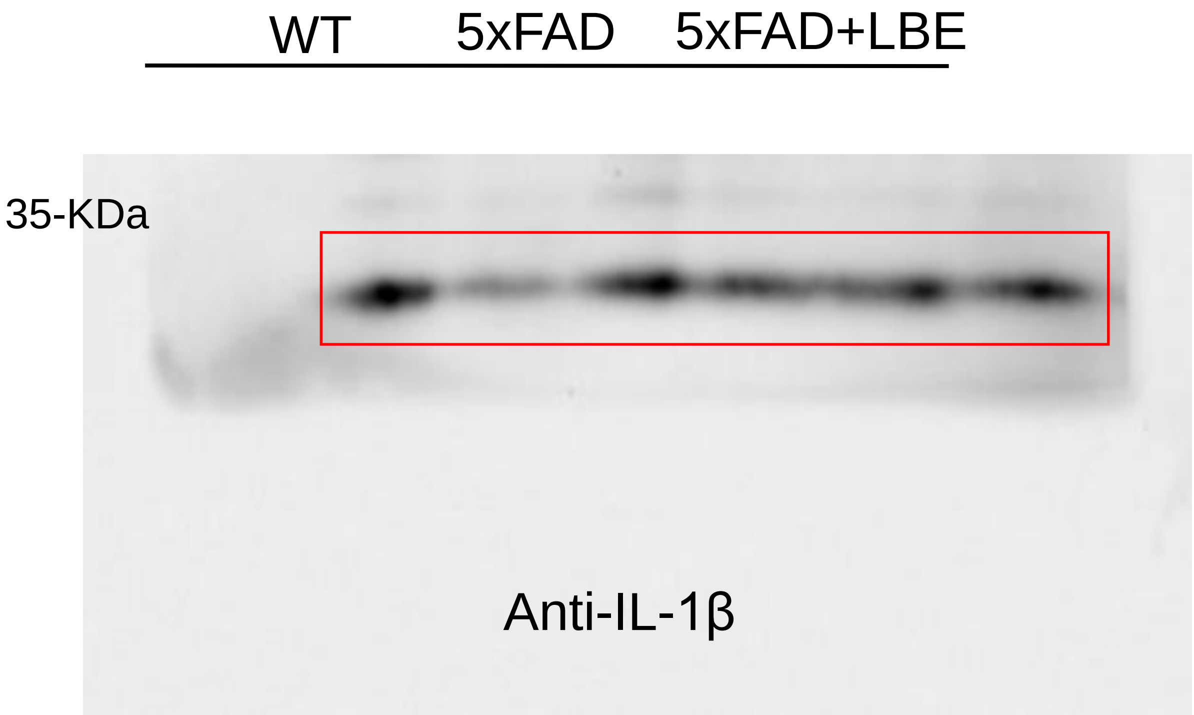

Figure4G	WT	5xFAD	LBE+5xFAD
IL-1β/GAPDH	0.23863	0.29268	0.537151
	0.391795	0.533907	0.509422
	0.329732	0.350067	0.421619
	0.740986	0.518311	0.398788
Mean	0.4253	0.4237	0.4667
SEM	0.1098	0.06034	0.03346

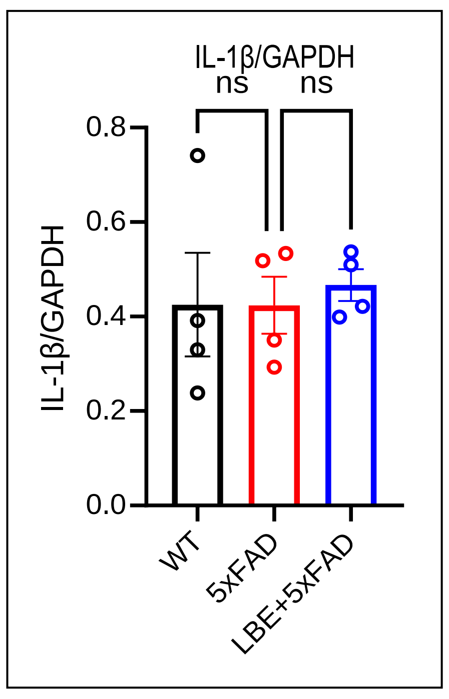

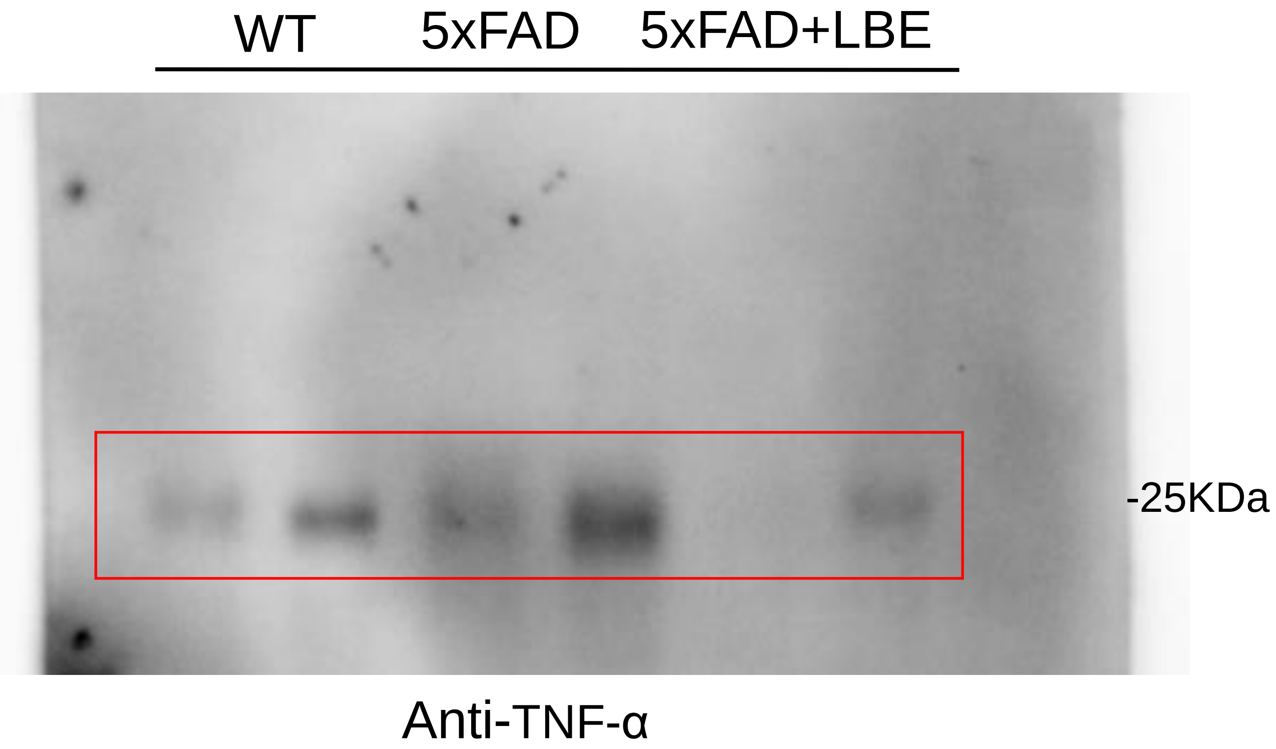

Figure4G	WT	5xFAD	LBE+5xFAD
TNF-α/GAPDH	0.75613	1.482174	0.56767
	1.695095	1.917525	0.875061
	0.817273	1.372217	0.990182
	0.981142	1.350716	0.984342
Mean	1.062	1.531	0.8543
SEM	0.2162	0.1321	0.09915

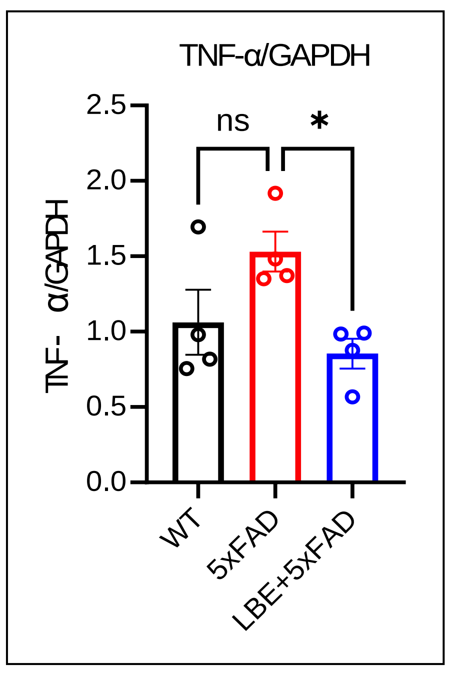

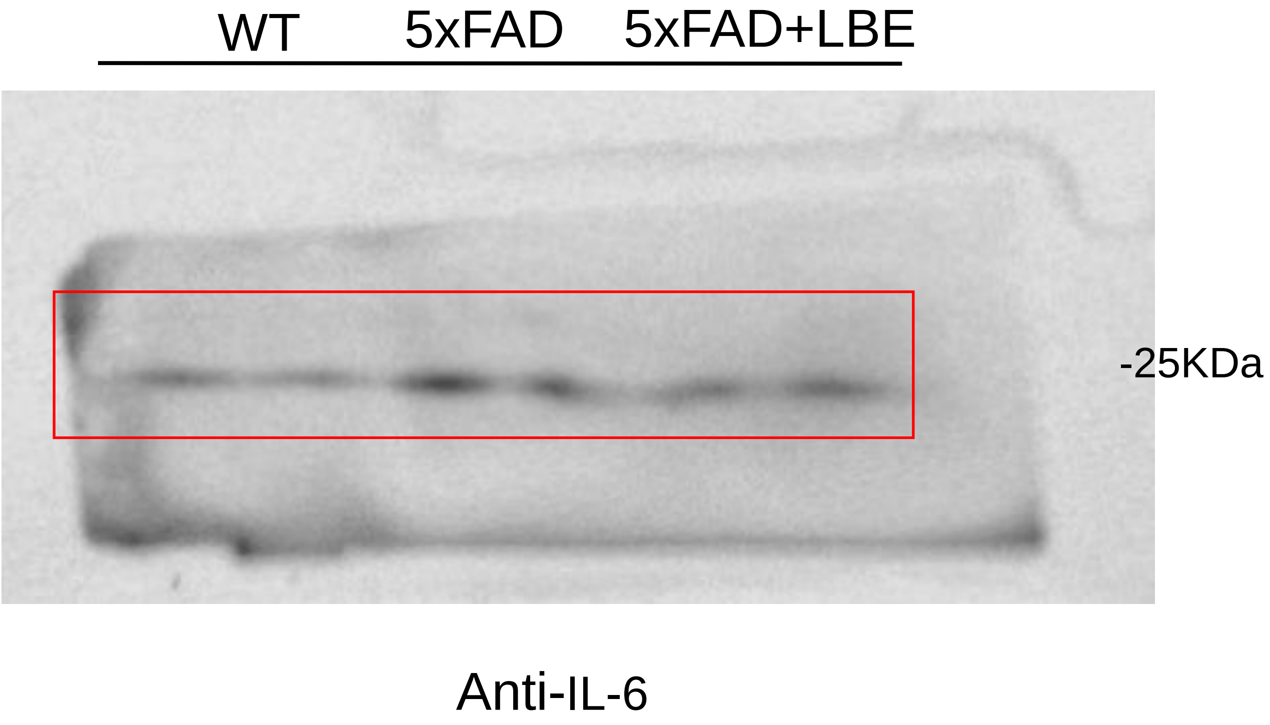

Figure4G	WT	5xFAD	LBE+5xFAD
IL-6/GAPDH	0.84899	1.117712	0.474284
	1.334445	1.406051	0.699717
	0.648065	1.327081	0.812076
	0.754539	1.26809	0.946935
Mean	0.8965	1.28	0.7333
SEM	0.1516	0.06095	0.1

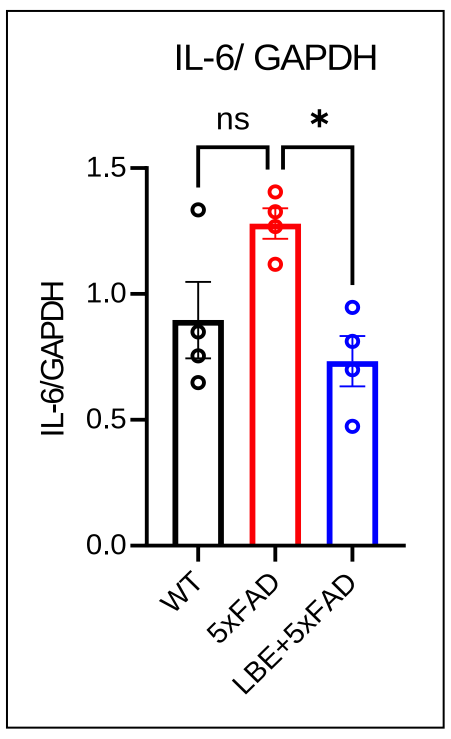

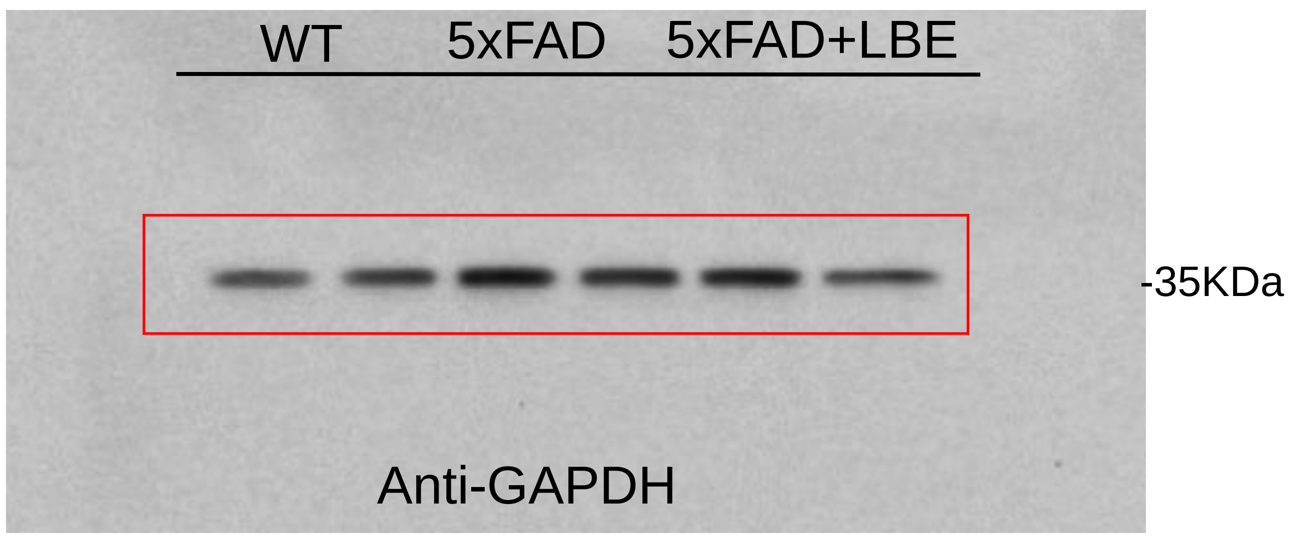

Figure8A-hippocampus

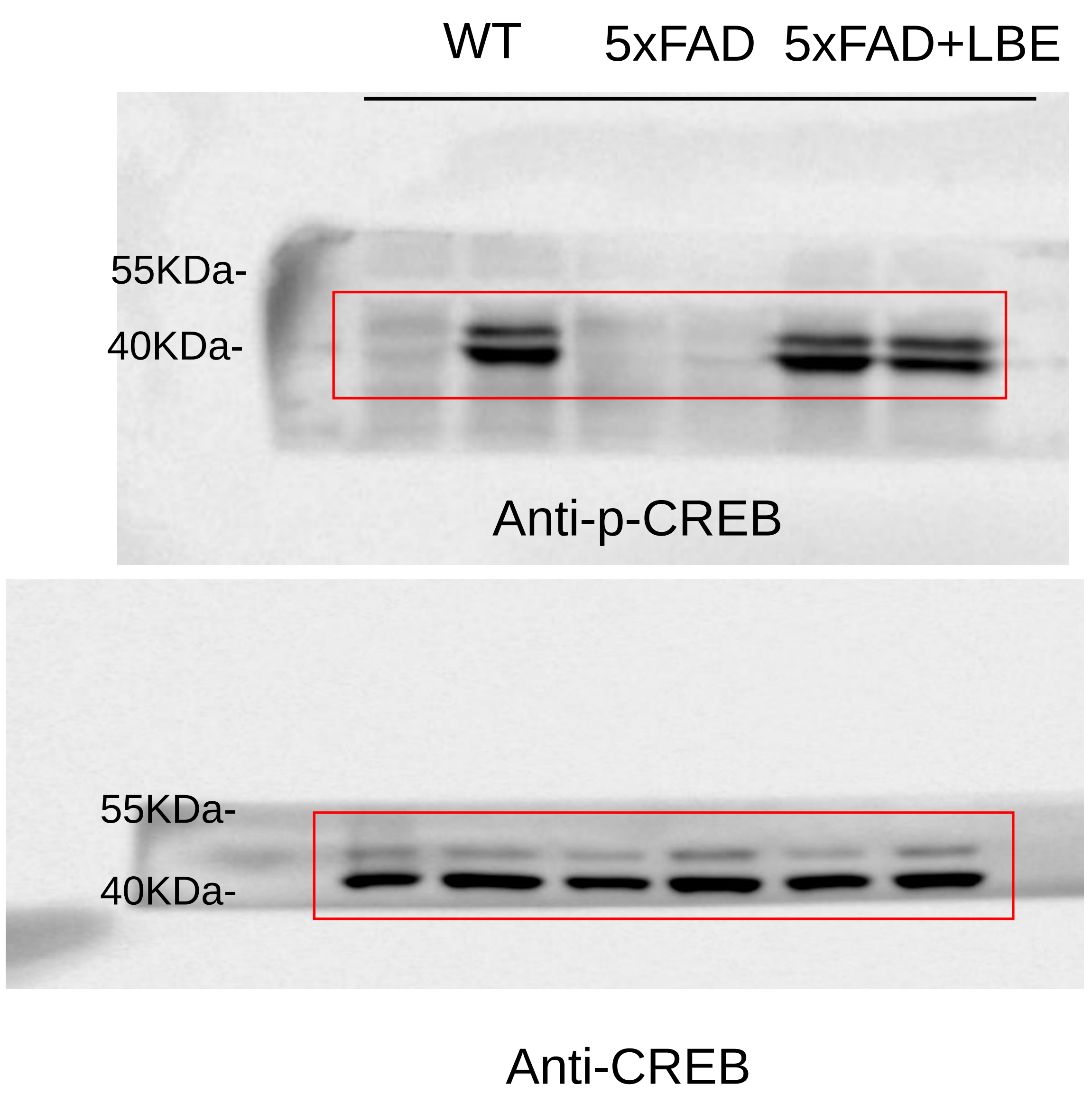

Figure8B	WT	5xFAD	LBE+5xFAD
	0.5876851	0.5016324	1.6822744
pCREB/CREB	0.8441431	0.6884563	2.1241161
	1.0146666	0.6017432	4.1157933
	3.6439555	0.4568409	3.9839025
Mean	1.523	0.5622	2.977
SEM	0.7125	0.05186	0.6268

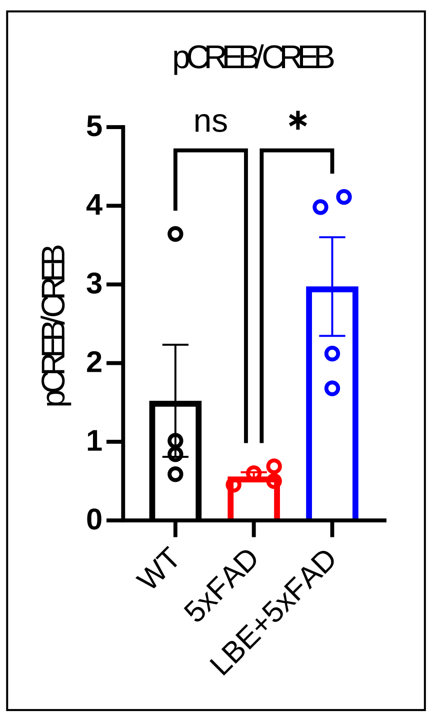

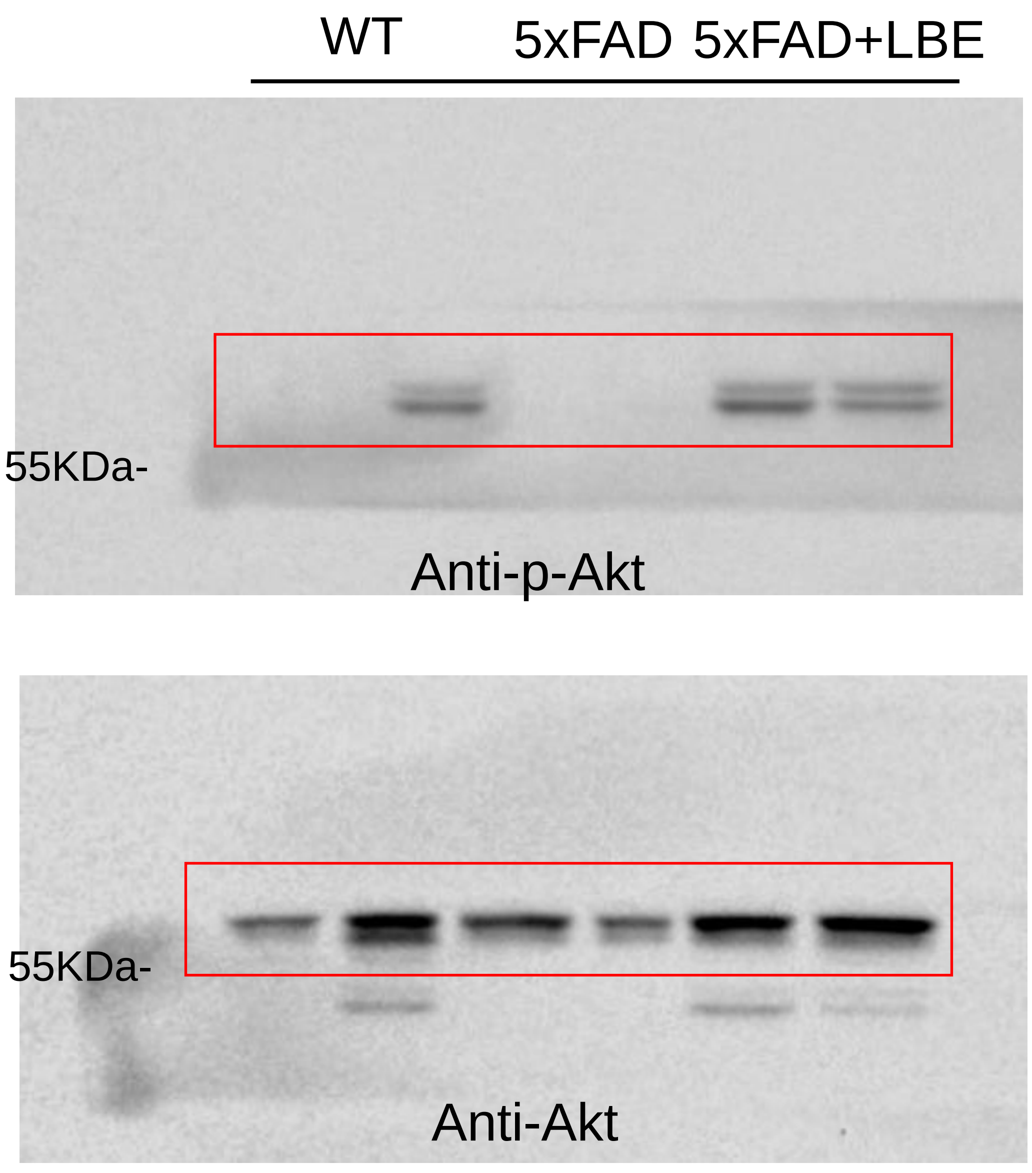

Figure8B	WT	5xFAD	LBE+5xFAD
	0.3471975	0.1823872	1.5552137
pAKT/AKT	0.9174065	0.2835715	1.0318063
	0.9124108	0.7642753	0.9416503
	1.2294576	0.4810908	1.5404397
Mean	0.8516	0.4278	1.267
SEM	0.1838	0.1282	0.163

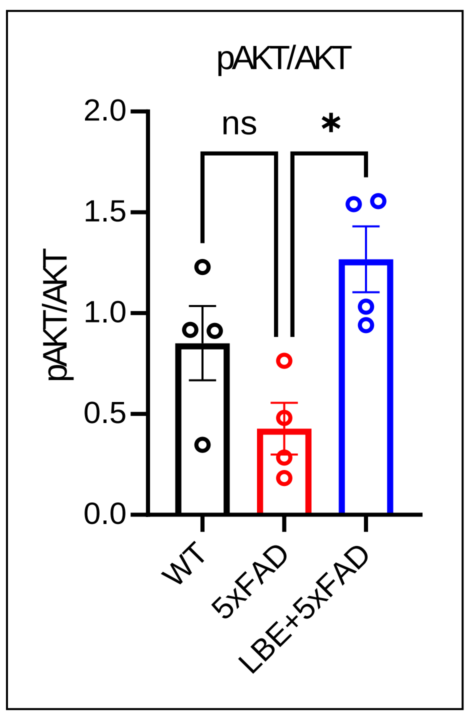

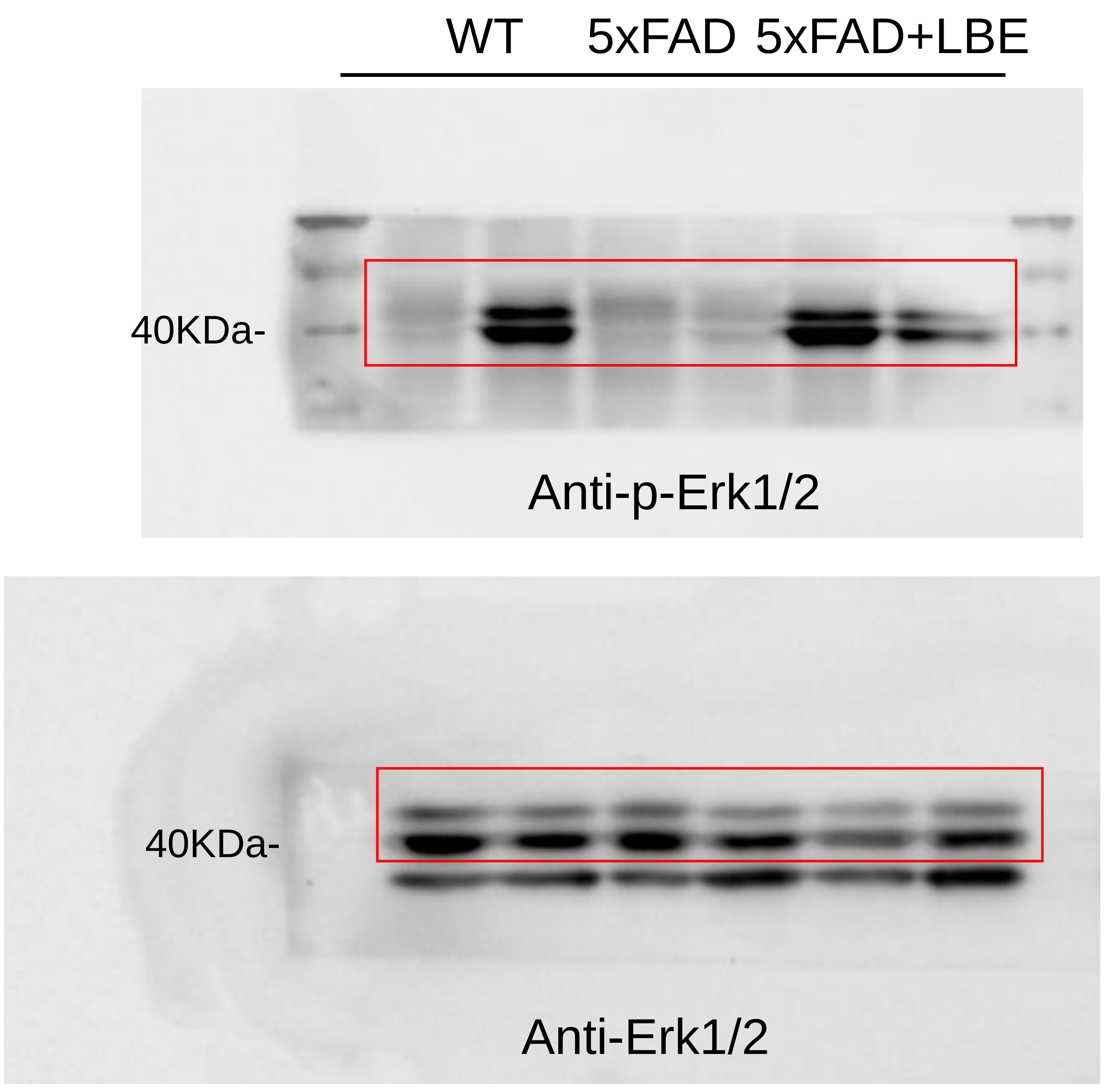

Figure8B	WT	5xFAD	LBE+5xFAD
	1.2019829	1.4119001	2.495803
pErk/Erk	1.2294576	0.4810908	1.3404397
	0.611362	0.4545912	2.3811537
	1.7360773	0.4059469	1.1792617
Mean	1.195	0.6884	1.849
SEM	0.23	0.2417	0.3426

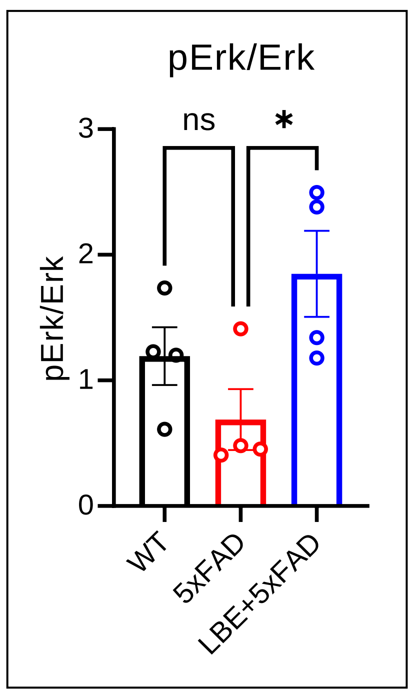

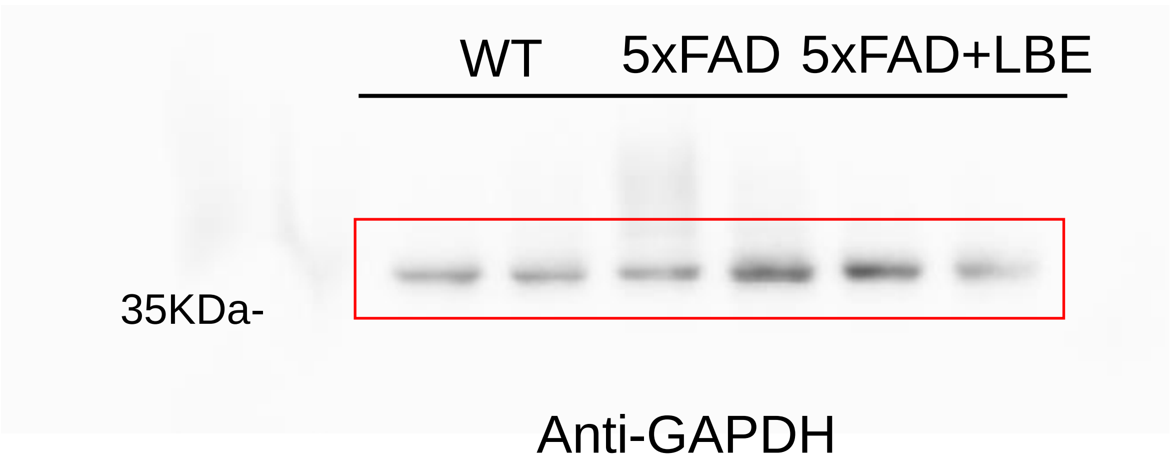

Figure8C-Cortex

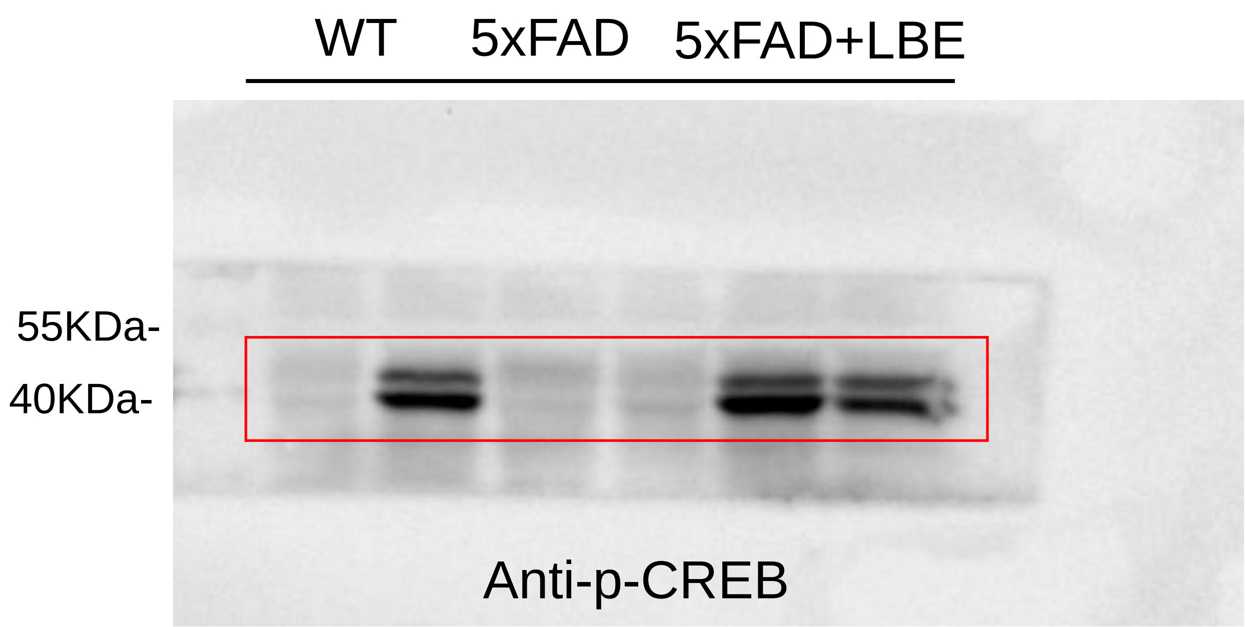

Figure8B	WT	5xFAD	LBE+5xFAD
	0.7839332	0.4564887	2.3109155
pCREB/CREB	2.7113822	0.4088167	2.0064279
	2.8877199	1.4747176	2.1885392
	1.9390552	1.049938	2.2643699
Mean	2.081	0.8475	2.193
SEM	0.4788	0.2549	0.06697

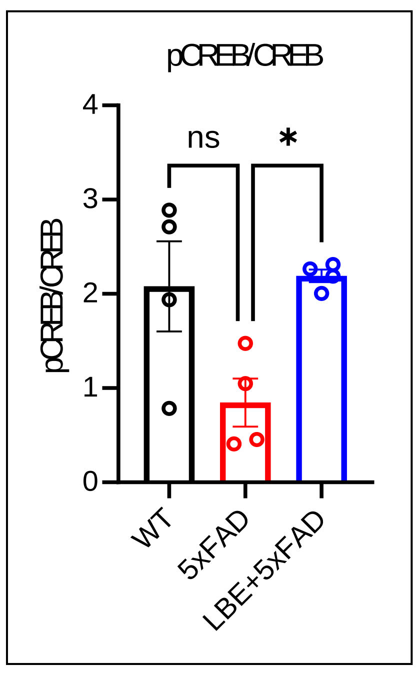

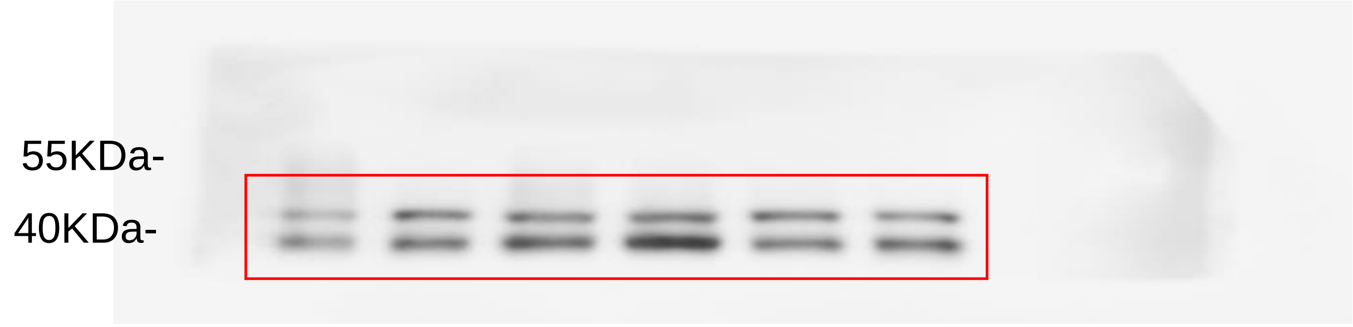

Anti-CREB

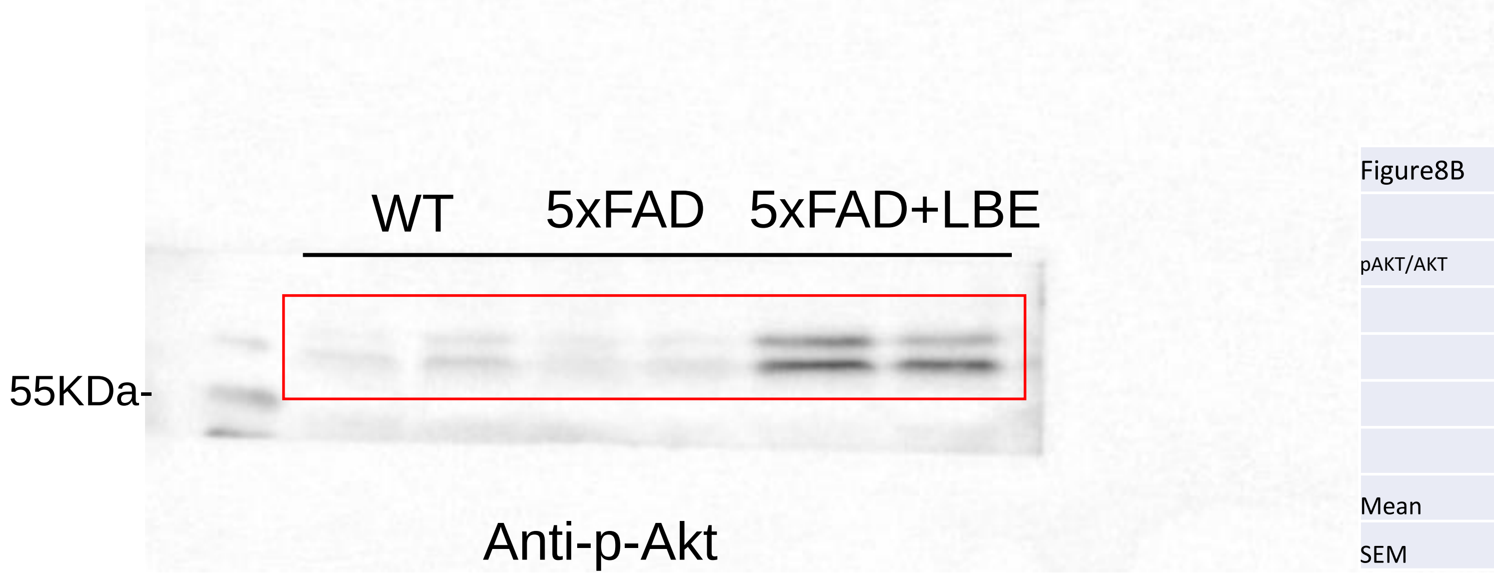

Figure8B	WT	5xFAD	LBE+5xFAD
	0.3151884	0.2380748	1.6977718
pAKT/AKT	0.8149725	0.3936409	0.7536558
	0.3507007	0.2544497	0.780928
	1.0324541	0.4911372	1.0458987
Mean	0.6283	0.3443	1.07
SEM	0.1764	0.06011	0.2195

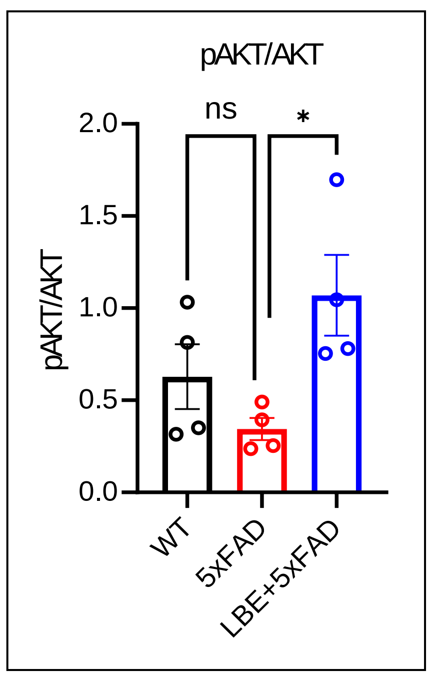

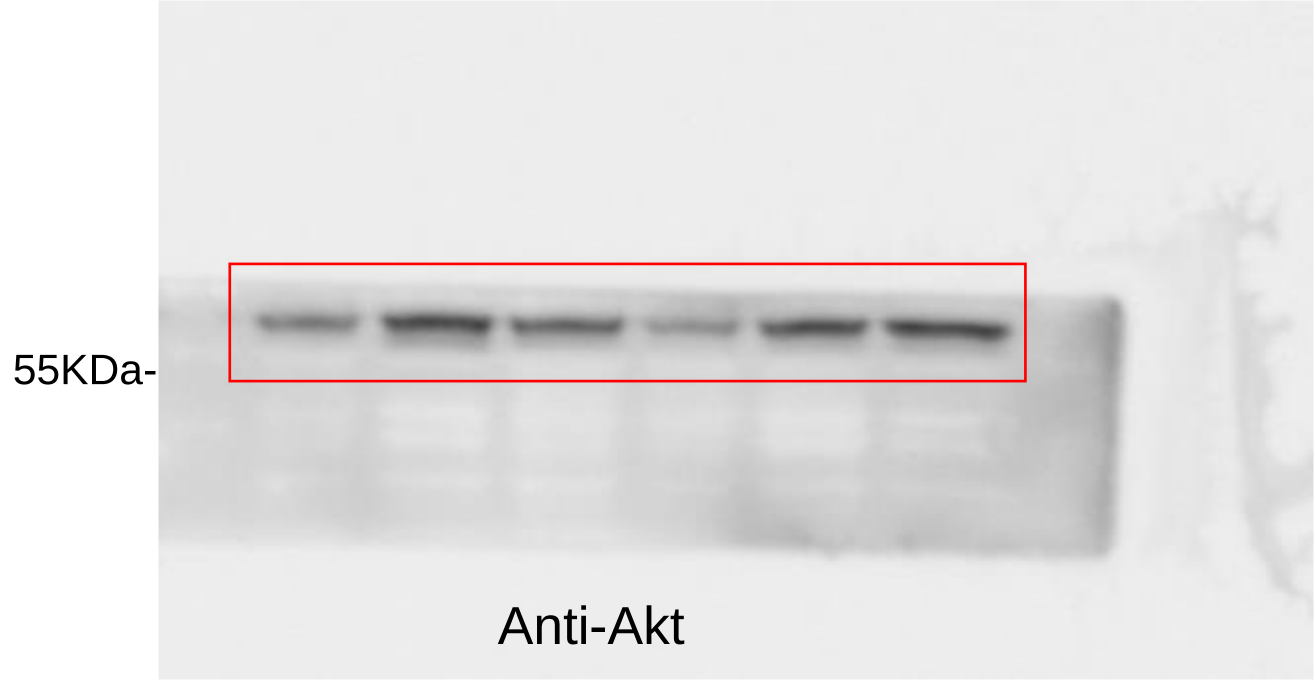

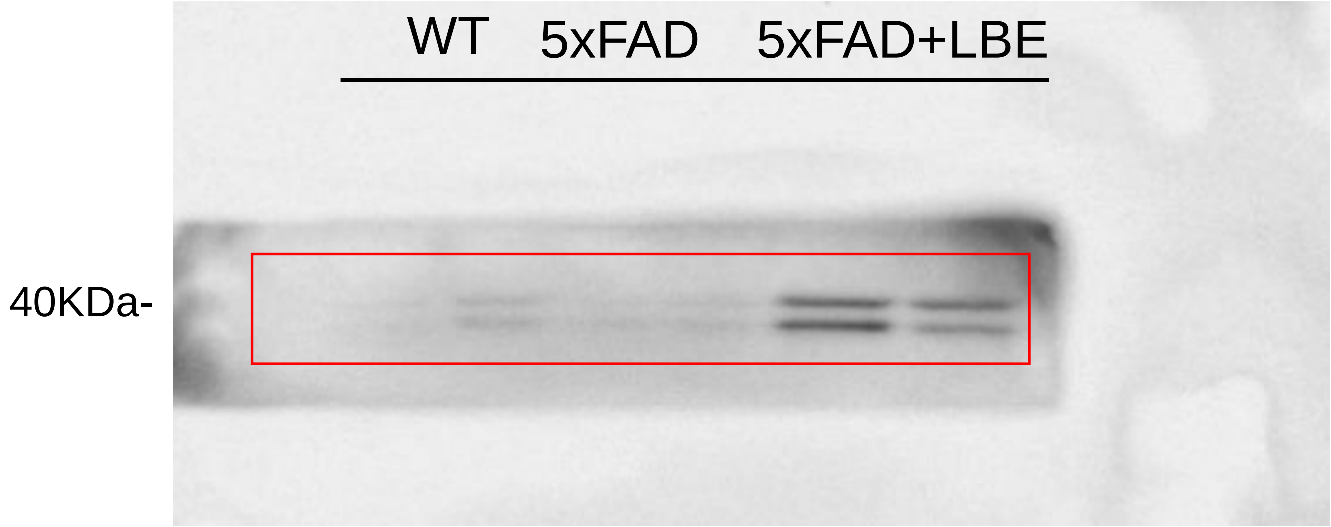

Anti-p-Erk1/2

Figure8B	WT	5xFAD	LBE+5xFAD
	0.5751597	0.683776	2.5705083
pErk/Erk	2.8923089	0.6481346	2.654845
	0.3745134	0.5790614	2.8840749
	1.3839451	0.4199429	2.8114391
Mean	1.306	0.5827	2.73
SEM	0.5719	0.05845	0.07156

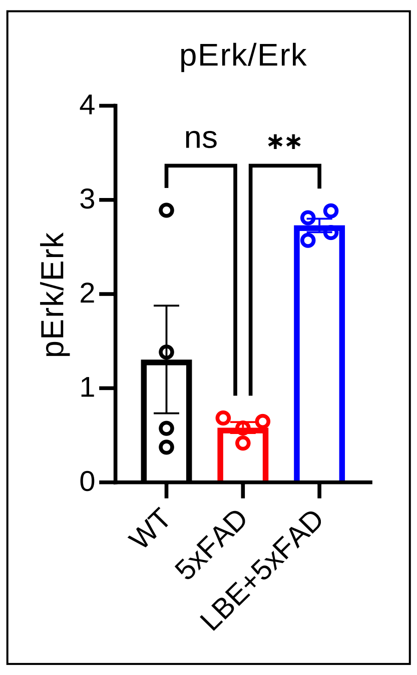

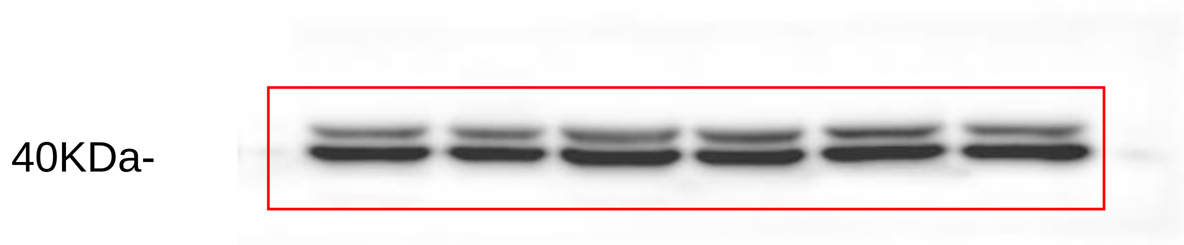

Anti-Erk1/2

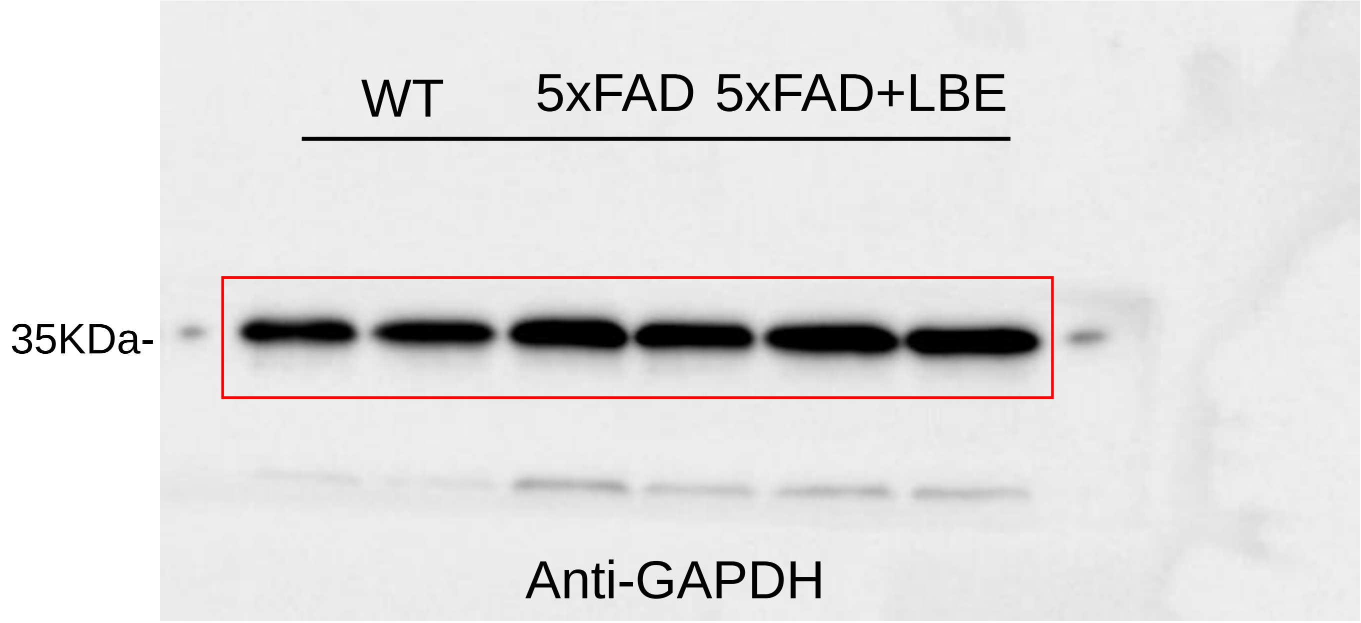

Figure8E-Spinal cord

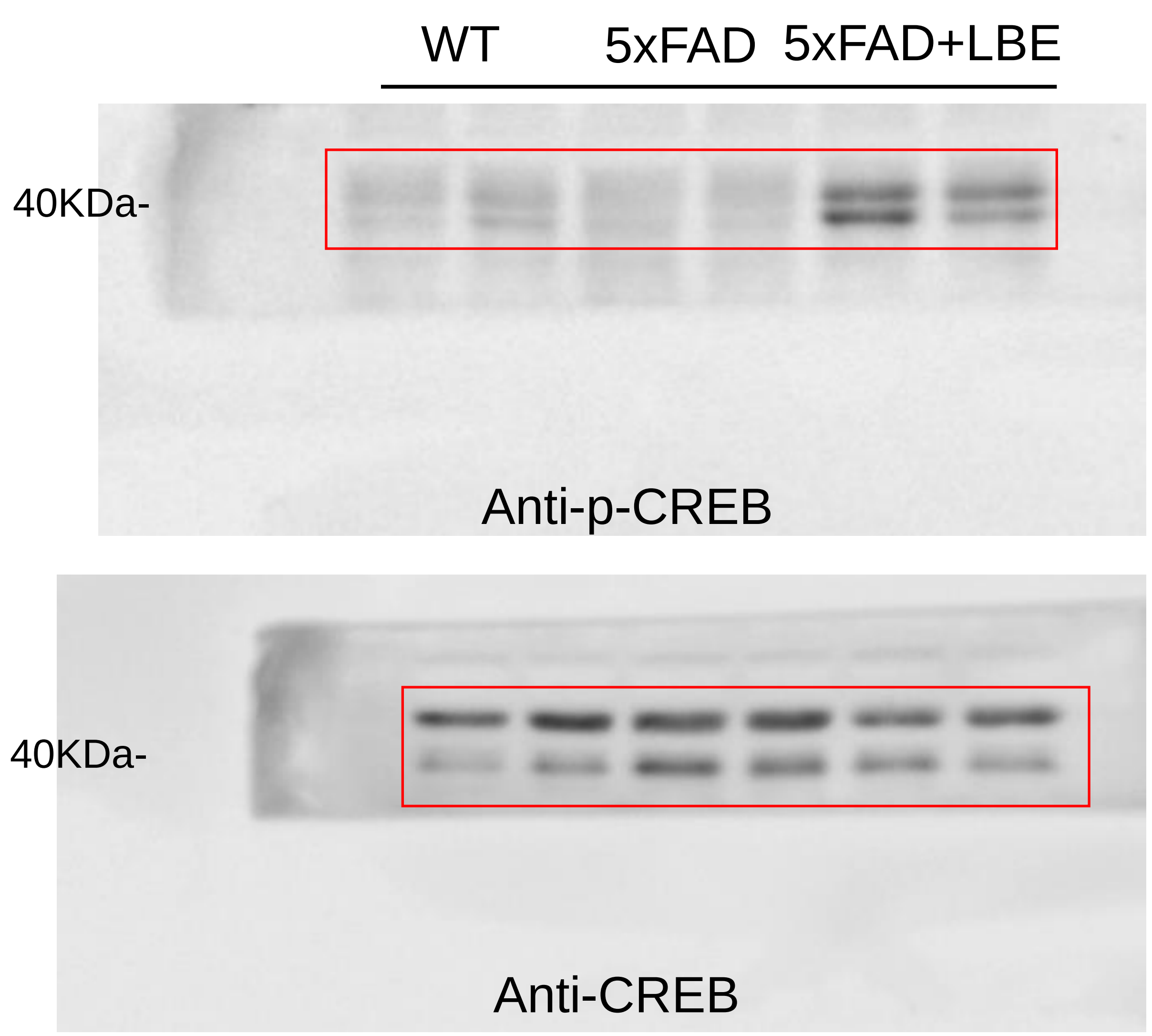

Figure8E	WT	5xFAD	LBE+5xFAD
	0.4973911	0.1574057	0.9233635
pCREB/CREB	0.5258266	0.165473	0.6848583
	0.8662193	0.2378275	0.3555498
		0.1712606	0.7982673
Mean	0.6298	0.183	0.6905
SEM	0.1185	0.0185	0.1218

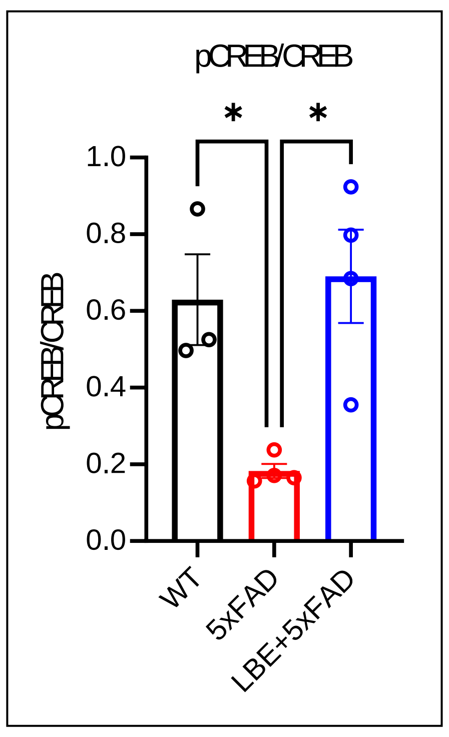

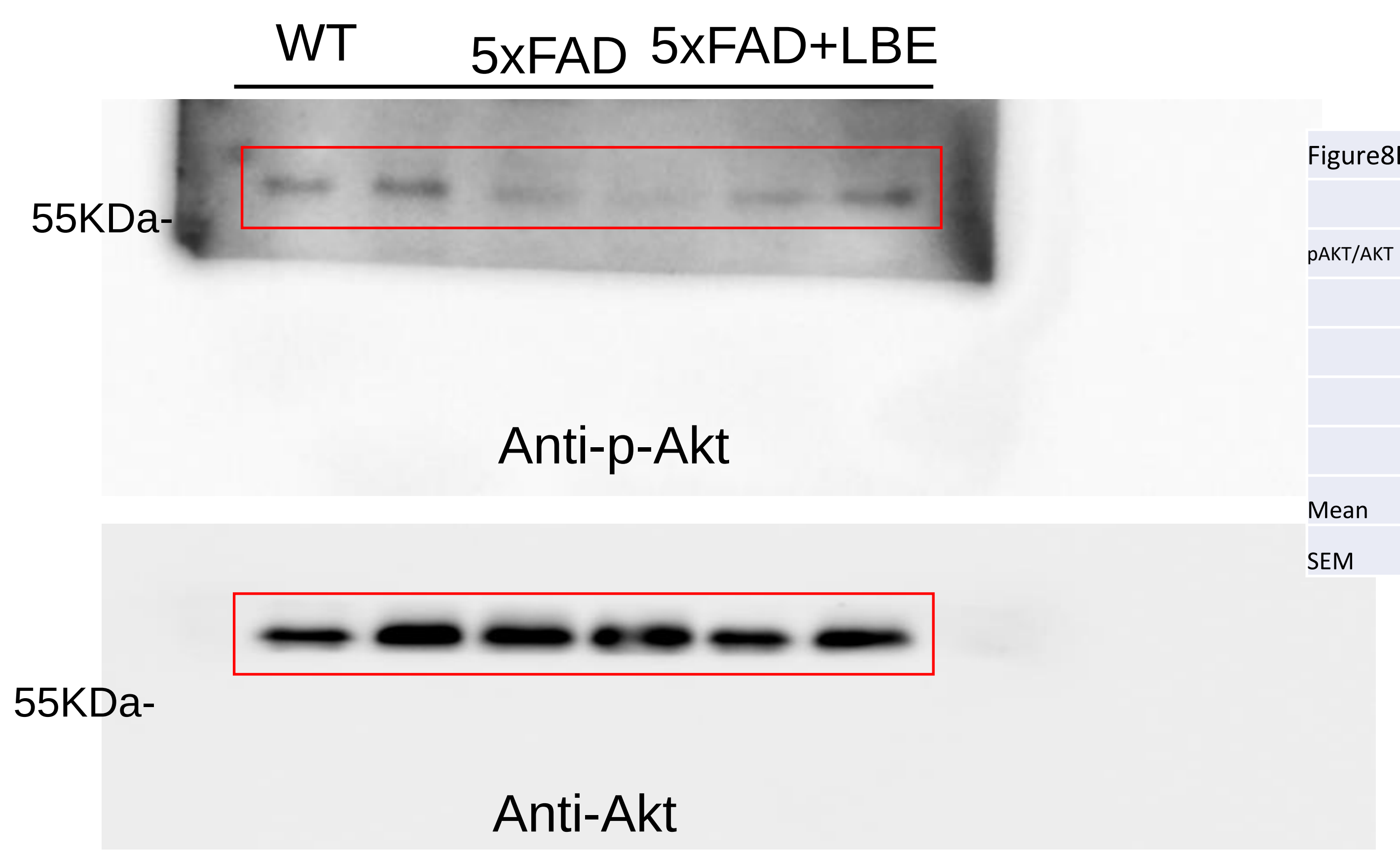

Figure8E	WT	5xFAD	LBE+5xFAD
	2.3840454	1.0493621	0.7182259
pAKT/AKT	2.1989294	0.5410423	2.2846222
	1.574844	1.4377125	1.1311293
	2.7008962	1.165442	3.5660181
Mean	2.215	1.048	1.925
SEM	0.2371	0.1877	0.6396

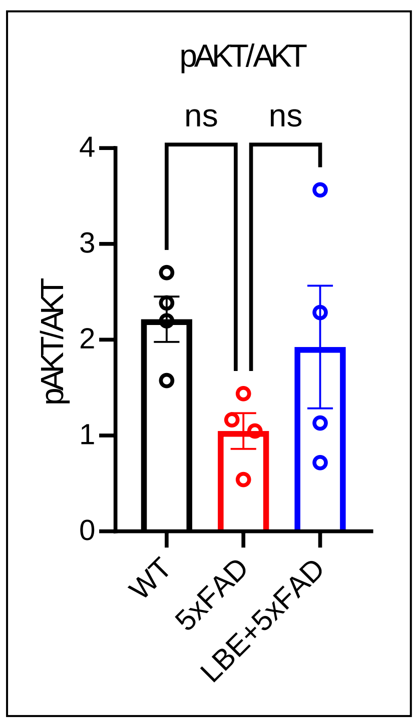

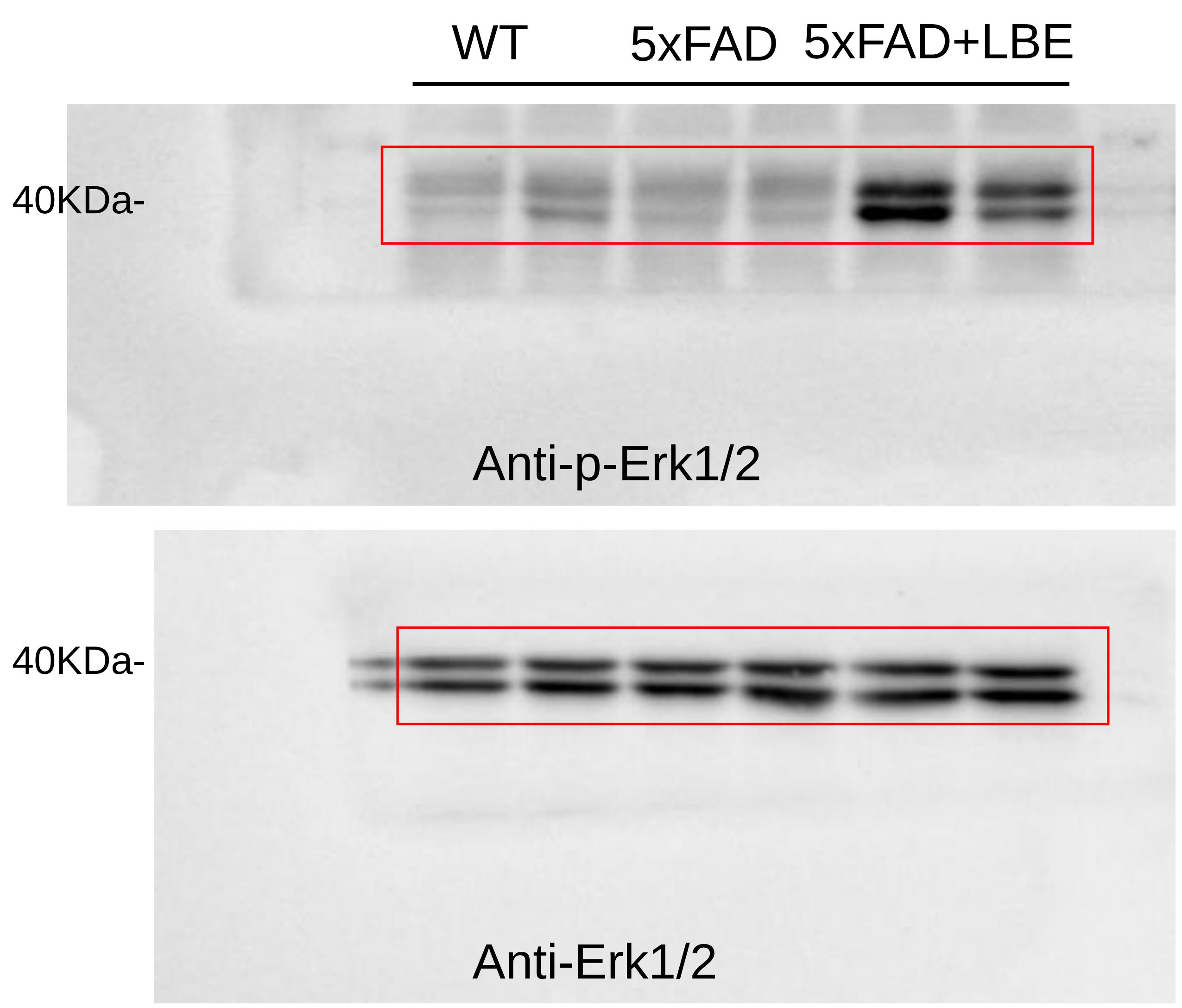

Figure8E	WT	5xFAD	LBE+5xFAD
	1.0316322	1.0230932	3.5717529
pErk/Erk	1.4008551	1.0595033	2.2490009
	1.6609217	1.1612349	1.592193
	1.8718885	0.6153223	2.0065839
Mean	1.491	0.9648	2.355
SEM	0.181	0.1201	0.4277

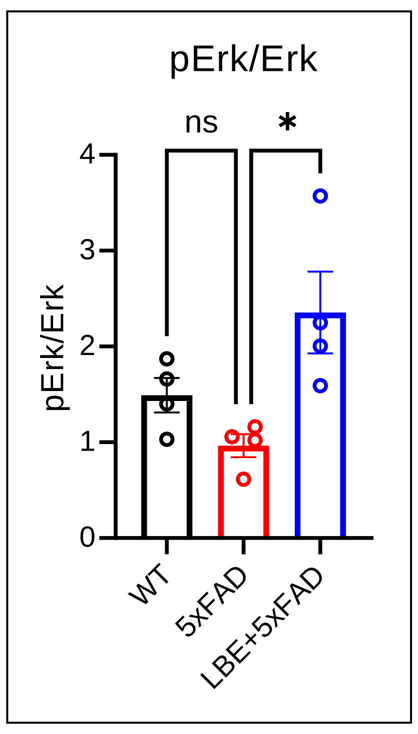

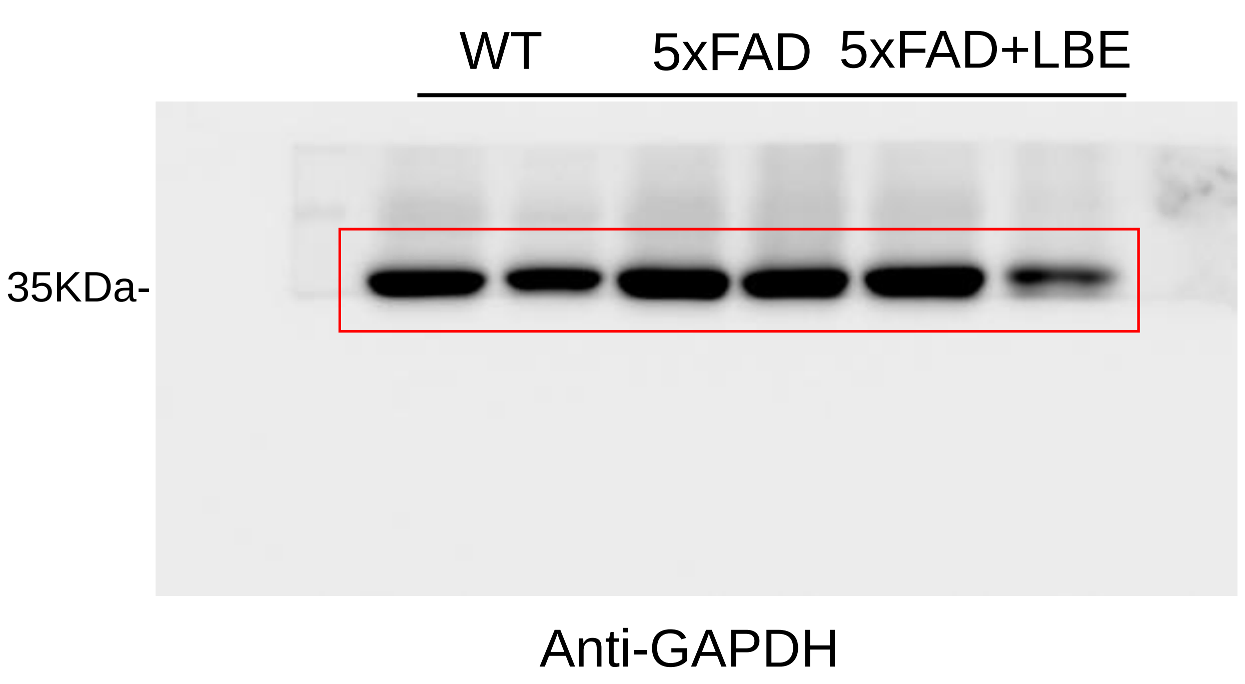

Figure8G-retina

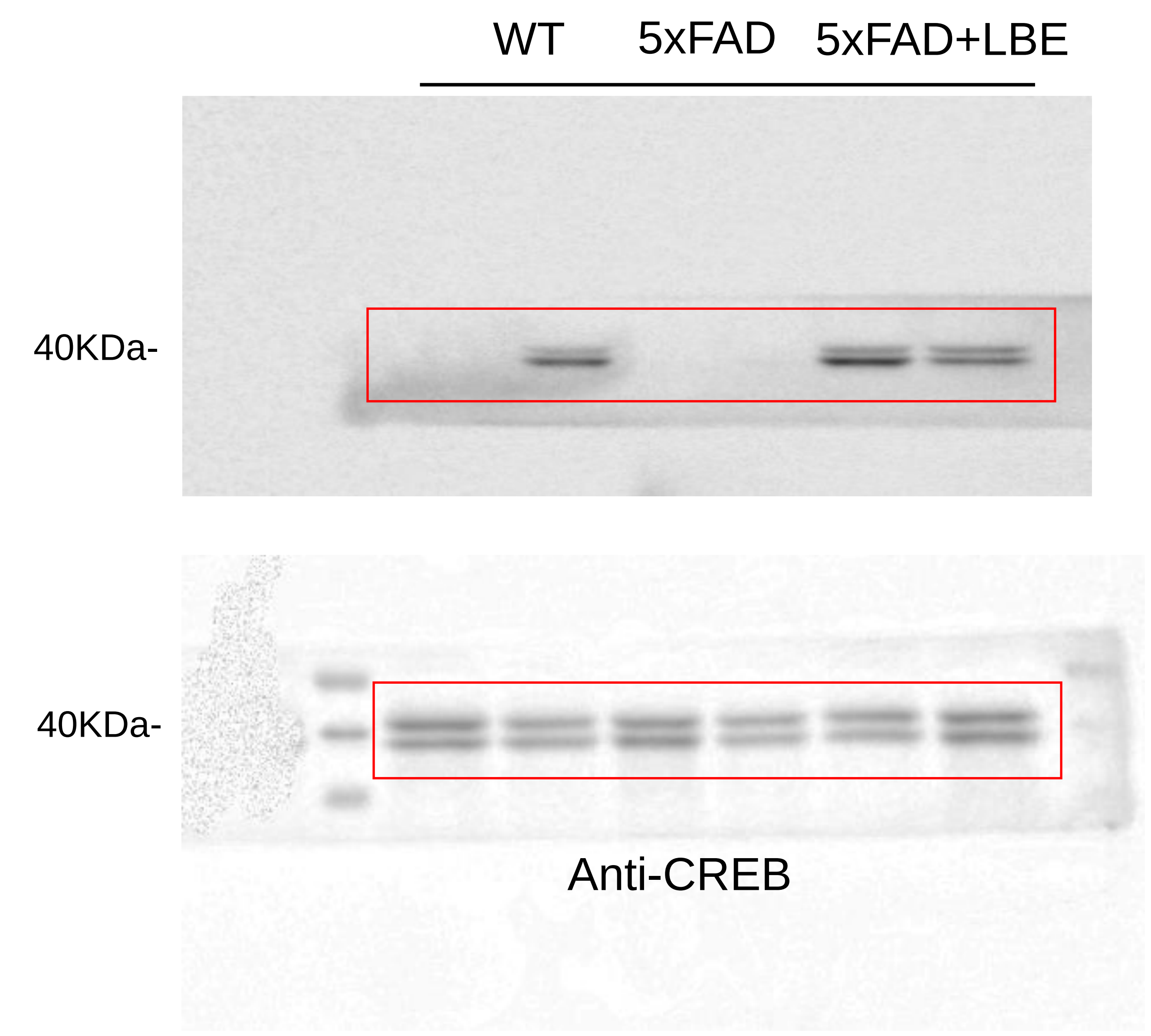

Figure8G	WT	5xFAD	LBE+5xFAD
pCREB/CREB	0.8994906	0.541321	1.7888979
	1.47218	0.5451814	1.7978353
	1.0950109	0.4702288	1.1683313
	0.5665498	0.8232906	0.8953262
Mean	1.008	0.595	1.413
SEM	0.1892	0.07802	0.2268

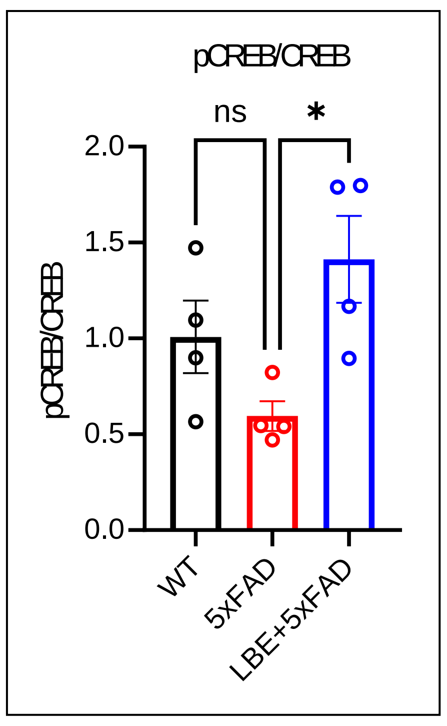

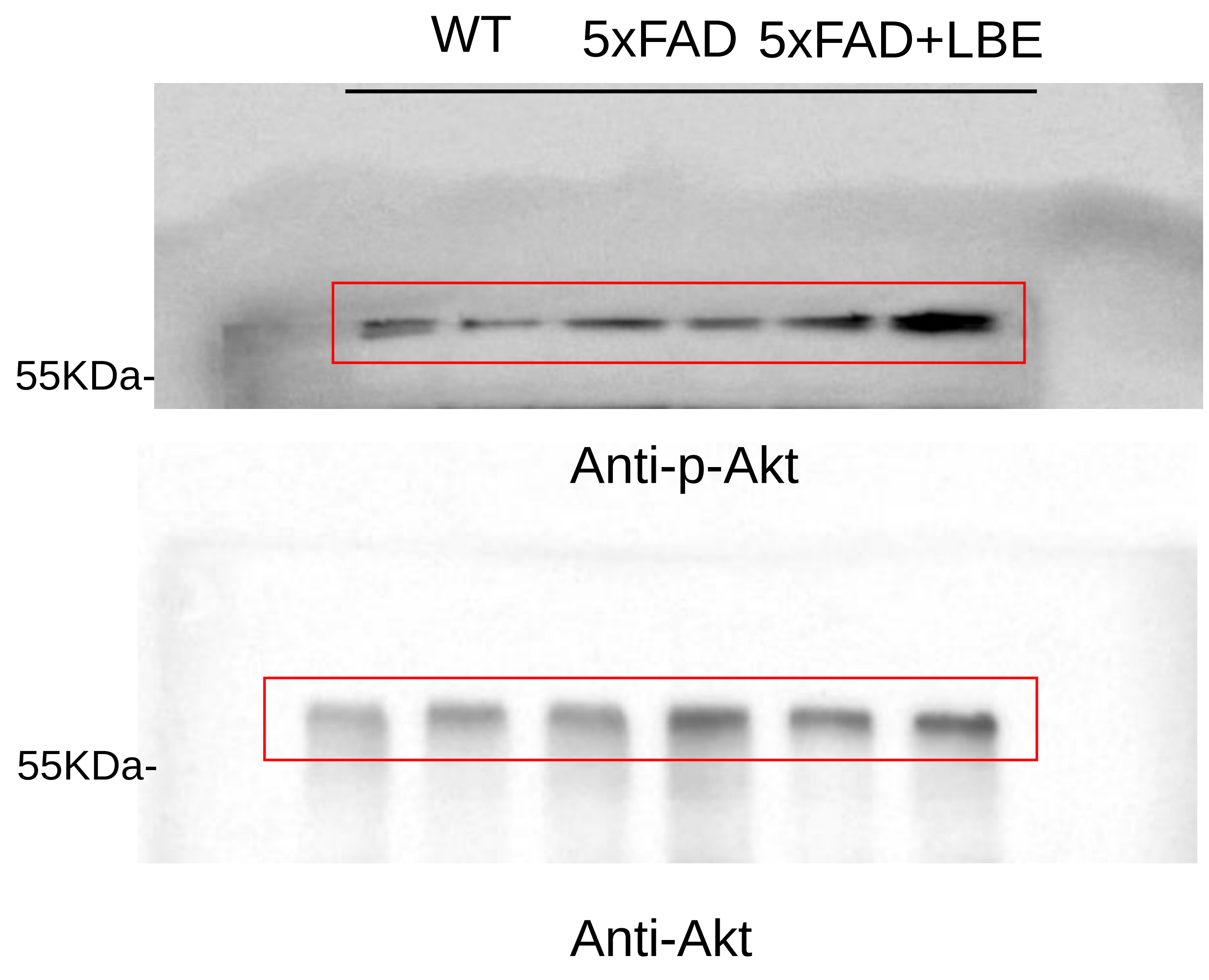

Figure8G	WT	5xFAD	LBE+5xFAD
pAKT/AKT	0.4174333	1.7112148	1.6680423
	1.8533722	0.9374567	3.0812616
	0.3098842	0.8071336	2.5921363
	1.0603147	0.2772386	1.7252961
Mean	0.9103	0.9333	2.267
SEM	0.3554	0.296	0.3441

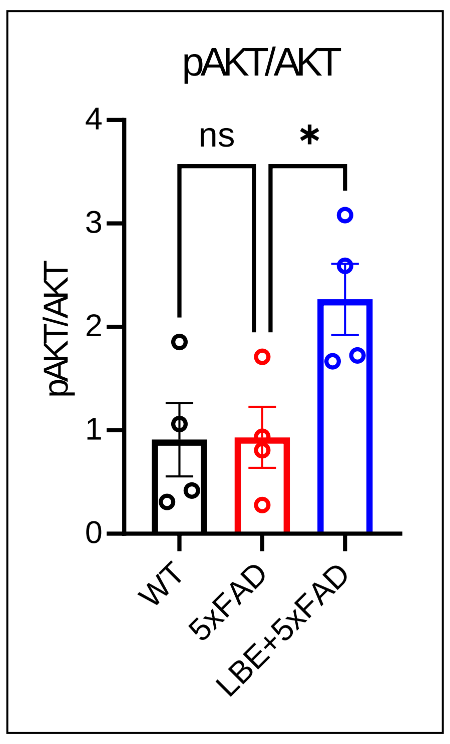

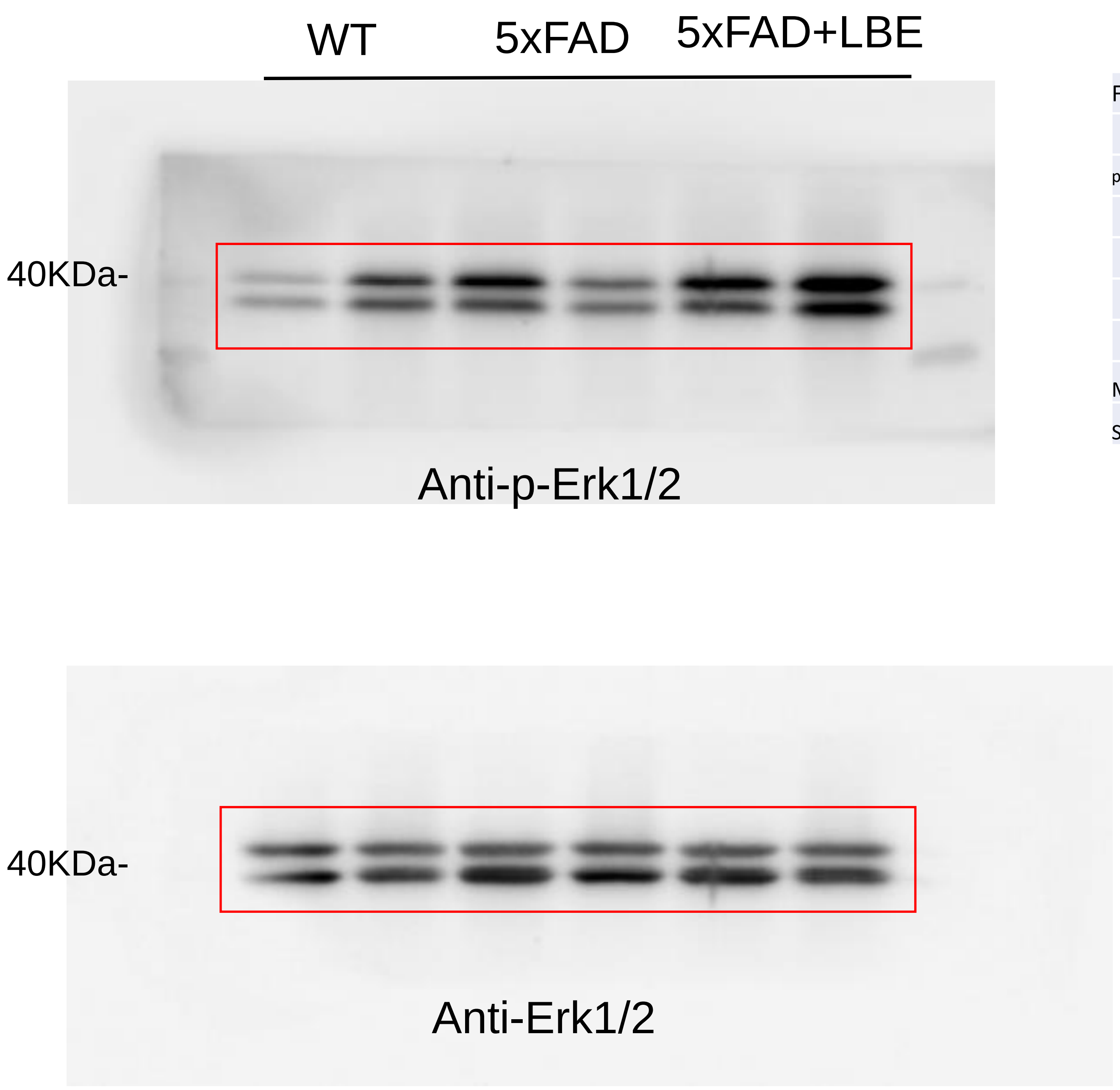

Figure8G	WT	5xFAD	LBE+5xFAD
pErk/Erk	0.6852574	0.8724561	1.0291002
	1.0336898	0.5764276	1.5819102
	1.218592	0.7620252	1.5452734
	1.093081	1.1091394	1.8642559
Mean	1.008	0.83	1.505
SEM	0.1142	0.1113	0.1739

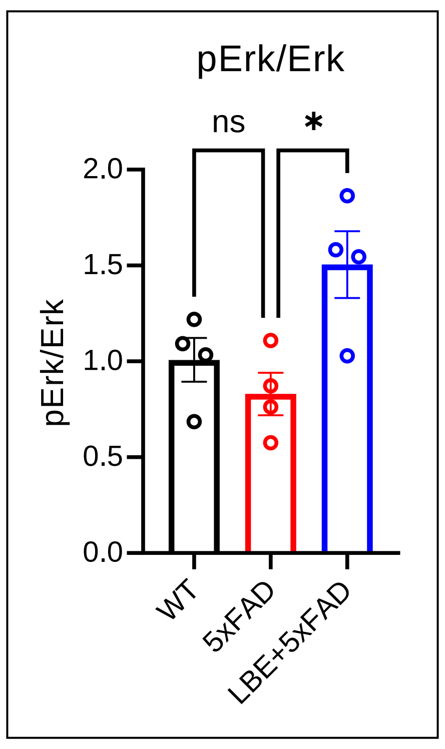

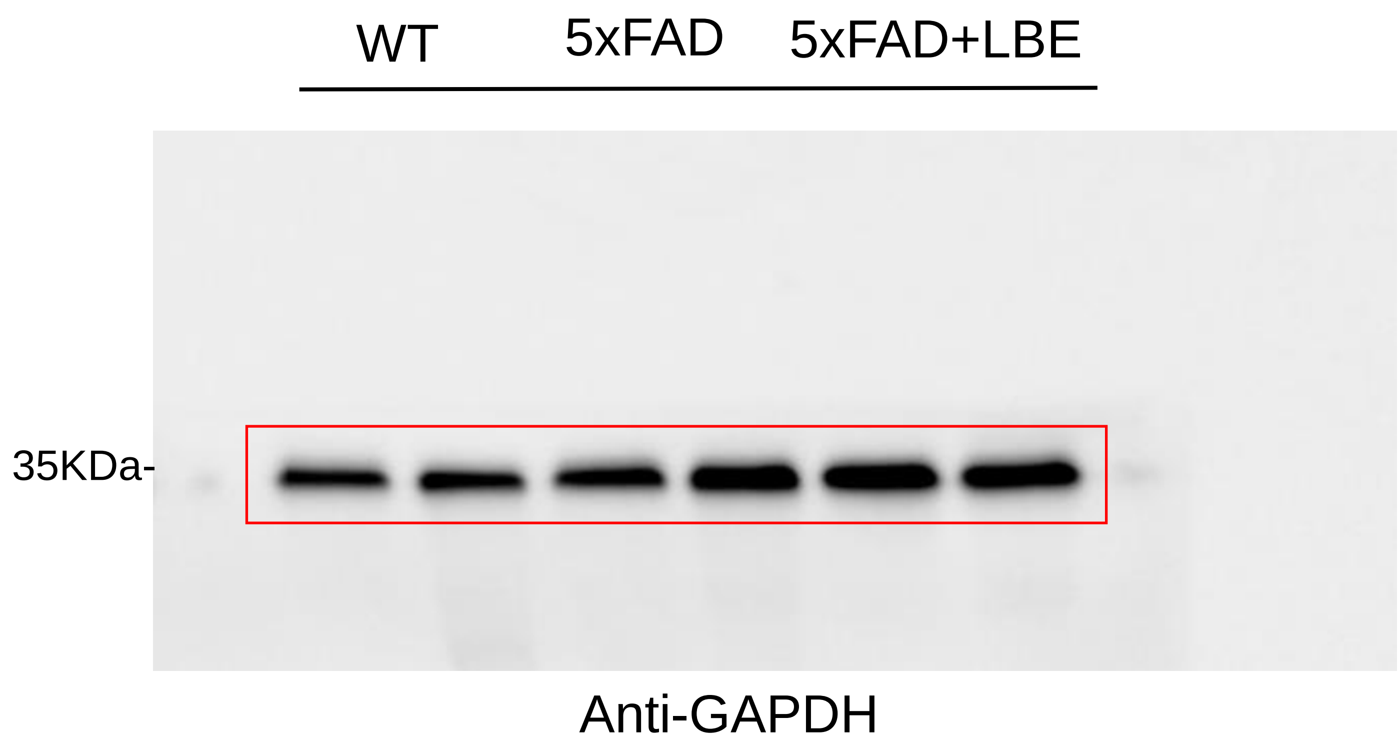

SFigure4-Hippocampus

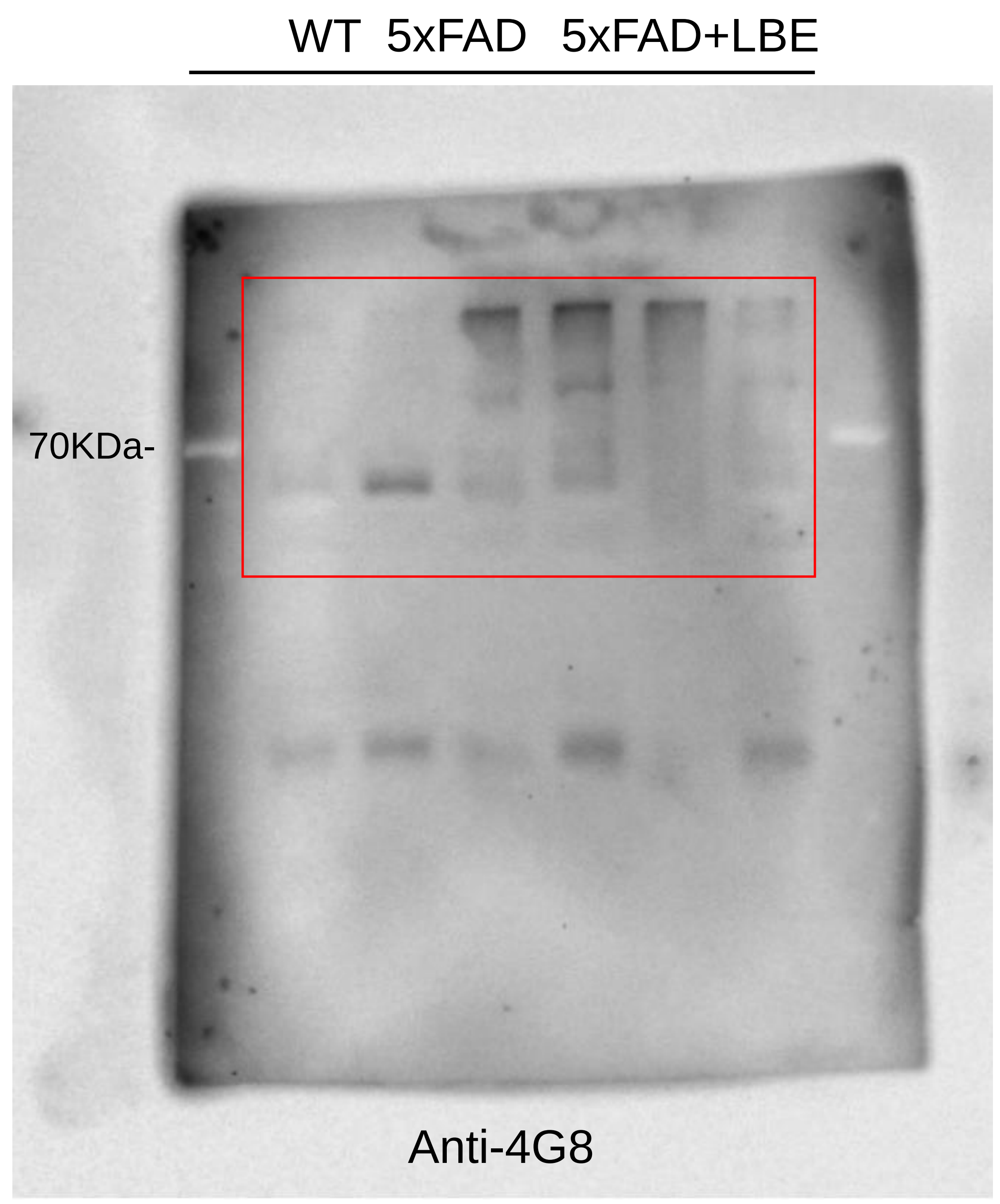

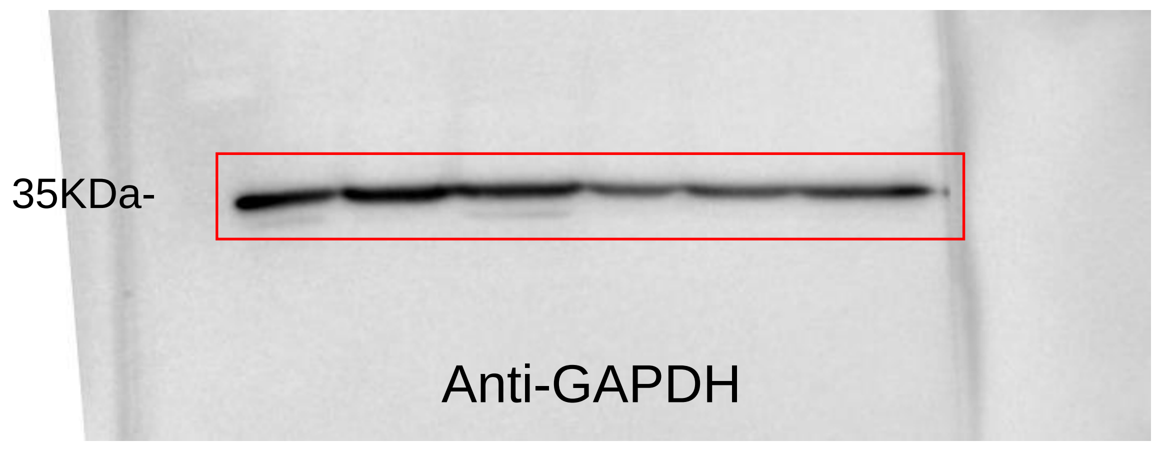

SFigure2A	WT	5xFAD	LBE+5xFAD
	1.003516	1.834932	1.096032
4G8/GAPDH	0.732315	1.847597	1.403044
	0.727873	1.410983	1.158024
	1.361336	1.796112	0.931392
Mean	0.9563	1.722	1.147
SEM	0.1496	0.1044	0.09779

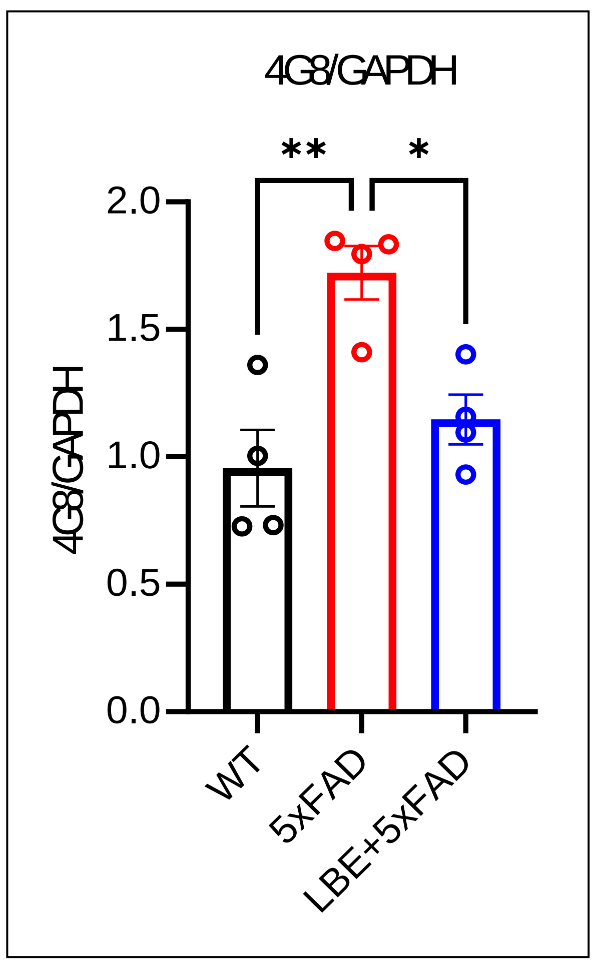

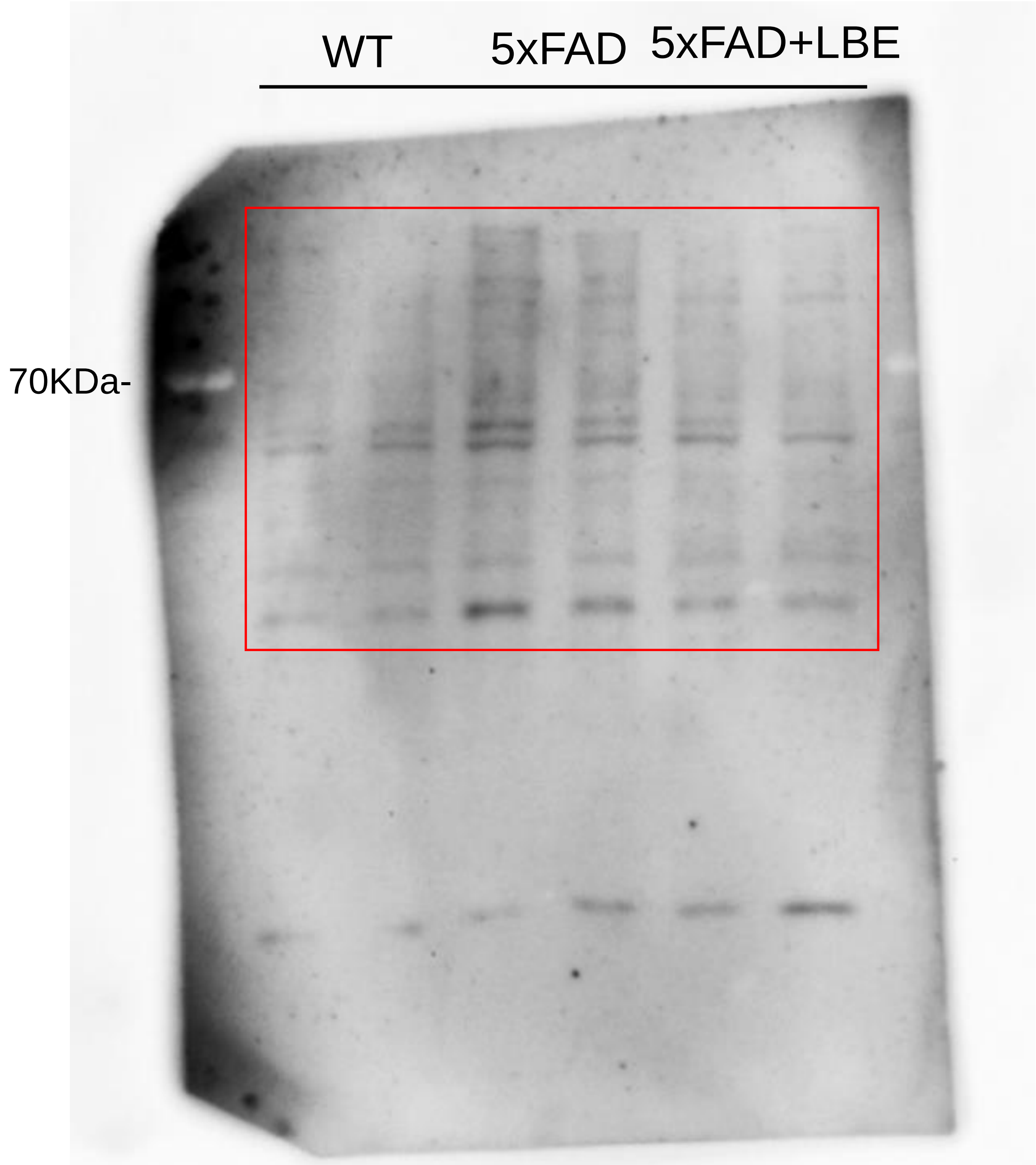

Anti-Aβ1-42

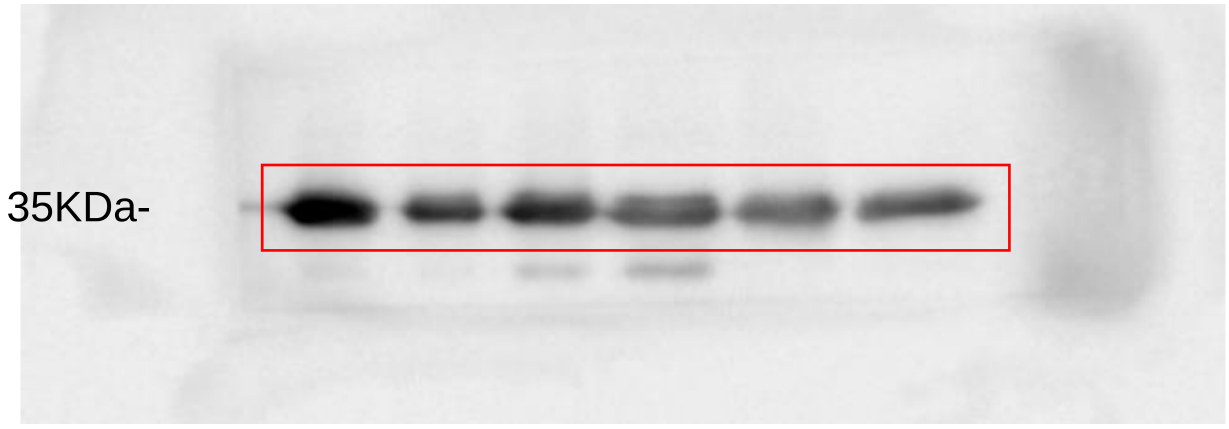

Anti-GAPDH

SFigure2B	WT	5xFAD	LBE+5xFAD
	0.431283	1.05431	0.602768
Aβ1-42/GAPDH	0.564715	0.933589	0.337892
	0.854495	1.159593	0.567553
	0.404017	0.970192	0.452922
Mean	0.5636	1.029	0.4903
SEM	0.1031	0.05021	0.06003

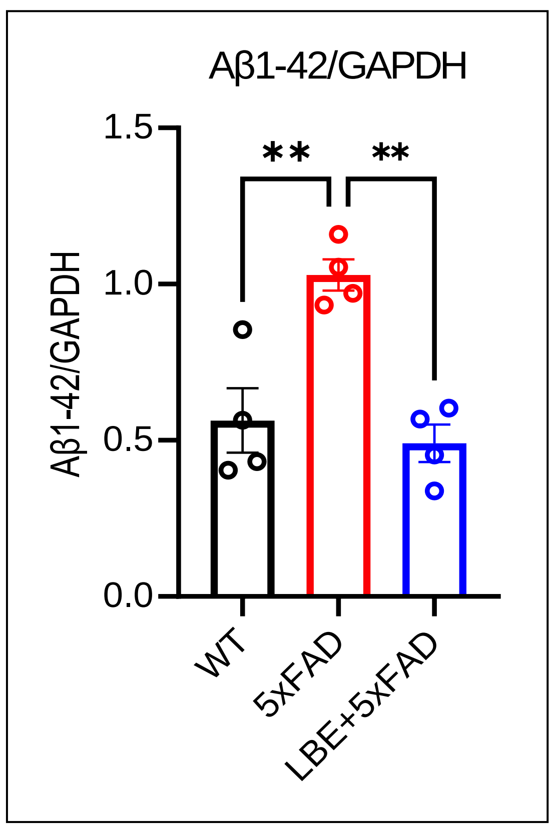

SFigure4-Cortex

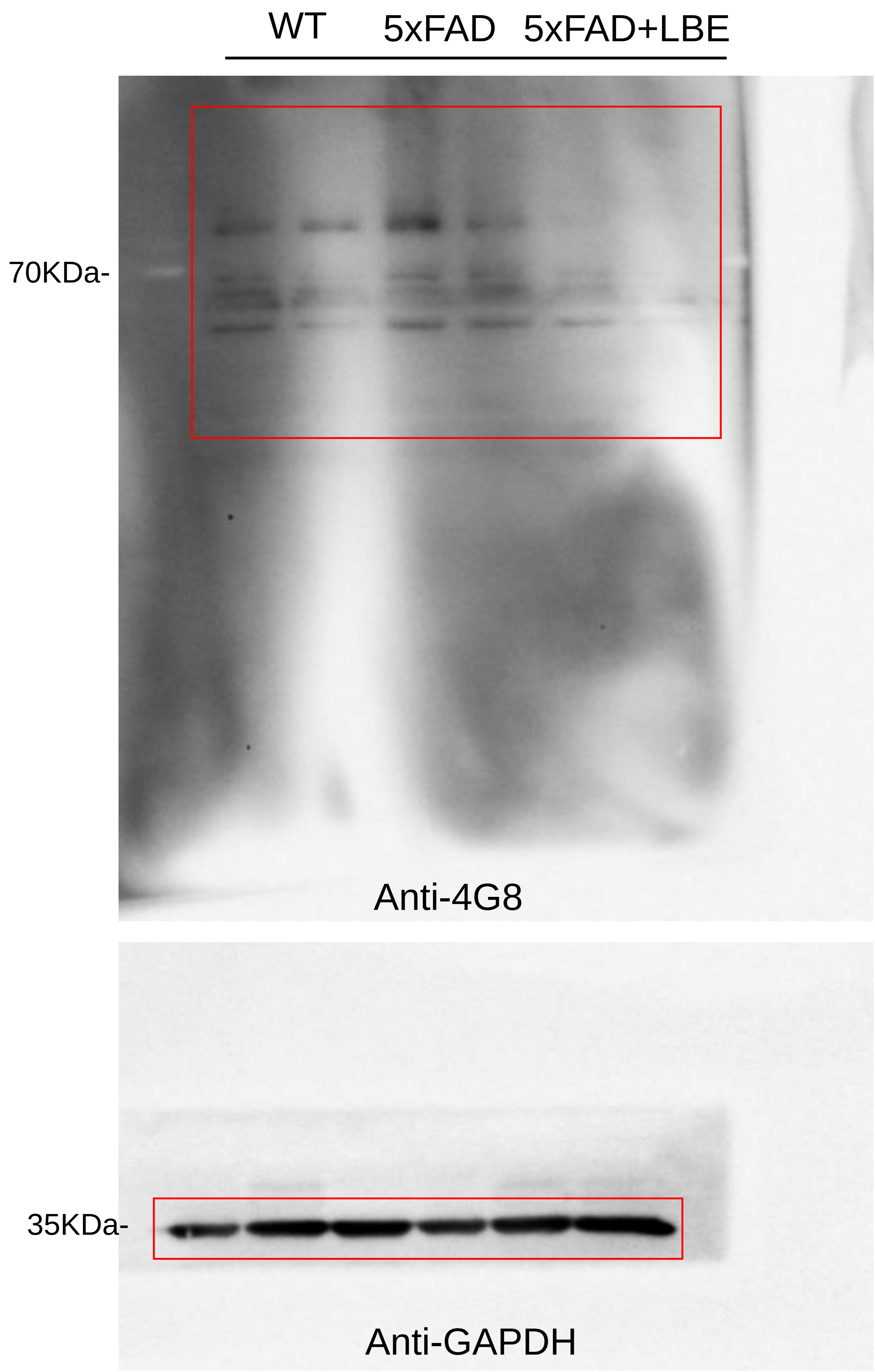

SFigure2C	WT	5xFAD	LBE+5xFAD
	0.805175	0.848118	0.489862
4G8/GAPDH	0.571301	1.166874	0.419853
	0.723724	1.023631	0.375844
	0.547742	1.254803	0.806809
Mean	0.662	1.073	0.5231
SEM	0.06164	0.08892	0.09744

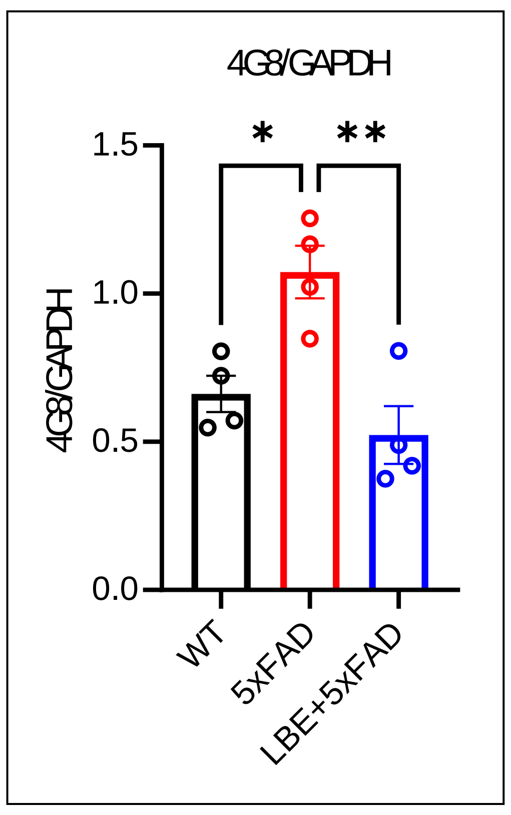

SFigure4-Spinal cord

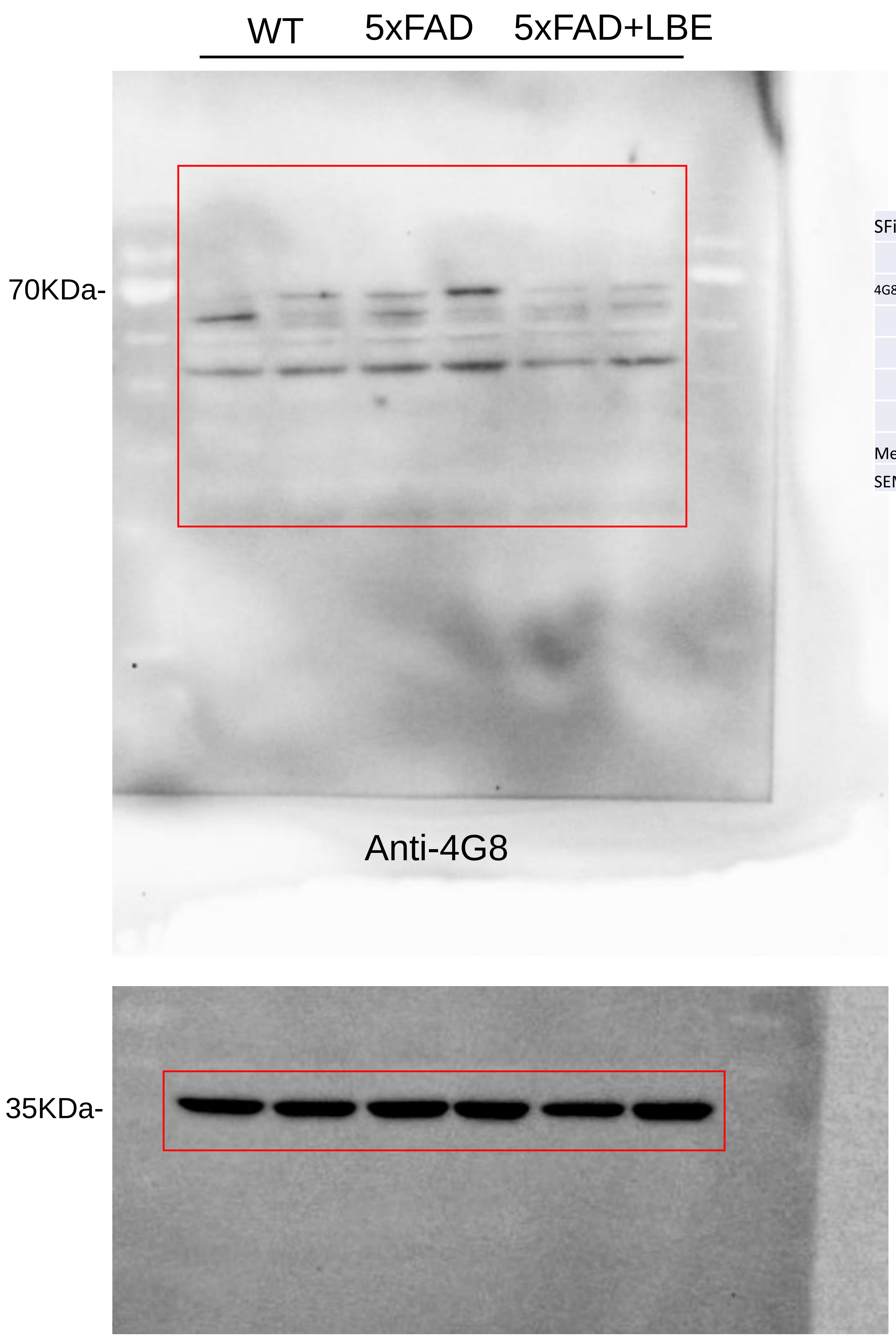

Anti-GAPDH

SFigure2D	WT	5xFAD	LBE+5xFAD
	0.892231	0.96842	0.454397
4G8/GAPDH	0.702515	0.802546	0.516617
	0.979822	1.074819	0.801225
	1.022055	1.18048	0.844777
Mean	0.8992	1.007	0.6543
SEM	0.0709	0.08061	0.09865

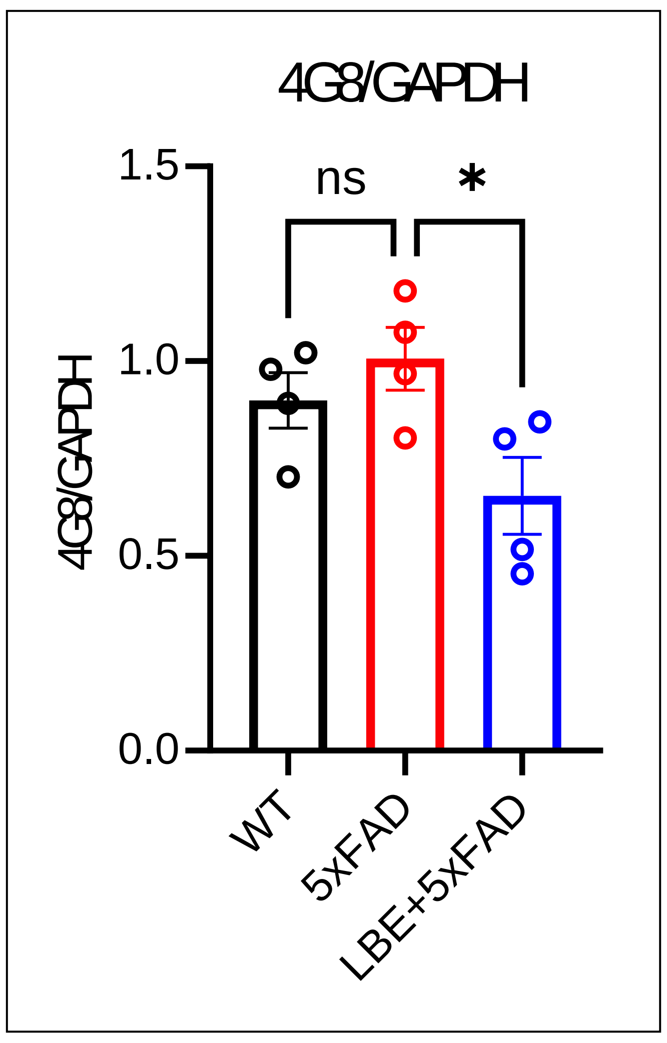

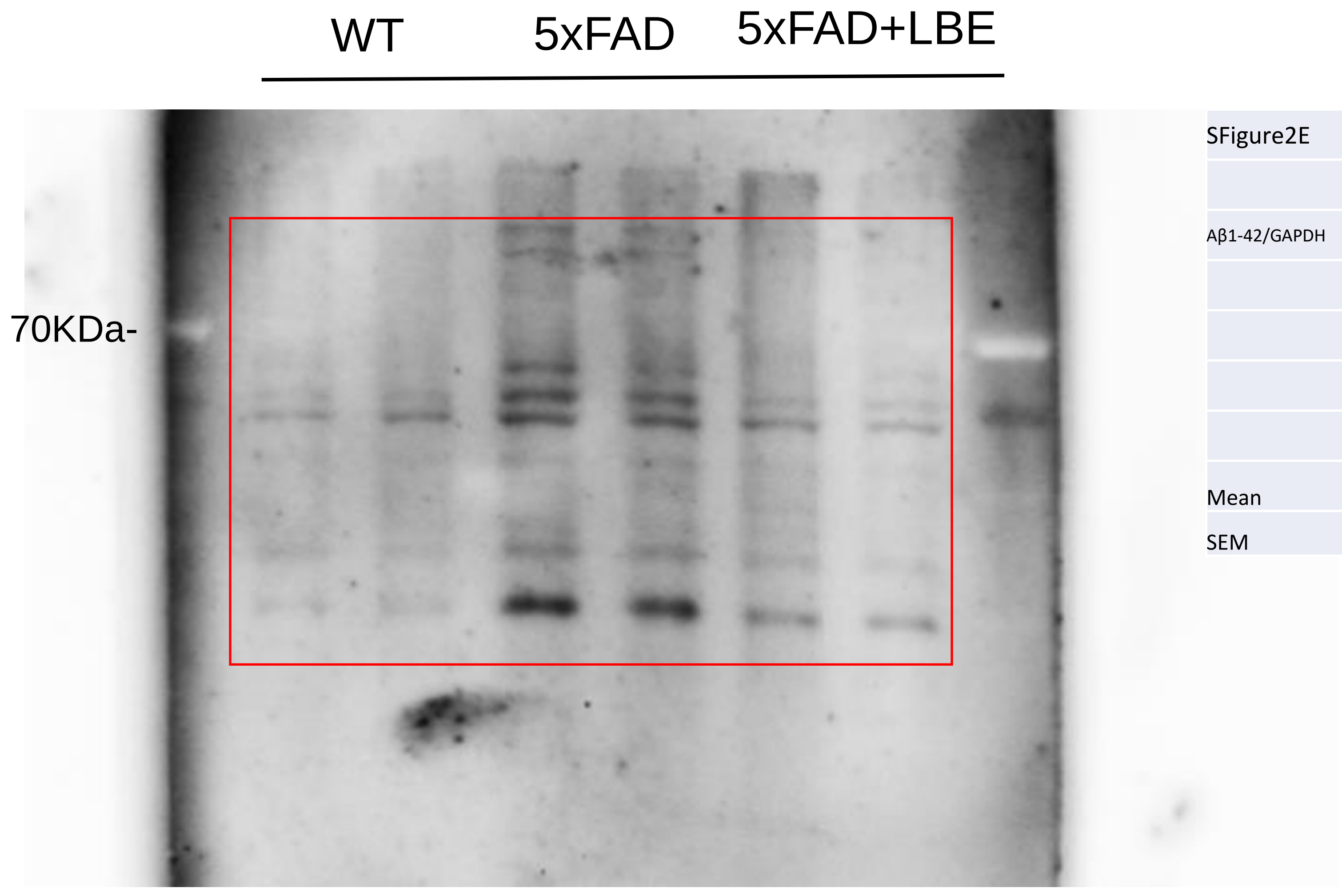

Anti-Aβ1-42

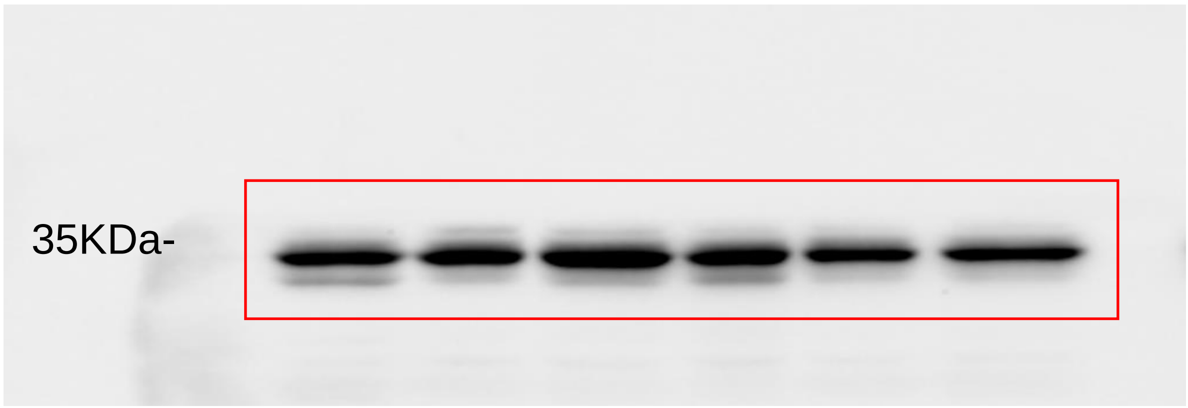

Anti-GAPDH

SFigure2E	WT	5xFAD	LBE+5xFAD
	0.709296	1.183982	0.886828
Aβ1-42/GAPDH	0.624881	1.14357	1.159115
	0.437709	1.390445	0.849019
	0.680566	1.382348	0.431105
Mean	0.6131	1.275	0.8315
SEM	0.06104	0.06481	0.1503

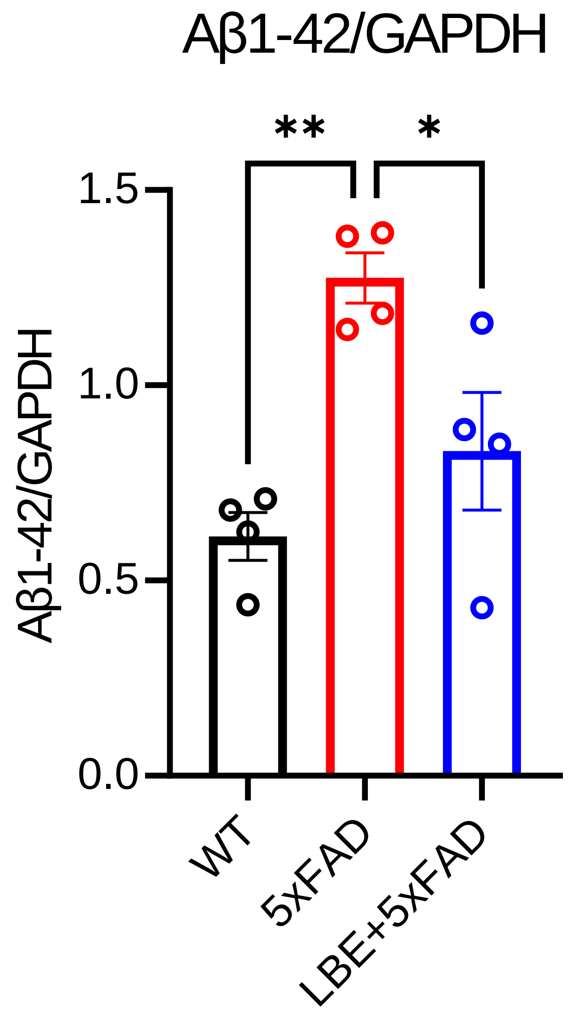

SFigure4-Retina

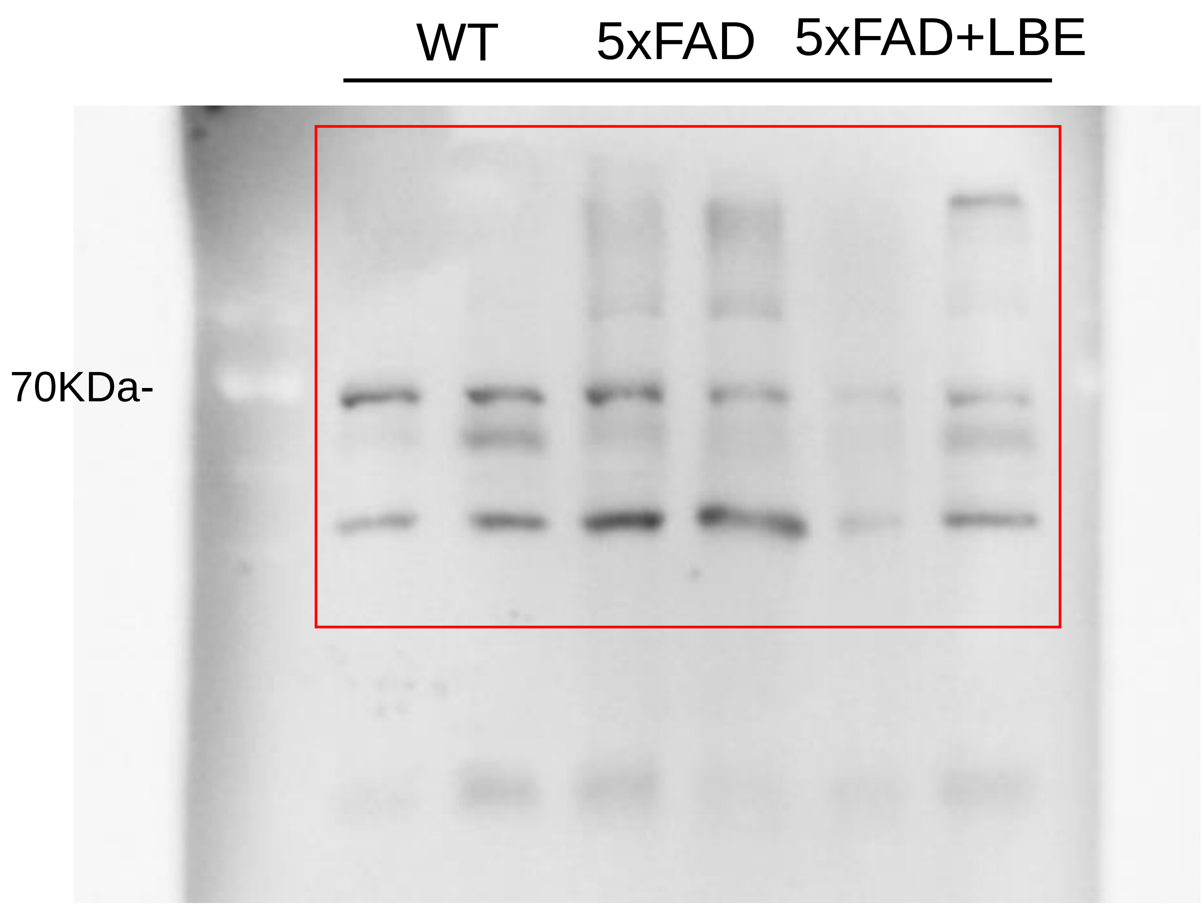

Anti-4G8

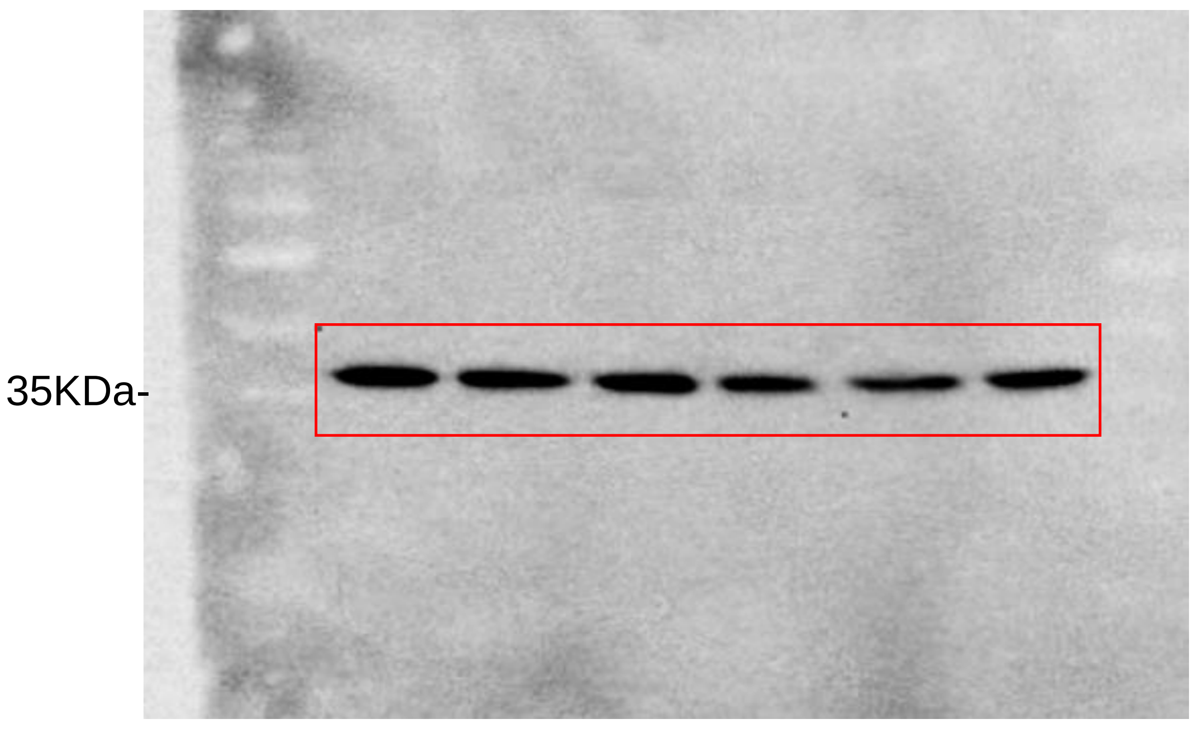

Anti-GAPDH

SFigure2F	WT	5xFAD	LBE+5xFAD
	0.567386	1.012181	0.708816
4G8/GAPDH	0.89945	0.93038	0.761061
	0.71579	0.954302	0.777957
	0.83855	1.166408	0.724831
Mean	0.7553	1.016	0.7432
SEM	0.07336	0.05305	0.01593

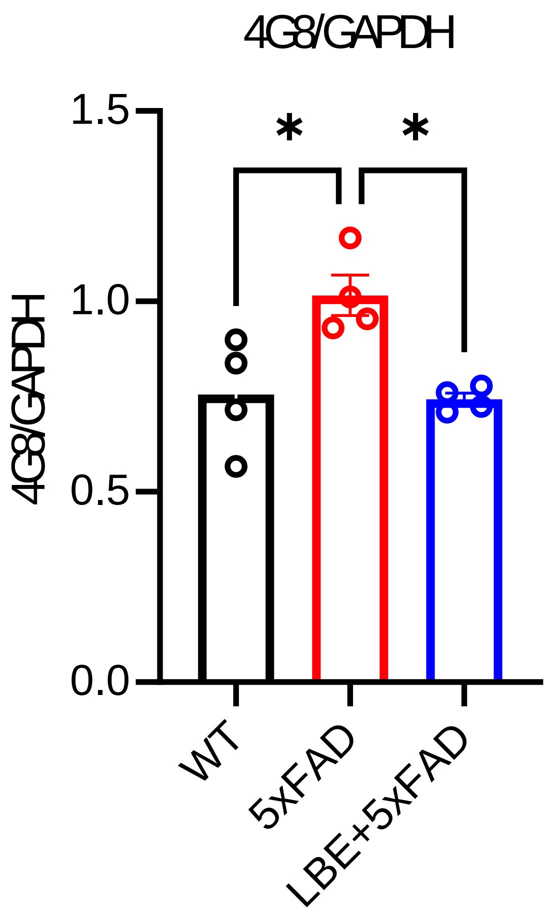

SFig5-SYP-WB

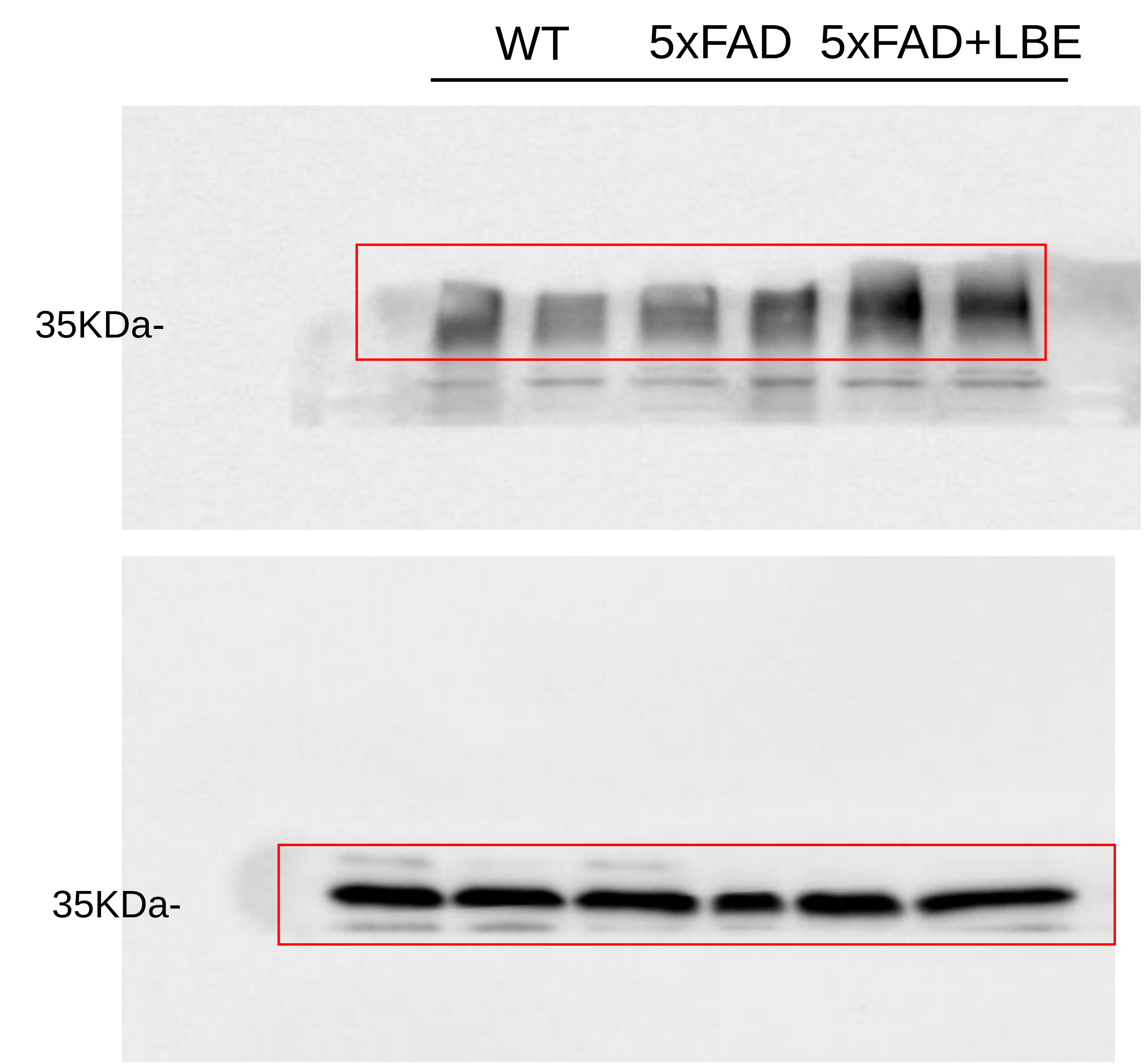

Anti-GAPDH

Sfigure-7	WT	5xFAD	LBE+5xFAD
	1.977642	1.716191	2.99564
SYP/GAPDH	1.966594	2.171409	2.756436
	1.451237	0.97021	2.660797
	1.772569	1.187855	2.71811
Mean	1.792	1.511	2.783
SEM	0.123	0.27	0.07364

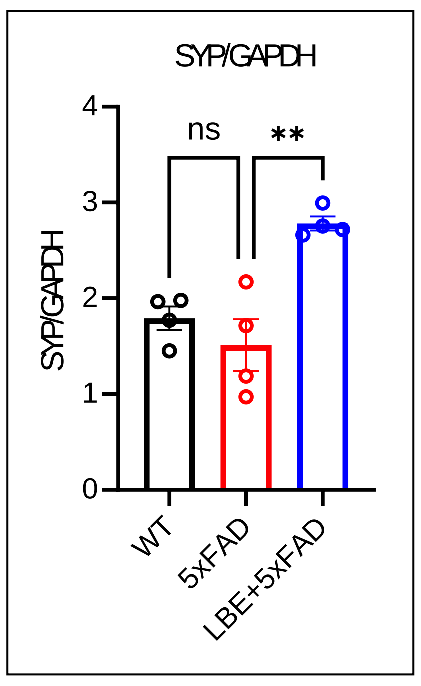

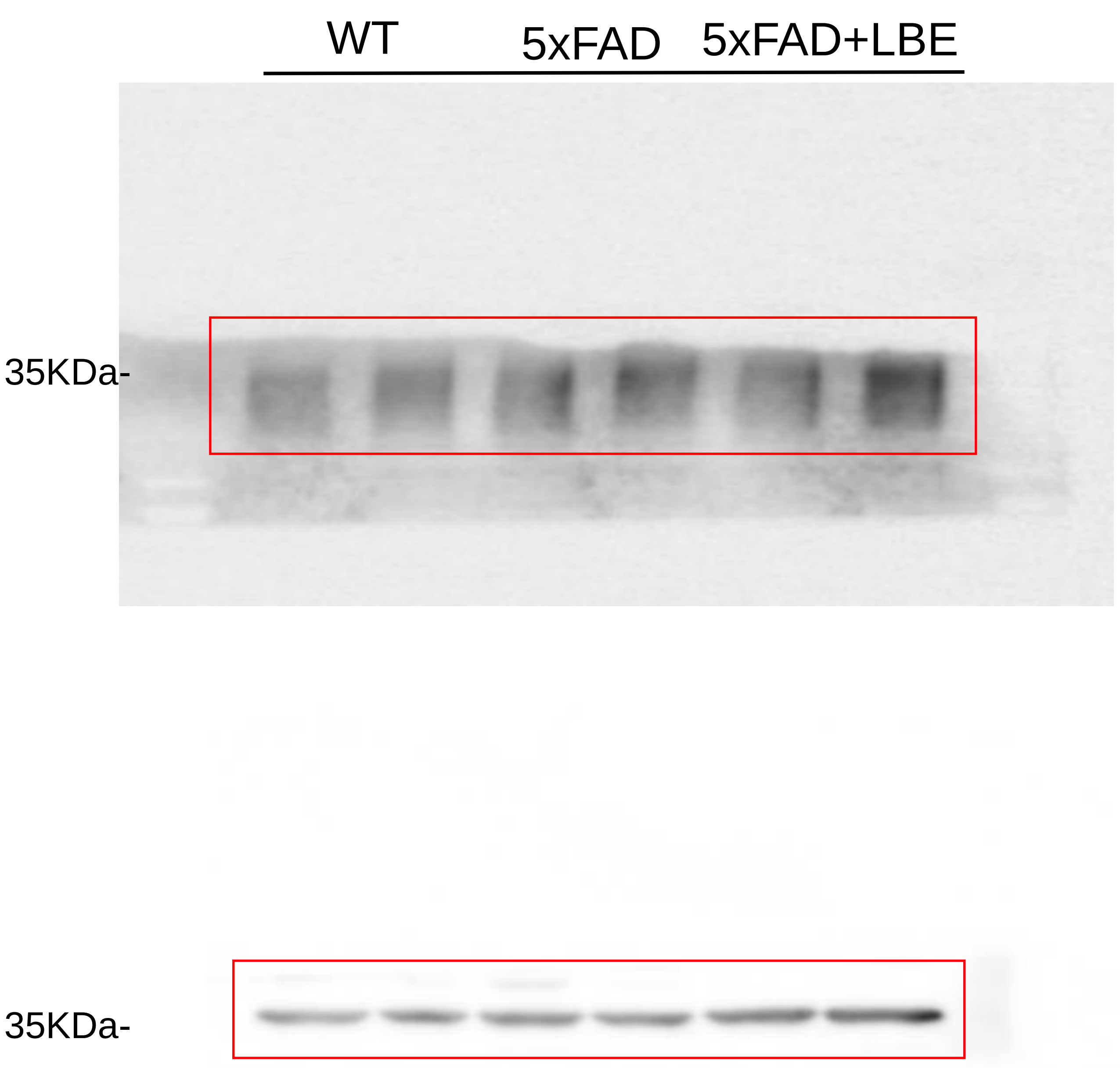

Anti-GAPDH

Sfigure-7	WT	5xFAD	LBE+5xFAD
	2.324057	2.341814	2.408365
SYP/GAPDH	2.492805	1.989089	3.357561
	2.231269	0.837634	1.10583
	4.152633	0.792042	2.83746
Mean	2.8	1.49	2.427
SEM	0.4541	0.3966	0.4813

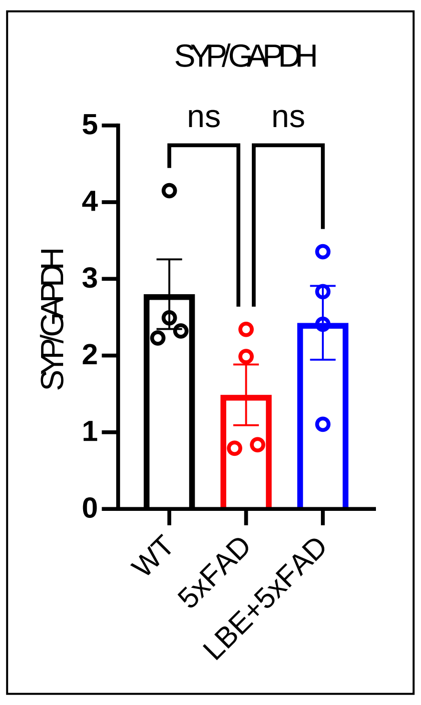

Supplement: Supplementary file 2 — Data S1. Supporting Information. [file CNS-30-e70123-s001.pdf]
